# Catalytic Photoredox C–H Arylation of 4-Oxo-4*H*-pyrido[1,2-*a*]pyrimidine-3-diazonium Tetrafluoroborates and Related Heteroaryl Diazonium Salts

**DOI:** 10.1021/acs.joc.3c01517

**Published:** 2023-09-07

**Authors:** Kris Antolinc, Helena Brodnik, Uroš Grošelj, Bogdan Štefane, Nejc Petek, Jurij Svete

**Affiliations:** Faculty of Chemistry and Chemical Technology, University of Ljubljana, Večna Pot 113, SI-1000 Ljubljana, Slovenia

## Abstract

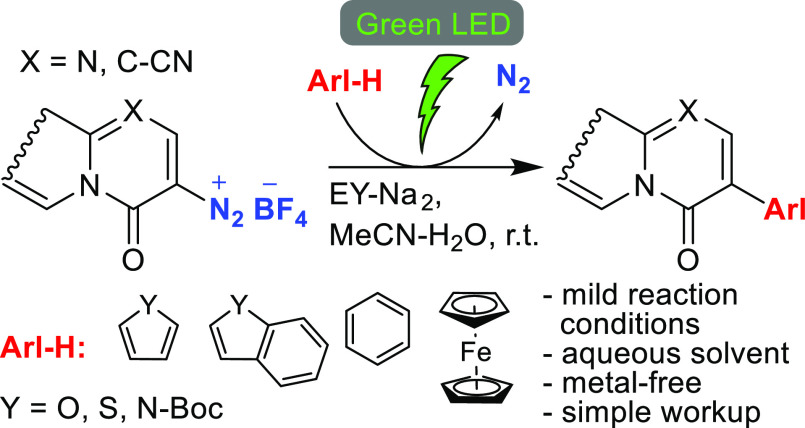

Irradiation of mixtures of title diazonium salts and heteroarenes with green light (510 nm) in the presence of eosin Y disodium salt (**EY-Na**_**2**_) as a photocatalyst furnished the corresponding arylation products in 8–63% yields. The proposed photocatalytic cycle is analogous to that proposed previously for closely related photoredox C–H arylations with aryl diazonium salts as aryl radical sources. This method has a broad substrate scope and represents a metal-free alternative for the synthesis of 3-heteroaryl-substituted 4*H*-quinolizin-4-ones and azino- and azolo-fused pyrimidones with a bridgehead nitrogen atom.

## 1 Introduction

**Figure 1:**
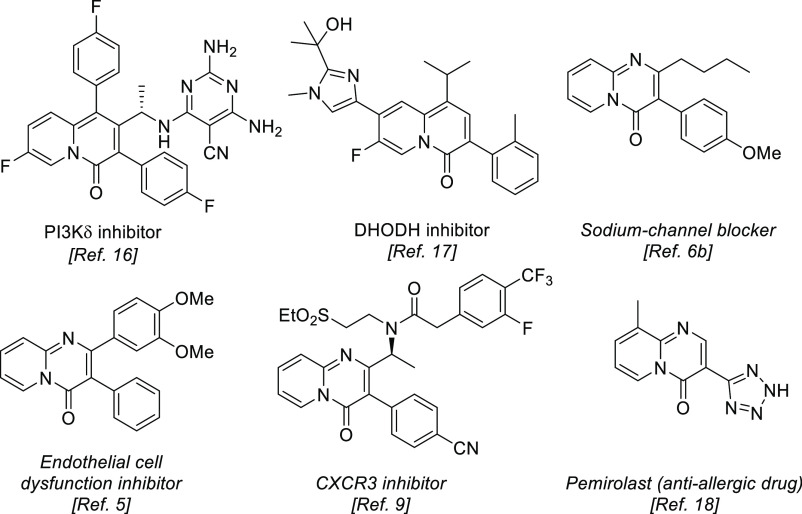
Some examples of biologically active 3-aryl-4*H*-quinolizin-4-one and 3-aryl-4*H*-pyrido[1,2-*a*]pyrimidin-4-one derivatives.

Fused heterocycles with bridgehead nitrogen atoms are important scaffolds commonly used as building blocks for applications in medicinal chemistry, bioorganic chemistry, catalysis, and materials science.^1^ In this context, quinolizines^2^ and pyrido[1,2-*a*]pyrimidines^3^ are of particular interest due to their biological activity. The significance of these fused systems is supported by the literature data,^4^ which show that 4-oxo-3-phenyl-4*H*-pyrido[1,2-*a*]pyrimidine is a substructure of more than 850 known biologically active compounds. They are associated with 21 different bioindicators, such as cardiovascular- (416),^5,6^ antitumor- (374),^7,8^ nervous system- (321),^9,10^ anti-inflammatory (306),^11,12^ and anti-infective agents (284).^13,14^ As many of these heterocyclic systems are fluorescent, they are also suitable for fluorescent sensing and labeling.^15^ Some examples of biologically active 3-aryl-4*H*-quinolizine-4-one^16,17^ and 3-aryl-4*H*-pyrido[1,2-*a*]pyrimidin-4-one derivatives, including the anti-allergic drug pemirolast^18^ are depicted in Figure 1.

**Figure 2:**
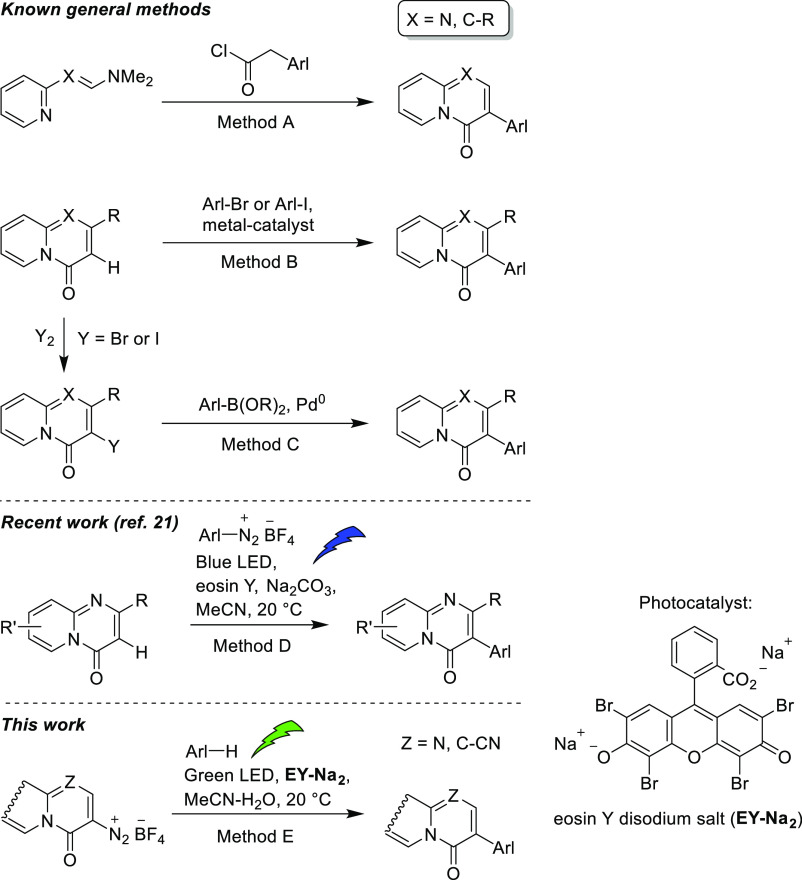
Synthesis of 3-(hetero)aryl-substituted 4-oxo-4*H*-quinolizines and 4-oxo-4*H*-pyrido[1,2-*a*]pyrimidines.

Known methods for the synthesis of 3-heteroaryl-substituted 4-oxo-4*H*-quinolizine and 4-oxo-4*H*-pyrido[1,2-*a*]pyrimidine derivatives (Figure 2) comprise cyclative condensation of 2-enamino- or 2-formamidino-substituted pyridine derivatives with α-[(hetero)aryl]acetyl chlorides (Figure 2, method A),^2,3,6^ transition metal-catalyzed direct C–H arylation of 3-unsubstituted quinolizinones and pyrido[1,2-*a*]pyrimidones with haloarenes (Figure 2, method B),^2,3,7,19^ and halogenation of the same starting compounds at position 3, followed by Suzuki–Miyaura arylation (Figure 2, method C).^2,3,20^ Despite their simplicity, the drawbacks of these synthetic approaches are the limited availability of α-(heteroaryl)acetyl chlorides (Figure 2, method A) and the use of transition-metal catalysts in the final synthetic step (Figure 2, methods B and C). Therefore, more sustainable and environmentally friendly methods that use readily available reagents and avoid the use of transition-metal catalysts are highly welcome. Recently, Kshirsagar and Bhawale reported visible-light-assisted direct C–H arylation of pyrido[1,2-*a*]pyrimidin-4-ones and thiazolo[3,2-*a*]pyrimidin-5-ones with aryl diazonium tetrafluoroborates under mild reaction conditions (Figure 2, method D).^21^ A complementary way to access title compounds is organocatalyzed photoredox-catalyzed arylation of 4-oxo-4*H*-quinolizine-3- and 4-oxo-4*H*-pyrido[1,2-*a*]pyrimidine-3-diazonium salts, accessible in three steps from alkyl 2-acylamino-3-(dimethylamino)propenoates (Figure 2, method E).^22^

Aryldiazonium salts are useful and versatile reagents with widespread applications in organic synthesis including arylation reactions.^23^ In organic photoredox catalysis,^24^ aryldiazonium salts are widely used for the arylation of arenes, alkenes, and alkynes.^25^ However, the use of aryldiazonium salts in known photoredox transformations is mostly limited to derivatives of benzenediazonium salts, while the use of their bicyclic and heterocyclic analogues remains poorly represented.^24,25^

In this context, we reported the synthesis of quinolizinone-diazonium tetrafluoroborates^26^ and azino-^27,28^ and azolo-fused pyrimidone-diazonium tetrafluoroborates.^29^ Under thermal conditions, these diazonium salts underwent various ring-transformation reactions to afford 1*H*-1,2,3-triazole,^27−29^ indolizine,^26,30^ pyrazole,^31^ and pyridine derivatives.^30,32^ Substitution of the diazonium group by the azido group^30^ and reduction to 3-unsubstituted analogues with isopropanol^26,28,29^ have also been carried out. In extension, we reasoned that these heteroaryldiazonium tetrafluoroborates could also serve as a source of heteroaryl radical intermediates. Accordingly, they could be used as valuable precursors in photocatalytic C–H arylation reactions to obtain 3-heteroaryl-substituted quinolizinones and fused pyrimidinones in an environmentally friendly and sustainable manner (cf. Figure 2, method E). Consequently, we performed a study on the arylation of title diazonium salts with a series of heteroarenes under photocatalytic conditions. Herein, we present the results of the study, which confirmed the applicability of title diazonium tetrafluoroborates in catalytic photoredox C–H arylation reactions.

## 2 Results and Discussion

### 2.1 Synthesis and Arylation of Diazonium Salts

**Scheme 1:**
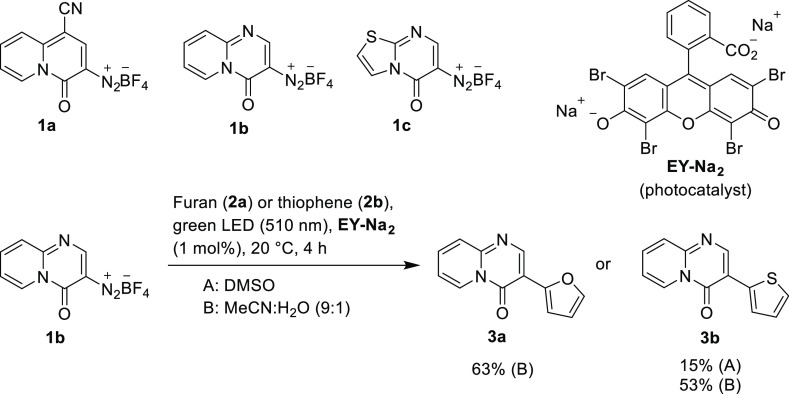
Title Diazonium Tetrafluoroborates **1a–c** and the Preliminary Visible-Light-Mediated C–H Arylation of **1b** with Thiophene (**2b**) in DMSO and with Furan (**2a**) and Thiophene (**2b**) in Aqueous Acetonitrile

Title diazonium salts, 1-cyano-4-oxo-4*H*-quinolizine-3-diazonium tetrafluoroborate (**1a**),^26^ 4-oxo-4*H*-pyrido[1,2-*a*]pyrimidine-3-diazonium tetrafluoroborate (**1b**),^27^ and 5-oxo-5*H*-thiazolo[3,2-*a*]pyrimidin-6-diazonium tetrafluoroborate (**1c**)^29^ were selected as model substrates for photocatalytic arylation reactions. Compounds **1a–c** were prepared in three steps from methyl 2-benzyloxycarbonylamino-3-(dimethylamino)propenoate and (2-pyridyl)acetonitrile, 2-aminopyridine, and 2-aminothiazole, respectively, following the literature procedures.^26,27,29^ Inspired by closely related C–H arylation of heteroarenes with aryl diazonium salts reported by König and co-workers,^33^ we have chosen their optimized reaction conditions as the starting point for our study. Eosin Y disodium salt (**EY-Na**_**2**_) was suitable as a photocatalyst as the absorption maxima of diazonium salts **1a–c** (below 425 nm) did not interfere with the absorption maximum of **EY-Na**_**2**_ at 520 nm (Figures S9–S12).^34^ Irradiation of a mixture of diazonium salt **1b** (1 equiv), thiophene (**2b**, 10 equiv), and **EY-Na**_**2**_ (1 mol %) with green light (λ = 510 nm) in degassed anhydrous DMSO under nitrogen at 20 °C for 4 h, followed by extraction workup gave the arylation product **3b** as a 4:1 mixture of two regioisomers in a 15% isolated yield (Scheme 1, conditions A). Replacing DMSO^33^ with aqueous acetonitrile (acetonitrile/water, 9:1),^23−25,35^ increased the yield to 53% as an 81:19 mixture of two isomers. In this case, extraction workup was not necessary and the product was isolated by flash chromatography (FC) only. Neutral alumina proved to be an optimal stationary phase because the catalyst and polar components remained absorbed, allowing for easy isolation of the pure, less polar product **3b**. The reaction of **1b** with excess furan (**2a**) under these conditions afforded the arylation product **3a** as a single isomer in a 63% isolated yield (Scheme 1, conditions B).

Based on preliminary results, we decided to use the reaction of diazonium tetrafluoroborate **1b** with furan (**2a**) as a model reaction for the subsequent optimization study. The results of the optimization study are shown in Table 1. Irradiation of a mixture of diazonium salt **1b** (1 equiv), furan (**2a**) (10 equiv), and **EY-Na**_**2**_ (1 mol %) with green light (λ = 510 nm) in a 9:1 mixture of acetonitrile and water at 20 °C for 4 h gave the arylation product **3a** in a 77% NMR yield (Table 1, entry 1). The yields of **3a** were lower when the content of water was either gradually increased to a 1:1 ratio (Table 1, entries 2 and 3) or gradually decreased to 19:1 (Table 1, entries 4 and 5). No product **3a** was observed in anhydrous acetonitrile and other organic solvents, such as ethyl acetate, methanol, THF, and toluene (Table 1, entry 6). This is consistent with the insolubility of the diazonium salts in anhydrous organic solvents. Higher catalyst loading did not improve the yield (Table 1, entries 7 and 8) while decreasing the amount of catalyst to 0.5 mol % resulted in a slight decrease in yield to 64% (Table 1, entry 9). Shortening the wavelength from 510 nm (green light) to 450 nm (blue light) had a negligible effect on the yield (69%, Table 1, entry 10). Without light and in the presence of **EY-Na**_**2**_, the conversion was slowed down to give **3a** in a 41% yield after 20 h (Table 1, entry 11), while without light and photocatalyst, the yield was 31% after 24 h (Table 1, entry 12). When the reaction under standard conditions (MeCN/H_2_O = 9:1, λ = 510 nm, 1 mol % **EY-Na**_**2**_) was performed in a non-degassed solvent, it showed a slight decrease in NMR yield to 68% after 4 h (Table 1, entry 13). In contrast, without light and catalyst, the NMR yield in non-degassed solvent was only 3% after 4 h (Table 1, entry 14), while lowering the reaction temperature to 4 °C stopped the reaction (Table 1, entry 15). Replacement of **EY-Na**_**2**_ with [Ru(bpy)_3_](PF_6_)_2_, rose bengal disodium salt (RB-Na_2_), rhodamine B, and 9-mesityl-10-methylacridinium perchlorate (Mes-Acr-MeClO_4_) as photocatalysts resulted in a decrease of the yield (Table 1, entries 16–19). These results clearly indicated that the initial reaction conditions (MeCN/H_2_O = 9:1, λ = 510 nm, 1 mol % **EY-Na**_**2**_, degassed solvent, nitrogen atmosphere) were optimal (Table 1, entry 1).

**Table 1:**
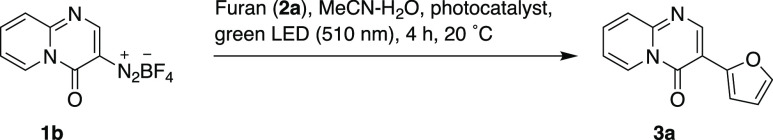
Optimization of the Reaction Conditions Using **1b** + **2a** → **3a** as Model Transformation^a^

entry	MeCN/H_2_O	time (h)	*T* (°C)	λ (nm)	photocatalyst (mol %)	yield (%)^b^
1	9:1	4	20	510	**EY-Na**_**2**_ (1)	77
2	7:1	4	20	510	**EY-Na**_**2**_ (1)	55
3	1:1	4	20	510	**EY-Na**_**2**_ (1)	50
4	12:1	4	20	510	**EY-Na**_**2**_ (1)	25
5	19:1	4	20	510	**EY-Na**_**2**_ (1)	21
6	100:0^c^	4	20	510	**EY-Na**_**2**_ (1)	0^c^
7	9:1	4	20	510	**EY-Na**_**2**_ (2.5)	70
8	9:1	4	20	510	**EY-Na**_**2**_ (5)	60
9	9:1	4	20	510	**EY-Na**_**2**_ (0.5)	64
10	9:1	4	20	450	**EY-Na**_**2**_ (1)	69
11	9:1	20	20	d	**EY-Na**_**2**_ (1)	41
12	9:1	24	20	d	no catalyst	31
13	9:1	4	20	510	**EY-Na**_**2**_ (1)	68^e^
14	9:1	4	20	d	no catalyst	3^e^
15	9:1	20	4	d	no catalyst	1^e^
16	9:1	4	20	450	[Ru(bpy)_3_](PF_6_)_2_ (1)	49
17	9:1	4	20	510	RB-Na_2_ (1)^f^	45
18	9:1	4	20	510	rhodamine B (1)	49
19	9:1	4	20	450	Mes-Acr-MeClO_4_ (1)^g^	66

a Unless otherwise stated, the following standard reaction conditions were used: diazonium salt **1b** (0.2 mmol), furan **2a** (2 mmol), MeCN/H_2_O (9:1, 2 mL), **EY-Na**_**2**_ (1 mol %), λ = 510 nm, *T* = 20 °C, and *t* = 4 h.

b NMR yield; determined using 1,3,5-trimethoxybenzene as an internal standard.

c No product **3a** formation was observed also in MeOH, THF, EtOAc, and toluene.

d No light.

e In non-degassed solvent under an air atmosphere.

f Rose bengal disodium salt.

g 9-Mesityl-10-methylacridinium perchlorate.

**Scheme 2:**
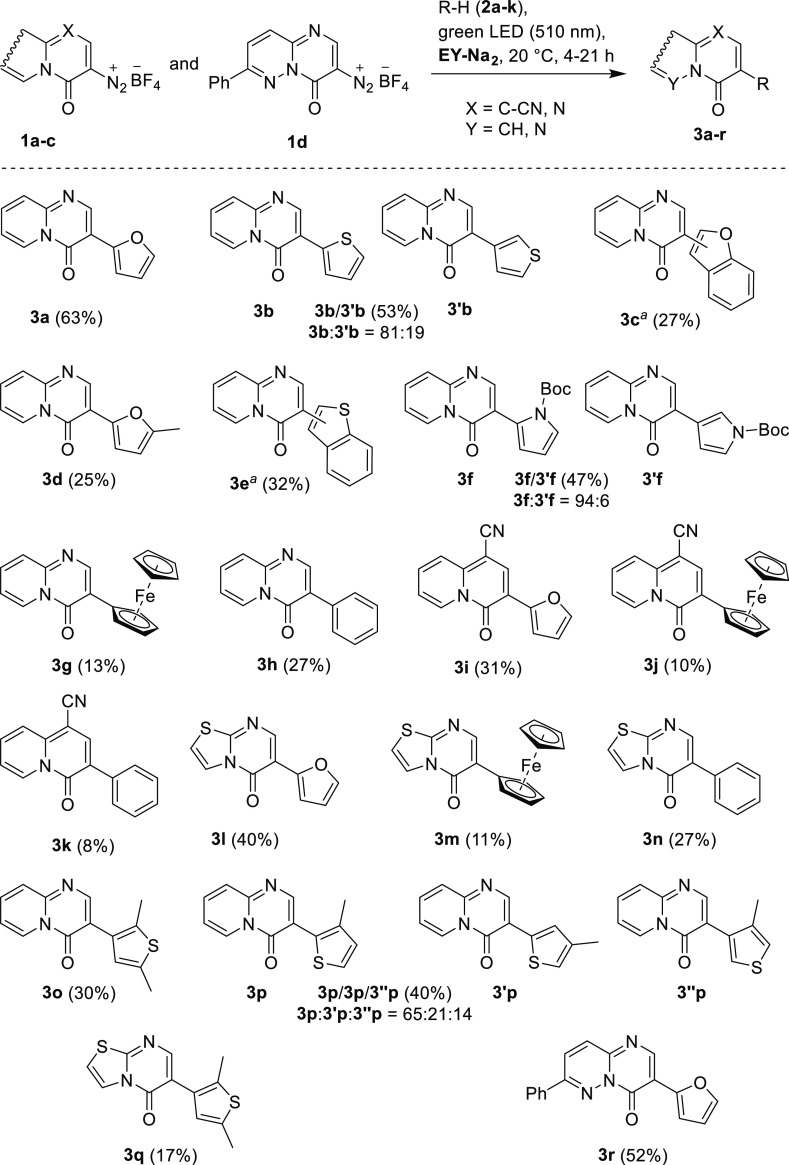
C–H Arylations of Diazonium Salts **1a–c** with Heteroarenes **2a–f**, **i**, **j**, Ferrocene (**2g**), and Benzene (**2h**), Reaction conditions: diazonium salt **1b** (0.5 mmol), heteroarene **1a–h** (0.5–5.0 mmol), MeCN/H_2_O (9:1, 2 mL), **EY-Na**_**2**_ (1 mol %), λ = 510 nm, *T* = 20 °C, and *t* = 4–21 h. ^*b*^ A mixture of several regioisomers. A mixture of several regioisomers.

With the optimized reaction conditions in hand, the substrate scope was examined. The results are shown in Scheme 2. First, diazonium tetrafluoroborate **1b** was treated with heteroarenes **2a–f**, ferrocene (**2g**), and benzene (**2h**) under optimized reaction conditions to give the corresponding arylation products **3a–h** in 13–63% isolated yields (Scheme 2). Reactions of **1b** with furan (**2a**), 2-methylfuran (**2d**), ferrocene (**2g**), and benzene (**2h**) gave the corresponding 3-(hetero)aryl-4-oxo-4*H*-pyrido[1,2-*a*]pyrimidines **3a**, **3d**, **3g**, and **3h** as single products in 13–63% isolated yields. The reaction of **1b** with thiophene (**2b**) gave the arylation product **3b/3′b** as an 81:19 mixture of the major 2-thienyl- (**3b**) and the minor 3-thienyl-isomer **3′b** in 53% isolated yield. Similarly, treatment of **1b** with 1-*tert*-butoxycarbonyl-1*H*-pyrrole (**2f**) gave the arylation product **3f/3′f** as a 93:7 mixture of the respective pyrrol-2-yl- (**3f**) and pyrrol-3-yl-substituted isomer **3′f** in 47% isolated yield. Reactions of **1b** with benzofuran (**2c**) and benzothiophene (**2e**) gave the corresponding arylation products **3c** and **3e** as mixtures of at least five regioisomers. The substrate scope was further explored in the reactions of 1-cyano-4-oxo-4*H*-quinolizine-3-diazonium tetrafluoroborate (**1a**) and 5-oxo-5*H*-thiazolo[3,2-*a*]pyrimidine-6-diazonium tetrafluoroborate (**1c**) with furan (**2a**), ferrocene (**2g**), and benzene (**2h**). As expected, all six transformations afforded the corresponding arylation products **3i–n** as single isomers in 8–40% isolated yields. Next, arylations of thiophene derivatives **2i** and **2j** with diazonium salts **1b** and **1c** were investigated. The reaction of **1b** with 2,5-dimethylthiophene (**2i**) gave single arylation product **3o** in a 30% yield, while arylation of **1b** with 3-methylthiophene (**2j**) afforded a mixture of three isomeric products **3p**, **3′p**, and **3″p** in a ratio of 65:21:14, respectively. Somewhat expectedly, the reaction of 5-oxo-5*H*-thiazolo[3,2-*a*]pyrimidine-6-diazonium tetrafluoroborate (**1c**) with 2,5-dimethylthiophene (**2i**) gave a single arylation product of **3q** in a 17% yield. Finally, the reaction scope was expanded with another diazonium salt, 4-oxo-7-phenyl-4*H*-pyrimido[1,2-*b*]pyridazine-3-diazonium tetrafluoroborate (**1d**).^28^ Treatment of **1d** with excess furan (**2a**) under standard conditions gave the expected product **3r** in a 52% yield. It is also worth mentioning that attempted arylations of **1b** with 1*H*-indole, 1-methyl-1*H*-indole, and 1-methyl-1*H*-pyrrole produced black semi-solid polymeric products and not the desired arylated compounds (Scheme 2).

The moderate isolated yields of compounds **3** are consistent with yields reported for photoredox transformations of aryl diazonium salts based on bicyclic and heterocyclic systems. In general, these yields are significantly lower than those obtained in the same reactions performed with analogous benzenediazonium salts.^24,25,35,36^ Formation of the major 2-furyl, 2-thienyl, and 2-pyrrolyl isomers **3a**, **3b**, **3d**, **3f**, **3p**, and **3r** was in agreement with the regioselectivity in closely related arylation reactions, while loss of selectivity in reactions with benzofuran (**2c**) and benzothiophene (**2e**) was somewhat surprising.^25^ The regioselective arylation of furan and thiophene derivatives at C(2) is explainable by a large HOMO coefficient of C(2),^37^ which directs the attack of a heteroaryl radical to this position.^38^

### 2.2 Structure Determination

The structures of novel compounds **3a–g**, **3i**, **3j**, **3l**, **3m**, and **3p**–**r** were determined by spectroscopic methods [IR, NMR spectroscopy (^1^H- and ^13^C-NMR, COSY, HSQC, HMBC, and NOESY spectroscopy), and MS-HRMS] and by elemental analyses for C, H, and N. Physical and spectral data of known arylation products **3h**,^6,19,20^**3k**,^39^ and **3n**(40) were in agreement with the literature data. Ratios of isomers in arylation products **3b/3′b** and **3f/3′f** were determined from the relative intensities of well-resolved characteristic signals in their ^1^H-NMR spectra.^34^ The structures of **3b** and **3′b** and assignments of signals for protons and carbon nuclei of each isomer in their ^1^H- and ^13^C-NMR spectra were determined by COSY, HSQC, HMBC, and NOESY spectroscopy (see the Supporting Information for details).^34^ The structure of compound **3n** was also determined by X-ray diffraction (Figure S21).^34^ The arylation products **3a–f**, **3h**, **3i**, **3k**, **3l**, and **3n–r** also showed fluorescence upon excitation with UV light, both in solution and in the solid state (Figures S14 and S15), whereas the ferrocenyl-substituted compounds **3g**, **3j**, and **3m** were not fluorescent.^34^

### 2.3 Elucidation of the Reaction Mechanism

**Figure 3:**
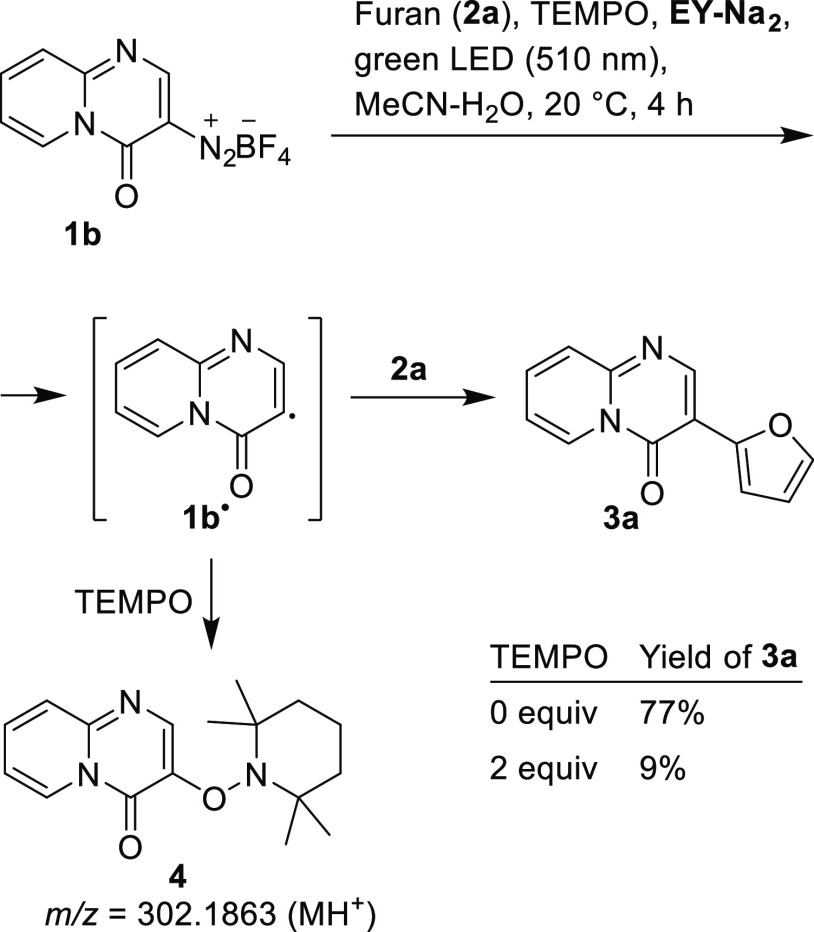
Effect of TEMPO on NMR yields of **3a** in the model reaction (**1b** + **2a** → **3a**).

Several recent studies have shown that photocatalytic C–H arylations of aryldiazonium salts proceed via a radical mechanism.^25,33,38,41^ The initial reactive species is an aryl radical intermediate formed by SET from the excited state of the catalyst to the aryldiazonium cation to give the corresponding nitrogen-centered radical, followed by the elimination of nitrogen.^25,33,38,41^ The reaction conditions optimization study (cf. Table 1) already showed that the conversion was lower in the absence of light or/and catalyst (cf. Table 1, entries 11–15), which was in agreement with the postulated radical mechanism. To confirm the radical mechanism, the model reaction **1b** + **2a** → **3a** was performed in the presence of TEMPO as a radical scavenger. As shown in Figure 3, the NMR yield of the arylation product **3a** decreased significantly from 77% without TEMPO to 9% with TEMPO. The addition of TEMPO also decreased the yield of **3a** when the reaction was carried out under air in a non-degassed solvent (Table S4).^34^ These results were consistent with the formation of the heteroaryl radical **1b**^•^ from the respective diazonium salt **1b** (Figure 3).

**Figure 4:**
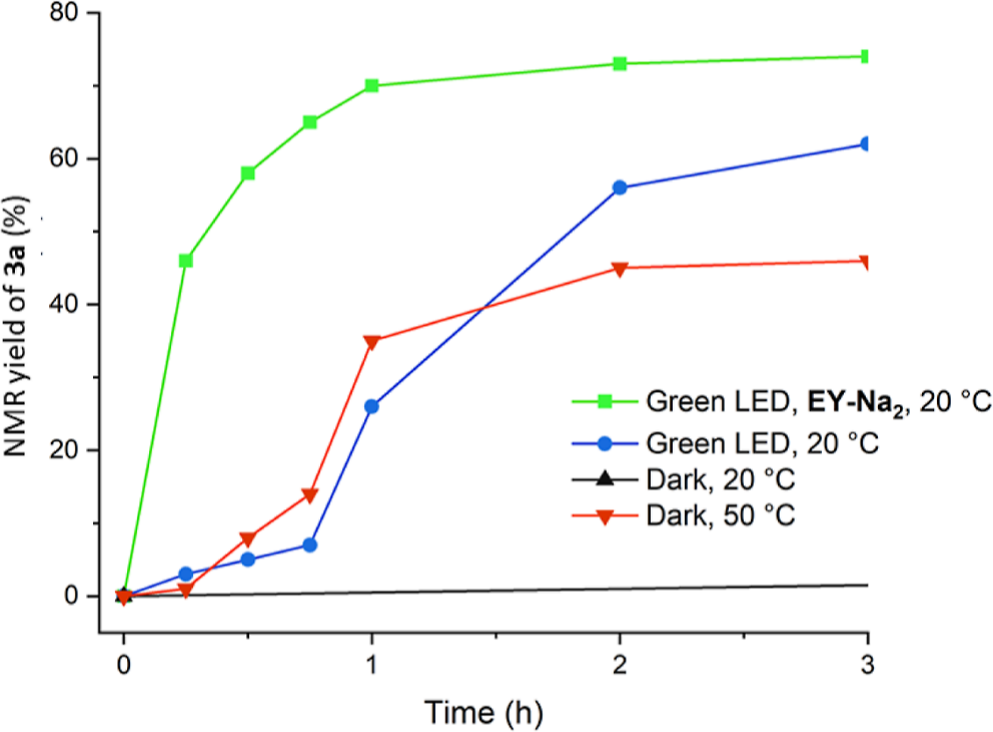
Zoomed kinetic profiles of model transformation **1b** + **2a** → **3a** within the first 3 h under photochemical conditions in the presence (**—**) or absence (**—**) of **EY-Na**_**2**_ and under thermal conditions in the absence of **EY-Na**_**2**_ at 20 °C (**—**) and 50 °C (**—**).

Next, the kinetic profiles for the model transformation (**1b** + **2a** → **3a**) were determined by ^1^H NMR under standard conditions (MeCN/H_2_O = 9:1, 510 nm, 1 mol % of **EY-Na**_**2**_, 20 °C, 48 h) and slightly varied reaction conditions. The results are shown in Figure 4 and Figure S20.^34^ The fastest reaction rate and the highest conversion were determined under standard photocatalytic conditions, which gave an NMR yield of 74% for **3a** after 3 h (Figure 4, green line), whereas the reaction rate and yield decreased in the absence of **EY-Na**_**2**_ (62% after 3 h, Figure 4, blue line). Although the thermal reaction at 20 °C was initially very slow, the reaction rate gradually increased, reaching 55% NMR yield after 48 h (Figure S20, black line). The thermal reaction proceeded faster at 50 °C, giving **3a** in 46% yield after 3 h (Figure 4, red line).^34^ Notably, only the photocatalytic reaction showed a logarithmic kinetic profile (Figure 4, green line), while sigmoidal kinetic profiles were obtained for the other three reactions (Figure S20, black line and Figure 4, blue and red line). The logarithmic kinetic profile of the photocatalytic transformation in the presence of **EY-Na**_**2**_ (Figure 4, green line) is consistent with the photocatalytic radical reaction mechanism. The sigmoidal kinetic profiles of the reactions in the absence of **EY-Na**_**2**_ suggest an autocatalytic or radical-chain reaction mechanism. Since **3a** does not absorb light at wavelengths above 450 nm (Figure S13),^34^ autocatalysis by product **3a** was ruled out. On the other hand, the sigmoidal kinetic profiles are consistent with the radical-chain reaction mechanism.^38,41^ This is possible because aryl radicals are generated from the respective aryl diazonium salts by the action of mild bases even under non-reducing thermal conditions,^42^ for example, by the use of pyridine or tertiary amines.^43^ In the model reaction (**1b** + **2a** → **3a**), the ring nitrogen atoms in diazonium salts **1** and in product **3** can act as weak tertiary amine bases (Figure S20, black line and Figure 4, red line).^34^

**Scheme 3:**
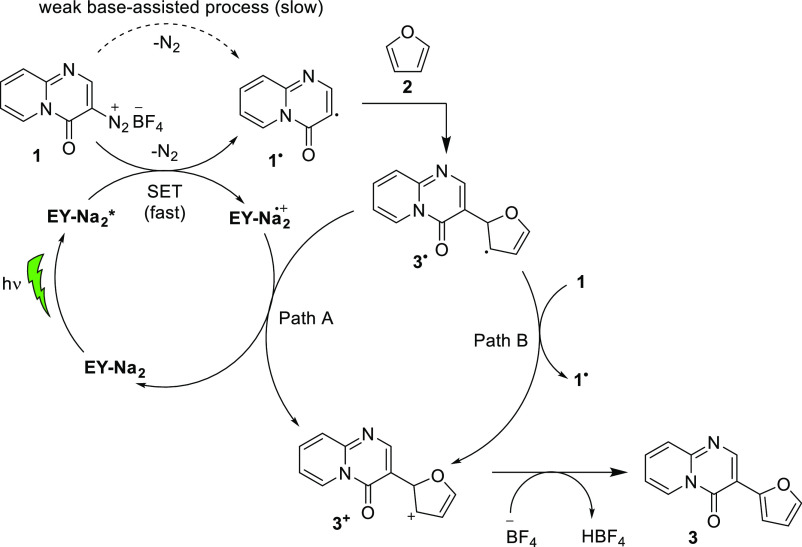
Plausible Reaction Mechanism Consisting of Photocatalytic C–H Arylation of Diazonium Salts **1** with Heteroarenes **2** Catalyzed by **EY-Na**_**2**_ and Product **3** (Path A) and Radical-Chain C–H Arylation of Diazonium Salts **1** with Heteroarenes **2** (Path B)

The above results support the radical mechanism of photocatalytic C–H arylation and are in line with the literature data for related photocatalytic C–H arylations.^25^ On the basis of our results and related literature examples,^25,33,38,41–43^ a plausible mechanism for photocatalytic arylation of title heteroaryl diazonium salts **1** with (hetero)arenes **2** is proposed (Scheme 3). The general structures of diazonium salts **1**, (hetero)arenes **2**, and intermediates **1**^•^, **3**^•^, and **3**^**+**^ are presented using 4-oxo-4*H*-pyrido[1,2-*a*]pyrimidine and furan as representative scaffolds. The reaction starts with light-promoted SET from **EY-Na**_2_* to heteroaryl diazonium salt **1**, followed by homolytic cleavage of the C–N bond and elimination of dinitrogen gas to generate the heteroaryl radical **1**^•^. To a smaller extent, heteroaryl radical **1**^•^ is also generated thermally with the assistance of another molecule of **1** or **3**, which act as weak bases.^42,43^ The addition of **1**^•^ to heteroarene **2** generates the radical intermediate **3**^•^, which is then oxidized with **EY-Na**_**2**_ radical cation (**EY-Na**_**2**_^•**+**^, path A) and/or with diazonium salt **1** (path B) into the corresponding carbocation intermediate **3**^**+**^. Deprotonation of carbocation **3**^**+**^ then gives the final product **3**. Notably, path A is a part of a photocatalytic cycle, whereas path B represents the competitive radical-chain reaction process (Scheme 3).

## 3 Conclusions

A series of novel 3-(hetero)aryl-substituted 4-oxo-4*H*-quinolizine and 4-oxo-4*H*-pyrido[1,2-*a*]pyrimidine derivatives **3a–n** were prepared by visible light-promoted photoredox arylation of the corresponding 4-oxo-4*H*-quinolizine-3- (**1a**), 4-oxo-4*H*-pyrido[1,2-*a*]pyrimidine-3- (**1b**), 5-oxo-5*H*-thiazolo[3,2-*a*]pyrimidine-6-diazonium tetrafluoroborate (**1c**), and 4-oxo-7-phenyl-4*H*-pyrimido[1,2-*b*]pyridazine-3-diazonium tetrafluoroborate (**1d**) with heteroarenes **2a–f**, **i–k**, ferrocene (**2g**), and benzene (**2h**). Title reactions are the rare examples of the use of heteroaryldiazonium salts in photoredox chemistry in general, while the photocatalytic C–H heteroarylation using quinolizinone-, azaquinolizinone-, or azolo-fused 4-pyrimidinone-diazonium salts is, to our knowledge, unprecedented. This synthetic method has a broad substrate scope, takes place at room temperature, and does not require a transition-metal catalyst. Although typically isolated yields have been moderate (up to 63%), the yields obtained with furan, pyrrole, and thiophene derivatives are comparable to those obtained in related C–H arylations with aryldiazonium salts (around 60%).^25,33,36^ On the other hand, C–H arylations have also been successful with ferrocene and led to the first examples of ferrocenyl-substituted quinolizinone- and azaquinolizinone-derivatives. Therefore, photocatalytic C–H arylation of title diazonium salts is a simpler and milder alternative to known methods and could become a useful synthetic tool for the preparation of 3-(hetero)aryl-substituted azino- and azolo-fused 4-pyridones and 4-pyrimidones with bridgehead nitrogen atoms - a class of compounds of particular interest due to their biological activities and optical properties.

## 4 Experimental Section

### 4.1 General Methods

Photocatalytic transformations were carried out on a custom-made photoreactor using LED illumination, magnetic stirring, and a cooling block to sustain a reaction temperature of 20 °C (see the Supporting Information for details).^34^ Melting points were determined on a Kofler micro hot stage and on an automated melting point system. The NMR spectra were recorded in CDCl_3_ and DMSO-*d*_6_ using TMS as the internal standard on a 500 or 600 MHz instrument at 500 and 600 MHz for ^1^H and at 125 and 150 MHz for the ^13^C nucleus, respectively. Mass spectra were recorded on a TOF LC/MS spectrometer and IR spectra on a FTIR ATR spectrophotometer. Microanalyses were performed by combustion analysis on a CHN analyzer. Column chromatography (CC) and FC were performed on neutral alumina (particle size: 0.035–0.070 mm).

Heteroarenes **2a–f**, ferrocene (**2g**), benzene (**2h**), and **EY-Na**_**2**_ are commercially available. 1-Cyano-4-oxo-4*H*-quinolizine-3-diazonium tetrafluoroborate (**1a**),^26^ 4-oxo-4*H*-pyrido[1,2-*a*]pyrimidine-3-diazonium tetrafluoroborate (**1b**),^27^ 5-oxo-5*H*-thiazolo[3,2-*a*]pyrimidine-6-diazonium tetrafluoroborate (**1c**),^29^ and 4-oxo-7-phenyl-4*H*-pyrimido[1,2-*b*]pyridazine-3-diazonium tetrafluoroborate (**1d**)^28^ were prepared following the literature procedures.

### 4.2 General Procedure for the Photocatalytic Arylation of Heteroaryl Diazonium Tetrafluoroborates **1a–c**

#### 4.2.1 Synthesis of Heteroaryl-Substituted Quinolizinones, Pyrido[1,2-*a*]pyrimidinones, and Thiazolo[3,2-*a*]pyrimidinones **3a–n**

A 5 mL vial with a screw cap and septum was charged with diazonium tetrafluoroborate **1** (0.5 mmol), heteroarene **2** (0.5–5 mmol), **EY-Na**_**2**_ (3.5 mg, 5 μmol), acetonitrile (1.8 mL), and water (0.2 mL). The vial was stopped, carefully degassed, and purged with nitrogen using the freeze–pump–thaw technique. Then, the vial was mounted into a photoreactor and irradiated with green light (510 nm) at 20 °C for 4–21 h. The reaction mixture was purified by CC or FC (neutral alumina, ethyl acetate/petroleum ether). Fractions containing the product were combined and evaporated in vacuo to give the arylation product **3**.

##### 4.2.1.1 3-(Furan-2-yl)-4*H*-pyrido[1,2-*a*]pyrimidin-4-one (**3a**)

From diazonium salt **1b** (130 mg, 0.5 mmol) and furan (**2a**) (365 μL, 5 mmol), 4 h, CC (ethyl acetate/petroleum ether = 1:1). Yield: 67 mg (63%) of yellow solid; mp 204–206 °C. ^1^H NMR (500 MHz, CDCl_3_): δ 9.18 (dt, *J* = 7.2, 1.2 Hz, 1H), 8.92 (s, 1H), 7.81–7.65 (m, 2H), 7.51 (dd, *J* = 1.8, 0.8 Hz, 1H), 7.31 (dd, *J* = 3.4, 0.8 Hz, 1H), 7.20 (ddd, *J* = 7.2, 5.6, 2.5 Hz, 1H), 6.56 (dd, *J* = 3.4, 1.8 Hz, 1H). ^13^C{^1^H} NMR (126 MHz, CDCl_3_): δ 154.2, 149.5, 149.0, 147.9, 141.9, 135.1, 127.4, 126.8, 116.1, 112.1, 110.5, 108.9. *m*/*z* (HRMS); found, 213.0654 (MH^+^). C_12_H_9_N_2_O_2_ requires *m*/*z* = 213.0659. Anal. Calcd for C_12_H_8_N_2_O_2_: C, 67.92; H, 3.80; N, 13.20. Found: C, 67.70; H, 3.42; N, 13.00. ν_max_ (ATR): 3110, 2916, 1687 (C=O), 1632 (C=O), 1476, 1334, 1255, 1087, 1005, 814, 744 cm^–1^.

##### 4.2.1.2 3-(Thiophen-2-yl)-4*H*-pyrido[1,2-*a*]pyrimidin-4-one (**3b**) and 3-(Thiophen-3-yl)-4*H*-pyrido[1,2-*a*]pyrimidin-4-one (**3′b**)

From diazonium salt **1b** (130 mg, 0.5 mmol) and thiophene (**2b**) (200 μL, 2.5 mmol), 19 h, CC (ethyl acetate). Yield: 60 mg (53%) of yellow solid; mp 195–197 °C; **3b/3′b** = 81:19. ^1^H NMR (500 MHz, CDCl_3_): δ major isomer **3b**: 9.21 (dt, *J* = 7.2, 1.2 Hz, 6 H), 8.87 (s, 2 H), 7.76–7.67 (m, 8 H, 9 H, 3′ H), 7.41 (dd, *J* = 5.2, 1.1 Hz, 5′ H), 7.22 (ddd, *J* = 7.1, 6.1, 1.9 Hz, 7 H), 7.15 (dd, *J* = 5.1, 3.7 Hz, 4′ H); minor isomer **3′b**: 9.21 (dt, *J* = 7.2, 1.2 Hz, 6 H), 8.75 (s, 2 H), 8.23 (dd, *J* = 3.0, 1.3 Hz, 2′ H), 7.76–7.67 (m, 8 H, 9 H), 7.63 (dd, *J* = 5.1, 1.3 Hz, 4′ H), 7.41 (dd, *J* = 5.2, 3.1 Hz, 5′ H), 7.19 (ddd, *J* = 7.2, 6.1, 1.6 Hz, 7 H). ^13^C{^1^H} NMR (126 MHz, CDCl_3_): δ major isomer **3b**: 155.4, 150.1, 149.6, 135.3, 135.3, 127.7, 127.1, 126.7, 126.2, 124.1, 116.3, 112.0; minor isomer **3′b**: 156.2, 151.5, 149.9, 135.7, 134.1, 127.6, 126.6, 125.9, 125.5, 123.9, 115.9, 112.5. ^1^H NMR (500 MHz, DMSO-*d*_6_): δ major isomer **3b**: 9.12 (dq, *J* = 7.1, 0.8 Hz, 6-H), 9.05 (s, 2-H), 7.97 (dd, *J* = 8.6, 6.7, 1.5 Hz, 8 H), 7.86 (dd, *J* = 3.7, 1.1 Hz, 3′ H), 7.79 (dt, *J* = 8.7, 1.1 Hz, 9 H), 7.57 (dd, *J* = 5.1, 1.0 Hz, 5′ H), 7.46 (td, *J* = 7.0, 1.4 Hz, 7 H), 7.16 (dd, *J* = 5.1, 3.7 Hz, 4′ H); minor isomer **3′b**: 9.13 (dq, *J* = 7.1, 0.8 Hz, 6 H), 8.94 (s, 2 H), 8.33 (dd, *J* = 3.0, 1.2 Hz, 2′ H), 7.96 (ddd, *J* = 8.6, 6.7, 1.5 Hz, 8 H), 7.84 (dd, *J* = 5.1, 1.2 Hz, 4′ H), 7.75 (dt, *J* = 8.6, 1.0 Hz, 9 H), 7.64 (dd, *J* = 5.1, 3.0 Hz, 5′ H), 7.42 (td, *J* = 7.0, 1.3 Hz, 7 H). ^13^C{^1^H} NMR (126 MHz, DMSO-*d*_6_): δ major isomer **3b**: 154.6, 150.0, 149.3, 136.8, 135.5, 127.5, 126.9, 126.5, 126.2, 123.5, 117.3, 110.4; minor isomer **3′b**: 155.4, 151.7, 149.6, 136.7, 134.5, 127.4, 126.3, 126.2, 125.8, 123.1, 116.9, 110.9. *m*/*z* (HRMS); found, 229.0428 (MH^+^). C_12_H_9_N_2_OS requires *m*/*z* = 229.0430. ν_max_ (ATR): 3080, 2922, 1655 (C=O), 1628 (C=O), 1478, 1368, 1123, 1075, 889, 800, 759, 731 cm^–1^.

##### 4.2.1.3 3-Benzo[*b*]furanyl-4*H*-pyrido[1,2-*a*]pyrimidin-4-one (a Mixture of Isomers) (**3c**)

From diazonium salt **1b** (130 mg, 0.5 mmol) and benzofuran (**2c**) (83 μL, 0.75 mmol), 19 h, CC (ethyl acetate). Yield: 35 mg (27%) of yellow-green solid; mp 130–205 °C; ratio of isomers = 37:25:18:12:8. ^1^H NMR (500 MHz, CDCl_3_): δ 9.27–9.22 (m, 1H), 9.14, 8.87, 8.62, 8.60, and 8.59 (5s, 8:25:18:12:37, 1H), 8.06–8.03, 7.83–7.19, and 6.86–6.79 (3m, 8:89:3, 8H). ^13^C{^1^H} NMR (126 MHz, CDCl_3_): δ 157.1, 156.9, 156.5, 156.4, 155.2, 155.1, 154.8, 154.6, 154.5, 154.3, 154.1, 153.0, 153.0, 152.5, 150.9, 150.6, 150.5, 150.4, 150.1, 149.9, 145.7, 145.5, 145.0, 144.9, 136.0, 135.9, 135.6, 135.5, 130.6, 129.5, 128.0, 127.9, 127.8, 127.7, 127.7, 127.7, 127.6, 127.1, 126.9, 126.9, 126.6, 126.6, 125.5, 125.1, 124.6, 124.4, 123.8, 123.4, 123.0, 123.0, 121.6, 121.3, 121.1, 121.0, 118.7, 117.2, 116.4, 116.3, 116.0, 115.9, 115.8, 112.9, 111.7, 111.4, 111.2, 110.8, 108.2, 106.9, 106.7, 106.7, 106.5. *m*/*z* (HRMS); found, 263.0807 (MH^+^). C_16_H_11_N_2_O_2_ requires *m*/*z* = 263.0815. Anal. Calcd for C_16_H_10_N_2_O_2_: C, 73.27; H, 3.84; N, 10.68. Found: C, 72.93; H, 3.53; N, 10.53. ν_max_ (ATR): 3090, 3034, 1677 (C=O), 1628 (C=O), 1486, 1293, 1256, 1119, 1071, 804, 790, 740 cm^–1^.

##### 4.2.1.4 3-(5-Methylfuran-2-yl)-4*H*-pyrido[1,2-*a*]pyrimidin-4-one (**3d**)

From diazonium salt **1b** (130 mg, 0.5 mmol) and 2-methylfuran (**2d**) (450 μL, 5 mmol), 19 h, CC (ethyl acetate/petroleum ether = 1:1). Yield: 28 mg (25%) of yellow–orange solid; mp 192–194 °C; ratio of isomers = 97:3. ^1^H NMR (500 MHz, CDCl_3_): δ 9.16 (dt, *J* = 7.2, 1.2 Hz, 1H), 8.88 (s, 1H), 7.71–7.64 (m, 2H), 7.20 (br d, *J* = 3.2 Hz, 1H), 7.17 (ddd, *J* = 7.2, 5.1, 2.9 Hz, 1H), 6.15 (br dq, *J* = 3.2, 1.0 Hz, 1H), 2.41 (br d, *J* = 1.0 Hz, 3H). ^13^C{^1^H} NMR (126 MHz, CDCl_3_): δ 154.1, 152.1, 149.0, 148.2, 146.1, 134.6, 127.3, 126.7, 115.9, 111.9, 109.3, 108.3, 13.8. *m*/*z* (HRMS); found, 227.0815 (MH^+^). C_13_H_11_N_2_O_2_ requires *m*/*z* = 227.0815. ν_max_ (ATR): 3102, 2919, 1673 (C=O), 1627 (C=O), 1570, 1485, 1366, 1256, 1106, 1018, 890, 773 cm^–1^.

##### 4.2.1.5 3-(Benzo[*b*]thiophenyl)-4*H*-pyrido[1,2-*a*]pyrimidin-4-one (a Mixture of Isomers) (**3e**)

From diazonium salt **1b** (130 mg, 0.5 mmol) and benzo[*b*]thiophene (**2e**) (100 mg, 0.75 mmol), 20 h, CC (ethyl acetate/petroleum ether = 3:1). Yield: 46 mg (32%) of yellow solid; mp 93–193 °C; ratio of isomers = 21:21:20:19:11:8. ^1^H NMR (500 MHz, CDCl_3_): δ 9.26–9.07 (m, 1H), 8.89, 8.72, 8.66, 8.64, 8.63, and 8.55 (6s, 21:8:20:19:11:21, 1H), 8.42–8.12, 7.98–7.64, 7.58–7.27, 7.25–7.13, and 6.47–6.45 (5m, 2:12:31:48:7, 8H). ^13^C{^1^H} NMR (126 MHz, CDCl_3_): δ 157.0, 156.9, 156.7, 156.6, 156.1, 155.3, 154.8, 154.4, 154.1, 153.7, 153.0, 151.5, 151.2, 151.1, 150.8, 150.6, 150.6, 150.1, 140.4, 140.3, 140.2, 140.1, 140.0, 139.9, 139.5, 139.2, 139.1, 138.5, 137.9, 136.3, 136.2, 136.1, 136.0, 135.9, 135.7, 135.6, 130.4, 130.3, 129.8, 129.5, 129.1, 128.0, 127.8, 127.8, 127.8, 127.7, 127.4, 127.2, 126.9, 126.7, 126.7, 126.6, 126.6, 126.6, 126.6, 126.5, 126.5, 126.4, 126.0, 125.9, 124.8, 124.7, 124.5, 124.5, 124.4, 124.3, 124.3, 124.2, 124.2, 123.7, 123.6, 123.6, 123.5, 123.3, 122.9, 122.9, 122.5, 122.5, 122.5, 122.0, 121.9, 117.2, 116.9, 116.7, 116.5, 116.0, 116.0, 115.9, 115.9, 115.6, 112.6, 111.6, 104.8. *m*/*z* (HRMS); found, 279.0588 (MH^+^). C_16_H_11_N_2_OS requires *m*/*z* = 279.0587. Anal. Calcd for C_16_H_10_N_2_OS requires C, 69.05; H, 3.62; N, 10.07. Found: C, 68.79; H, 3.45; N, 9.99. ν_max_ (ATR): 3097, 2917, 2849, 1667 (C=O), 1628 (C=O), 1563, 1471, 1333, 1292, 1122, 1069, 891, 757, 697 cm^–1^.

##### 4.2.1.6 *tert*-Butyl 2-(4-Oxo-4*H*-pyrido[1,2-*a*]pyrimidin-3-yl)-1*H*-pyrrole-1-carboxylate (**3f**) and *tert*-Butyl 3-(4-Oxo-4*H*-pyrido[1,2-*a*]pyrimidin-3-yl)-1*H*-pyrrole-1-carboxylate (**3′f**)

From diazonium salt **1b** (130 mg, 0.5 mmol) and *N*-Boc-pyrrole (**2f**) (167 μL, 1 mmol), 4 h, CC (ethyl acetate/petroleum ether = 1:1). Yield: 74 mg (47%) of pale yellow solid; mp 193–195 °C; **3f**:**3′f** = 93:7. ^1^H NMR (500 MHz, CDCl_3_): δ major isomer **3f**: 9.11 (ddd, *J* = 7.2, 1.6, 0.9 Hz, 1H), 8.35 (s, 1H), 7.72 (ddd, *J* = 9.0, 6.5, 1.6 Hz, 1H), 7.66 (ddd, *J* = 9.0, 1.5, 0.9 Hz, 1H), 7.42 (dd, *J* = 3.3, 1.8 Hz, 1H), 7.15 (ddd, *J* = 7.2, 6.5, 1.5 Hz, 1H), 6.31 (dd, *J* = 3.3, 1.8 Hz, 1H), 6.27 (t, *J* = 3.3 Hz, 1H), 1.40 (s, 9H). minor isomer **3′f**: 9.18 (dt, *J* = 7.2, 1.2 Hz, 1H), 8.68 (s, 1H), 8.20 (t, *J* = 1.9 Hz, 1H), 7.68–7.70 (m, 2H), 7.36 (dd, *J* = 3.4, 2.2 Hz, 1H), 7.18 (ddd, *J* = 7.2, 5.2, 2.8 Hz, 1H), 6.73 (dd, *J* = 3.4, 1.7 Hz, 1H), 1.63 (s, 9H). ^13^C{^1^H} NMR (126 MHz, CDCl_3_): δ major isomer **3f**: 156.8, 152.3, 150.9, 149.0, 135.5, 127.4, 127.2, 126.5, 123.1, 115.6, 115.4, 112.9, 110.6, 83.4, 27.7; minor isomer **3′f**: 155.8, 150.1, 149.3, 148.8, 134.7, 127.3, 126.6, 120.7, 119.9, 119.7, 115.8, 111.6, 109.5, 83.9, 28.0. *m*/*z* (HRMS); found, 312.1337 (MH^+^). C_17_H_18_N_3_O_3_ requires *m*/*z* = 312.1343. ν_max_ (ATR): 3105, 2982, 2930, 749 (C=O), 1674 (C=O), 1636 (C=O), 1495, 1331, 1306, 1254, 1139, 1054, 904, 844, 765, 721 cm^–1^.

##### 4.2.1.7 3-Ferrocenyl-4*H*-pyrido[1,2-*a*]pyrimidin-4-one (**3g**)

From diazonium salt **1b** (130 mg, 0.5 mmol) and ferrocene (**2g**) (93 mg, 0.5 mmol), 19 h, CC (ethyl acetate/petroleum ether = 1:1). Yield: 22 mg (13%) of red solid; mp 202–204 °C. ^1^H NMR (500 MHz, CDCl_3_): δ 9.17 (ddd, *J* = 7.2, 1.5, 0.9 Hz, 1H), 8.58 (s, 1H), 7.69 (ddd, *J* = 9.0, 6.5, 1.5 Hz, 1H), 7.64 (ddd, *J* = 9.0, 1.5, 0.9 Hz, 1H), 7.15 (ddd, *J* = 7.2, 6.5, 1.5 Hz, 1H), 5.04 (t, *J* = 1.9 Hz, 2H), 4.39 (t, *J* = 1.9 Hz, 2H), 4.11 (s, 5H). ^13^C{^1^H} NMR (126 MHz, CDCl_3_): δ 155.7, 150.4, 149.5, 134.5, 127.1, 126.7, 116.3, 115.7, 79.2, 69.5, 69.1, 67.5. *m*/*z* (HRMS); found, 331.0517 (MH^+^). C_18_H_15_FeN_2_O requires *m*/*z* = 331.0528. ν_max_ (ATR): 3083, 2917, 1670 (C=O), 1633 (C=O), 1495, 1459, 1381, 1257, 1100, 809, 789 cm^–1^.

##### 4.2.1.8 3-Phenyl-4*H*-pyrido[1,2-*a*]pyrimidin-4-one (**3h**)

From diazonium salt **1b** (130 mg, 0.5 mmol) and benzene (**2h**) (225 μL, 2.5 mmol), 21 h, CC (ethyl acetate/petroleum ether = 1:1). Yield: 30 mg (27%) of pale-green solid; mp 168–170 °C, lit.^19^ mp 167–169 °C. ^1^H NMR (500 MHz, CDCl_3_): δ 9.21 (ddd, *J* = 7.2, 1.6, 0.9 Hz, 1H), 8.56 (s, 1H), 7.85–7.78 (m, 2H), 7.75 (ddd, *J* = 9.0, 6.5, 1.6 Hz, 1H), 7.70 (dt, *J* = 9.0, 1.5 Hz, 1H), 7.47 (br t, *J* = 7.7 Hz, 2H), 7.37 (tt, *J* = 7.4, 1.3 Hz, 1H), 7.20 (ddd, *J* = 7.2, 6.5, 1.5 Hz, 1H). ^13^C{^1^H} NMR (126 MHz, CDCl_3_): δ 156.8, 153.0, 150.7, 135.7, 134.3, 128.6, 128.6, 127.8, 127.7, 126.6, 117.1, 115.9. *m*/*z* (HRMS); found, 223.0863 (MH^+^). C_14_H_11_N_2_O requires *m*/*z* = 223.0866. Anal. Calcd for C_14_H_10_N_2_O·1/8H_2_O: C, 74.90; H, 4.60; N, 12.48. Found: C, 75.02; H, 4.58; N, 12.25. ν_max_ (ATR): 3133, 2917, 1672 (C=O), 1625 (C=O), 1494, 1472, 1289, 1124, 1074, 899, 774, 696 cm^–1^. The spectral data are in agreement with the literature.^6,19,20^

##### 4.2.1.9 3-(Furan-2-yl)-4-oxo-4*H*-quinolizine-4-carbonitrile (**3i**)

From diazonium salt **1a** (142 mg, 0.5 mmol) and furan (**3a**) (365 μL, 5 mmol), 4 h, CC (ethyl acetate/petroleum ether = 1:1). Yield: 37 mg (31%) of orange solid; mp 196–198 °C. ^1^H NMR (500 MHz, CDCl_3_): δ 9.34 (br dt, *J* = 7.3, 1.0 Hz, 1H), 8.43 (s, 1H), 8.01 (br dt, *J* = 8.9, 1.0 Hz, 1H), 7.70 (ddd, *J* = 8.8, 6.7, 1.2 Hz, 1H), 7.51 (br dd, *J* = 1.7, 0.6 Hz, 1H), 7.37 (br d, *J* = 3.4 Hz, 1H), 7.27 (td, *J* = 7.0, 1.4 Hz, 1H), 6.57 (dd, *J* = 3.4, 1.8 Hz, 1H). ^13^C{^1^H} NMR (126 MHz, CDCl_3_): δ 154.0, 148.5, 143.6, 142.6, 133.8, 133.5, 128.9, 123.6, 117.2, 117.1, 112.5, 111.9, 111.1, 85.9. *m*/*z* (HRMS); found, 237.0657 (MH^+^). C_14_H_9_N_2_O_2_ requires *m*/*z* = 237.0659. ν_max_ (ATR): 2218 (C≡N), 1682 (C=O), 1502, 1246, 1013, 744 cm^–1^.

##### 4.2.1.10 3-Ferrocenyl-4-oxo-4*H*-quinolizin-1-carbonitrile (**3j**)

From diazonium salt **1a** (142 mg, 0.5 mmol) and ferrocene (**2g**) (140 mg, 0.75 mmol), 4 h, CC (ethyl acetate/petroleum ether = 1:3). Yield: 17 mg (10%) of red solid; mp 194–196 °C. ^1^H NMR (500 MHz, CDCl_3_): δ 9.30 (br d, *J* = 7.3 Hz, 1H), 8.04 (s, 1H), 7.93 (br d, *J* = 8.8 Hz, 1H), 7.66 (ddd, *J* = 8.8, 6.7, 1.2 Hz, 1H), 7.21 (td, *J* = 6.9, 1.0 Hz, 1H), 5.04 (t, *J* = 1.9 Hz, 2H), 4.41 (t, *J* = 1.9 Hz, 2H), 4.10 (s, 5H). ^13^C{^1^H} NMR (126 MHz, CDCl_3_): δ 155.6, 143.6, 135.4, 132.7, 128.5, 123.6, 120.6, 117.6, 116.8, 85.3, 80.5, 69.7, 69.6, 68.0, 68.0. *m*/*z* (HRMS); found, 354.0449 (M^+^). C_20_H_14_FeN_2_O requires *m*/*z* = 354.0456. ν_max_ (ATR): 3118, 3088, 2917, 2950, 2206 (C≡N), 1675 (C=O), 1628 (C=O), 1584, 1504, 1459, 1305, 1247, 1150, 1104, 803, 756 cm^–1^.

##### 4.2.1.11 4-Oxo-3-phenyl-4*H*-quinolizin-4-carbonitrile (**3k**)

From diazonium salt **1a** (142 mg, 0.5 mmol) and benzene (**2h**) (225 μL, 2.5 mmol), 18 h, CC (ethyl acetate/petroleum ether = 2:3). Yield: 10 mg (8%) of green solid; mp 178–180 °C, lit.^39^ mp 180–182 °C. ^1^H NMR (500 MHz, CDCl_3_): δ 9.36 (br d, *J* = 7.3 Hz, 1H), 8.05 (s, 1H), 8.01 (br d, *J* = 8.9 Hz, 1H), 7.79–7.70 (m, 3H), 7.46 (br t, *J* = 7.6 Hz, 2H), 7.37 (tt, *J* = 7.4, 1.2 Hz, 1H), 7.27 (td, *J* = 7.2, 1.3 Hz, 1H). ^13^C{^1^H} NMR (126 MHz, CDCl_3_): δ 156.8, 145.0, 138.9, 135.8, 134.2, 129.3, 128.7, 128.7, 128.2, 123.4, 120.8, 117.3, 117.0, 85.4. *m*/*z* (HRMS); found, 247.0865 (MH^+^). C_16_H_11_N_2_O requires *m*/*z* = 247.0866. ν_max_ (ATR): 2207 (C≡N), 1665 (C=O), 1626, 1587, 1500, 1487, 766, 697 cm^–1^. The spectral data are in agreement with the literature.^39^

##### 4.2.1.12 6-(Furan-2-yl)-5*H*-thiazolo[3,2-*a*]pyrimidin-5-one (**3l**)

From diazonium salt **1c** (133 mg, 0.5 mmol) and furan (**2a**) (365 μL, 5 mmol), 4 h, CC (ethyl acetate/petroleum ether = 1:1). Yield: 44 mg (40%) of pale green solid; mp 201–203 °C. ^1^H NMR (500 MHz, CDCl_3_): δ 8.60 (s, 1H), 8.11 (d, *J* = 4.9 Hz, 1H), 7.47 (br dd, *J* = 1.6, 0.6 Hz, 1H), 7.23 (dd, *J* = 3.3, 0.6 Hz, 1H), 7.07 (br d, *J* = 4.9 Hz, 1H), 6.52 (dd, *J* = 3.3, 1.8 Hz, 1H). ^13^C{^1^H} NMR (126 MHz, CDCl_3_): δ 160.3, 155.4, 147.5, 147.2, 142.0, 122.4, 112.4, 112.1, 110.5, 110.3. *m*/*z* (HRMS); found, 219.0230 (MH^+^). C_10_H_7_N_2_O_2_S requires *m*/*z* = 219.0223. ν_max_ (ATR): 3122, 2921, 1673 (C=O), 1504, 1477, 1348, 1275, 1162, 1068, 976, 814, 770, 708 cm^–1^.

##### 4.2.1.13 6-Ferrocenyl-5*H*-thiazolo[3,2-*a*]pyrimidin-5-one (**3m**)

From diazonium salt **1c** (133 mg, 0.5 mmol) and ferrocene (**2g**) (140 mg, 0.75 mmol), 4 h, CC (ethyl acetate/petroleum ether = 1:3). Yield: 18 mg (11%) of orange-red solid; mp 228–230 °C. ^1^H NMR (500 MHz, CDCl_3_): δ 8.25 (s, 1H), 8.08 (d, *J* = 4.9 Hz, 1H), 7.02 (d, *J* = 4.9 Hz, 1H), 4.94 (t, *J* = 1.8 Hz, 2H), 4.35 (t, *J* = 1.8 Hz, 2H), 4.11 (s, 5H). ^13^C{^1^H} NMR (126 MHz, CDCl_3_): δ 159.9, 156.9, 148.6, 122.3, 117.4, 111.9, 78.6, 73.3, 69.6, 69.2, 67.4. *m*/*z* (HRMS); found, 336.0013 (M^+^). C_16_H_12_FeN_2_OS requires *m*/*z* = 336.0020. ν_max_ (ATR): 3104, 2921, 2852, 1658 (C=O), 1505, 1366, 1279, 1081, 927, 801, 723, 658 cm^–1^.

##### 4.2.1.14 6-Phenyl-5*H*-thiazolo[3,2-*a*]pyrimidin-5-one (**3n**)

From diazonium salt **1c** (133 mg, 0.5 mmol) and benzene (**2h**) (225 μL, 2.5 mmol), 18 h, CC (ethyl acetate/petroleum ether = 1:2). Yield: 30 mg (27%) of translucent crystalline solid; mp 146–148 °C, lit.^40^ mp 147–148 °C. ^1^H NMR (500 MHz, CDCl_3_): δ 8.23 (s, 1H), 8.12 (d, *J* = 4.9 Hz, 1H), 7.71 (d, *J* = 7.1 Hz, 2H), 7.45 (t, *J* = 7.6 Hz, 2H), 7.37 (tt, *J* = 7.4, 1.2 Hz, 1H), 7.07 (d, *J* = 4.9 Hz, 1H). ^13^C{^1^H} NMR (126 MHz, CDCl_3_): δ 161.8, 157.8, 151.5, 133.5, 128.7, 128.6, 128.1, 122.7, 118.7, 112.1. *m*/*z* (HRMS); found, 229.0431 (MH^+^). C_12_H_9_N_2_OS requires *m*/*z* = 229.0430. Anal. Calcd for C_12_H_8_N_2_OS·1/50HBF_4_: C, 62.66; H, 3.51; N, 12.18. Found: C, 62.87; H, 3.12; N, 11.97. ν_max_ (ATR): 3118, 1653 (C=O), 1501, 1359, 1283, 715, 698, 654 cm^–1^. Physical data are in agreement with the literature.^40^

##### 4.2.1.15 3-(2,5-Dimethylthiophen-3-yl)-4*H*-pyrido[1,2-*a*]pyrimidin-4-one (**3o**)

From diazonium salt **1b** (130 mg, 0.5 mmol) and 2,5-dimethylthiophene (**2i**) (115 μL, 1 mmol), 18 h, CC (ethyl acetate/petroleum ether = 1:1). Yield: 38 mg (30%) of yellow solid; mp 128–130 °C. ^1^H NMR (500 MHz, CDCl_3_): δ 9.15 (ddd, *J* = 7.2, 1.3, 0.7 Hz, 1H), 8.34 (s, 1H), 7.73 (ddd, *J* = 8.1, 6.5, 1.5 Hz, 1H), 7.68 (ddd, *J* = 8.9, 1.3, 0.7 Hz, 1H), 7.17 (td, *J* = 7.2, 1.5 Hz, 1H), 6.84–6.82 (br m, 1H), 2.44 (s, 3H), 2.40 (s, 3H). ^13^C{^1^H} NMR (126 MHz, CDCl_3_): δ 156.7, 154.0, 150.7, 135.9, 135.6, 135.0, 130.3, 127.7, 127.4, 126.7, 115.8, 113.8, 15.3, 14.4. *m*/*z* (HRMS); found, 257.0739 (MH^+^). C_14_H_13_N_2_OS requires *m*/*z* = 257.0743. ν_max_ (ATR): 3101, 2914, 2854, 1669 (C=O), 1632 (C=O), 1494, 1474, 1325, 1236, 1075, 1027, 964, 849, 773, 740, 654 cm^–1^.

##### 4.2.1.16 3-(3-Methylthiophen-2-yl)-4*H*-pyrido[1,2-*a*]pyrimidin-4-one (**3p**), 3-(4-Methylthiophen-2-yl)-4*H*-pyrido[1,2-*a*]pyrimidin-4-one (**3′p**), and 3-(4-Methylthiophen-3-yl)-4*H*-pyrido[1,2-*a*]pyrimidin-4-one (**3″p**)

From diazonium salt **1b** (130 mg, 0.5 mmol) and 3-methylthiophene (**2j**) (100 μL, 1 mmol), 18 h, CC (ethyl acetate/petroleum ether = 2:3). Yield: 48 mg (40%) of yellow solid; mp 78–130 °C; **3p**:**3′p**:**3″p** = 65:21:14. ^1^H NMR (500 MHz, CDCl_3_): δ major isomer **3p**: 9.17 (br d, *J* = 6.7 Hz, 1H), 8.49 (s, 1H), 7.78–7.72 (m, 1H), 7.72–7.67 (m, 1H), 7.33 (br d, *J* = 5.1 Hz, 1H), 7.20 (td, *J* = 7.0, 1.2 Hz, 1H), 6.95 (d, *J* = 5.1 Hz, 1H), 2.32 (s, 3H); minor isomer **3′p**: 9.18 (br d, *J* = 6.4 Hz, 1H), 8.81 (s, 1H), 7.78–7.72 (m, 1H), 7.72–7.67 (m, 1H), 7.56 (d, *J* = 0.9 Hz, 1H), 7.20 (td, *J* = 7.0, 1.2 Hz, 1H), 6.97 (br s, 1H), 2.32 (s, 3H); minor isomer **3″p**: 9.18 (br d, *J* = 6.7 Hz, 1H), 8.38 (s, 1H), 7.78–7.72 (m, 1H), 7.72–7.67 (m, 1H), 7.42 (d, *J* = 3.3 Hz, 1H), 7.20 (td, *J* = 7.0, 1.2 Hz, 1H), 7.05–7.03 (m, 1H), 2.26 (s, 3H). ^13^C{^1^H} NMR (126 MHz, CDCl_3_): δ major isomer **3p**: 156.5, 154.1, 150.0, 136.3, 136.0, 130.5, 127.9, 126.7, 125.3, 121.9, 116.1, 112.0, 15.6; minor isomers **3′p** and **3″p**: 156.7, 155.4, 154.1, 153.9, 151.0, 150.6, 149.6, 137.9, 137.7, 135.9, 135.5, 135.3, 135.1, 129.3, 127.8, 127.7, 126.8, 126.7, 125.5, 121.9, 116.4, 115.9, 114.0, 112.2, 15.9, 15.4. *m*/*z* (HRMS); found, 243.0588 (MH^+^). C_13_H_11_N_2_OS requires *m*/*z* = 243.0587. ν_max_ (ATR): 3085, 2960, 1657 (C=O), 1627 (C=O), 1483, 1375, 1333, 1125, 1080, 884, 824, 765, 729, 707 cm^–1^.

##### 4.2.1.17 6-(2,5-Dimethylthiophen-3-yl)-5*H*-thiazolo[3,2-*a*]pyrimidin-5-one (**3q**)

From diazonium salt **1c** (133 mg, 0.5 mmol) and 2,5-dimethylthiophene (**2i**) (115 μL, 1 mmol), 4 h, CC (ethyl acetate/petroleum ether = 2:3). Yield: 23 mg (17%) of yellow solid; mp 155–157 °C. ^1^H NMR (500 MHz, CDCl_3_): δ 8.08 (d, *J* = 4.9 Hz, 1H), 8.02 (s, 1H), 7.04 (d, *J* = 4.9 Hz, 1H), 6.76 (br s, 1H), 2.43 (s, 3H), 2.38 (s, 3H). ^13^C{^1^H} NMR (126 MHz, CDCl_3_): δ 161.6, 157.6, 152.4, 135.9, 135.1, 129.3, 127.4, 122.6, 115.0, 112.0, 15.3, 14.3. *m*/*z* (HRMS); found, 263.0302 (MH^+^). C_12_H_11_N_2_OS_2_ requires *m*/*z* = 263.0307. ν_max_ (ATR): 3069, 2918, 2854, 1650 (C=O), 1496, 1476, 1435, 1345, 1218, 1136, 1023, 844, 777, 742, 675 cm^–1^.

##### 4.2.1.18 3-(Furan-2-yl)-7-phenyl-4*H*-pyrimido[1,2-*b*]pyridazin-4-one (**3r**)

From diazonium salt **1d** (169 mg, 0.5 mmol) and furan (**2a**) (365 μL, 5 mmol), 18 h, CC (ethyl acetate/petroleum ether = 3:2). Yield: 75 mg (52%) of yellow solid; mp 216–218 °C. ^1^H NMR (500 MHz, CDCl_3_): δ 8.83 (s, 1H), 8.17–8.05 (m, 2H), 7.93 (d, *J* = 9.4 Hz, 1H), 7.87 (d, *J* = 9.4 Hz, 1H), 7.60–7.42 (m, 5H), 6.57 (dd, *J* = 3.4, 1.8 Hz, 1H). ^13^C{^1^H} NMR (126 MHz, CDCl_3_): δ 154.7, 153.4, 147.4, 146.8, 146.5, 142.7, 135.2, 133.9, 131.5, 129.4, 127.6, 124.9, 114.3, 112.7, 112.5. *m*/*z* (HRMS); found, 290.0922 (MH^+^). C_17_H_12_N_2_O_2_ requires *m*/*z* = 290.0924. ν_max_ (ATR): 3106, 1699 (C=O), 1540, 1474, 1346, 1328, 1251, 1007, 838, 778, 758, 726, 688 cm^–1^.

#### 4.2.2 Synthesis of 3-(Furan-2-yl)-4*H*-pyrido[1,2-*a*]pyrimidin-4-one (**3a**) on a Larger Scale

A 16 mL vial with a screw cap and septum was charged with diazonium tetrafluoroborate **1b** (520 mg, 2.0 mmol), furan **2** (1.46 mL, 20 mmol), **EY-Na**_**2**_ (14 mg, 20 μmol), acetonitrile (7.2 mL), and water (0.8 mL). The vial was stopped, carefully degassed, and purged with nitrogen using the freeze–pump–thaw technique. Then the vial was mounted into a photoreactor and irradiated with green light (510 nm) at 20 °C for 4 h. The reaction mixture was purified by CC (neutral alumina, ethyl acetate/petroleum ether = 1:1). Fractions containing the product were combined and evaporated in vacuo to give **3a**. Yield: 241 mg (57%) of yellow solid with physical and spectral data in agreement with the data of **3a** obtained on a 0.5 mmol scale (see Section 4.2.1).

#### 4.2.3 Preparation of Single Crystals of Compound **3n**

A 1 mL vial was charged with compound **3n** (5 mg) and CDCl_3_ (150 μL). Then, the 1 mL vial containing a solution of **3n** in CDCl_3_ was placed into a 5 mL vial containing petroleum ether (450 μL). The outer vial (5 mL vial) was stopped and left to stand at room temperature for 3 days under vapor diffusion conditions. Since no crystal growth was observed after 3 days, the stopper was punctured several times with a syringe needle to allow slow evaporation of the solvents. After standing at room temperature for two more days, the solvents evaporated completely to leave a translucent crystalline residue that contained single crystals of **3n**. One of these crystals was then selected and used for X-ray diffraction analysis.

## Data Availability

The data underlying this study are available in the published article and its Supporting Information.

## Supporting Information Available

Experimental details (reaction setup), copies of ^1^H NMR, ^13^C NMR, and 2D NMR spectra, copies of IR spectra, structure determination by NMR, elucidation of the reaction mechanism, and X-ray diffraction analysis data for **3n** (PDF)

## Dedication

This work is dedicated to Professor Emeritus Branko Stanovnik, University of Ljubljana, on the occasion of his 85th birthday.
