# Supplementary material for: Catalytic Photoredox C–H Arylation of 4-Oxo-4H-pyrido[1,2-a]pyrimidine-3-diazonium Tetrafluoroborates and Related Heteroaryl Diazonium Salts
Source: J Org Chem. 2023 Sep 7;88(19):13934–45. doi: 10.1021/acs.joc.3c01517 (PMC10563132; doi:10.1021/acs.joc.3c01517)
Supplement: Supplementary file 1 — jo3c01517_si_001.pdf [file jo3c01517_si_001.pdf]

Supporting Information

for

Catalytic Photoredox C–H Arylation of 4-Oxo-4*H*-pyrido[1,2-*a*]pyrimidine-3-diazonium Tetrafluoroborates and Related Heteroaryl Diazonium Salts

Kris Antolinc, Helena Brodnik, Uroš Grošelj, Bogdan Štefane, Nejc Petek*, and Jurij Svete*

University of Ljubljana, Faculty of Chemistry and Chemical Technology, Večna pot 113, SI-1000 Ljubljana, Slovenia

E-mail: nejc.petek@fkkt.uni-lj.si and jurij.svete@fkkt.uni-lj.si

Table of Contents

1. Experimental details (reaction setup) S2
2. Copies of ^{1}H and ^{13}C NMR spectra of compounds **3a**–**n**. S3
3. Copies of IR spectra of compounds **3a**–**n** S23
4. Structure determination by NMR. S28
5. Elucidation of the reaction mechanism. S40
6. X-Ray diffraction analysis data for compound **3n**. S50
7. References S52

1. Experimental details (reaction setup).

Photocatalytic transformations were carried out on a custom made photoreactor using LED illumination, magnetic stirring, and cooling block to sustain a reaction temperature of 20 °C. Vials were placed around 2 mm above LEDs with no filter applied. LEDs used: 450 nm (ProLight Opto, PM2B-3-LBS-SD, blue, wavelength of peak intensity 445–455 nm, 39.8–51.7 lm) and 510 nm (Seoul Semiconductor, G42180, green, wavelength of peak intensity 505–520 nm, 70 lm). The photochemical reaction setup is shown in Figure S1.

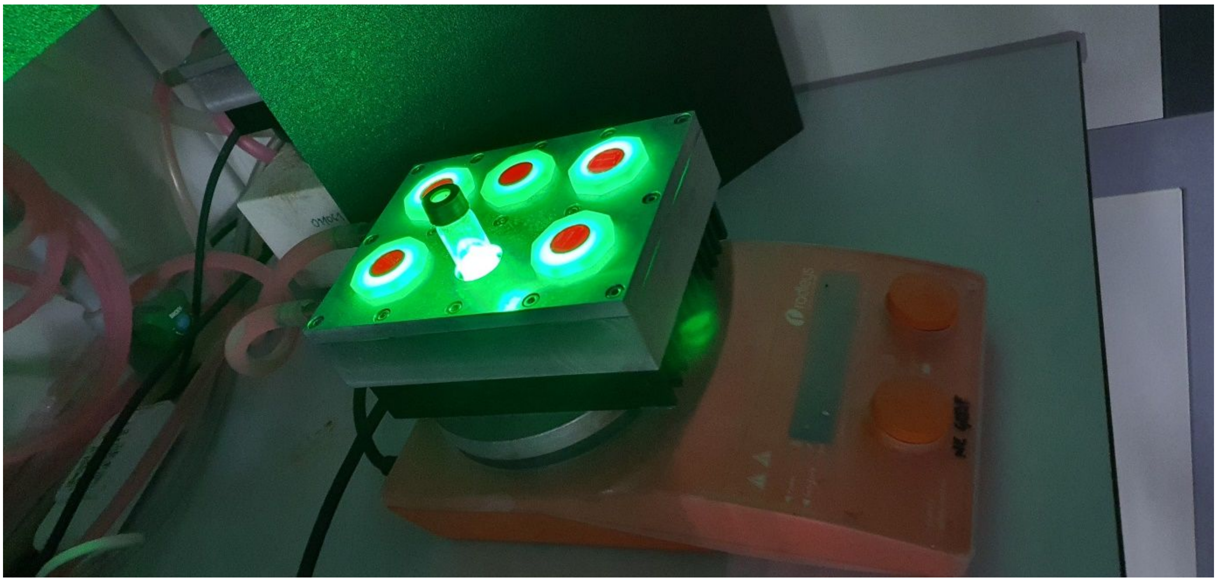

Figure S1. Photochemical reaction setup.

2. Copies of ^{1}H and ^{13}C NMR spectra of novel compounds 3a–r.

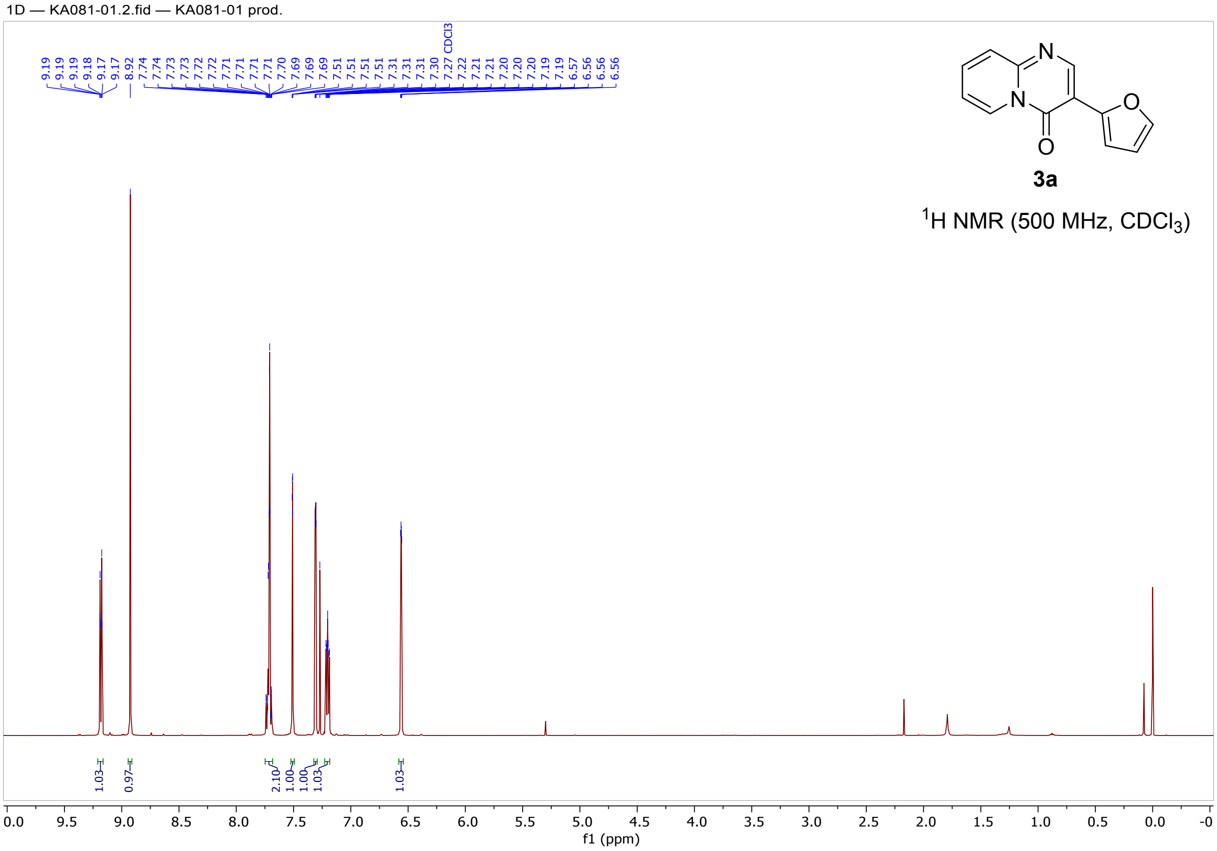

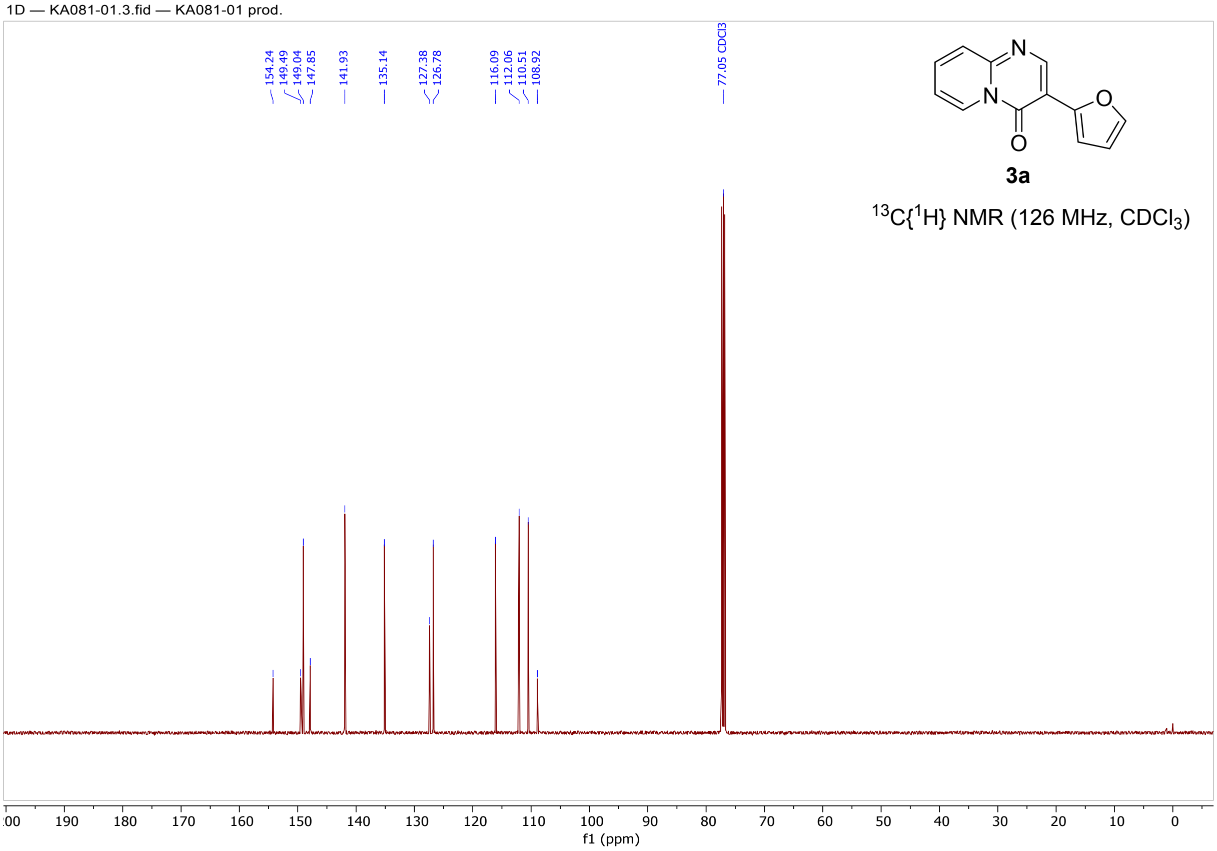

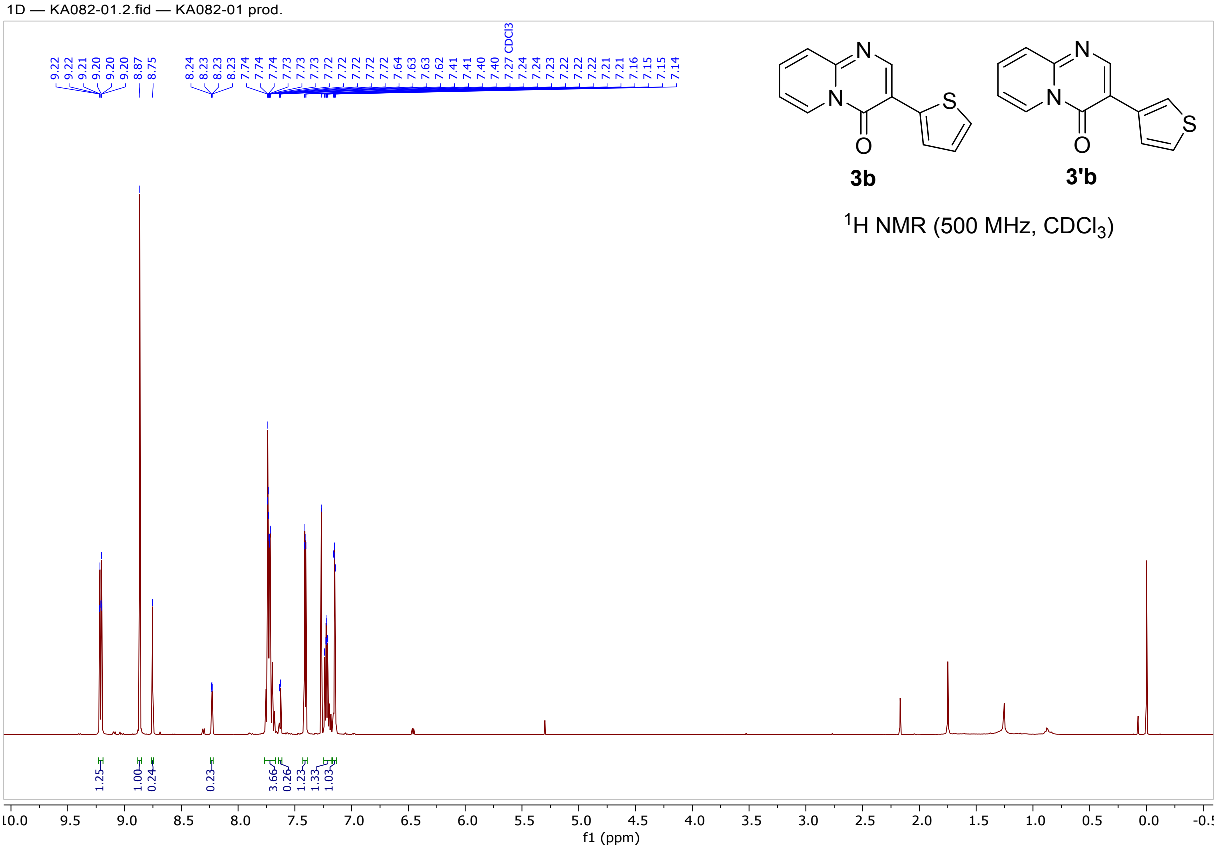
1D — KA082-01.2.fid — KA082-01 prod.
3b
3'b
1H NMR (500 MHz, CDCl3)
f1 (ppm)

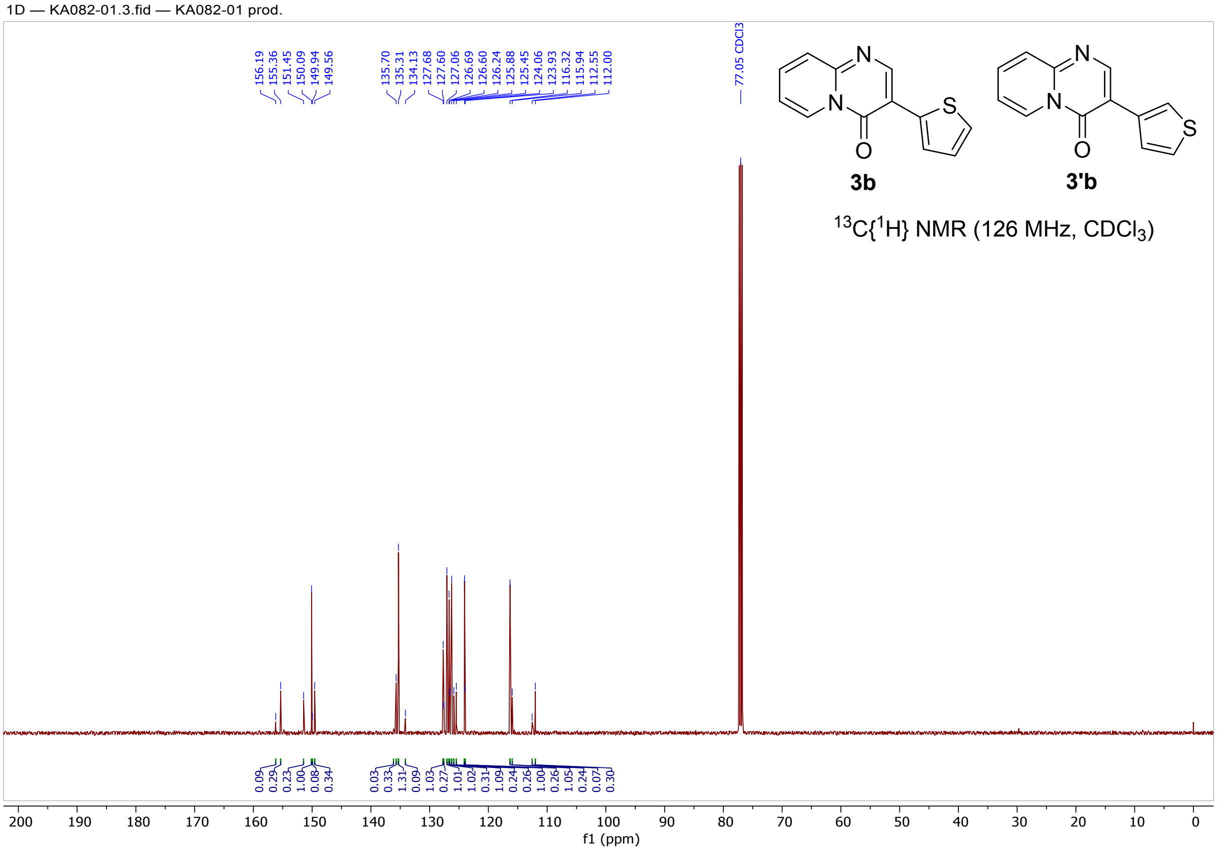
1D — KA082-01.3.fid — KA082-01 prod.
77.05 CDCl3
3b
3'b
13C{1H} NMR (126 MHz, CDCl3)
f1 (ppm)

COSY — KA082-02.2.ser — COSY tiofena

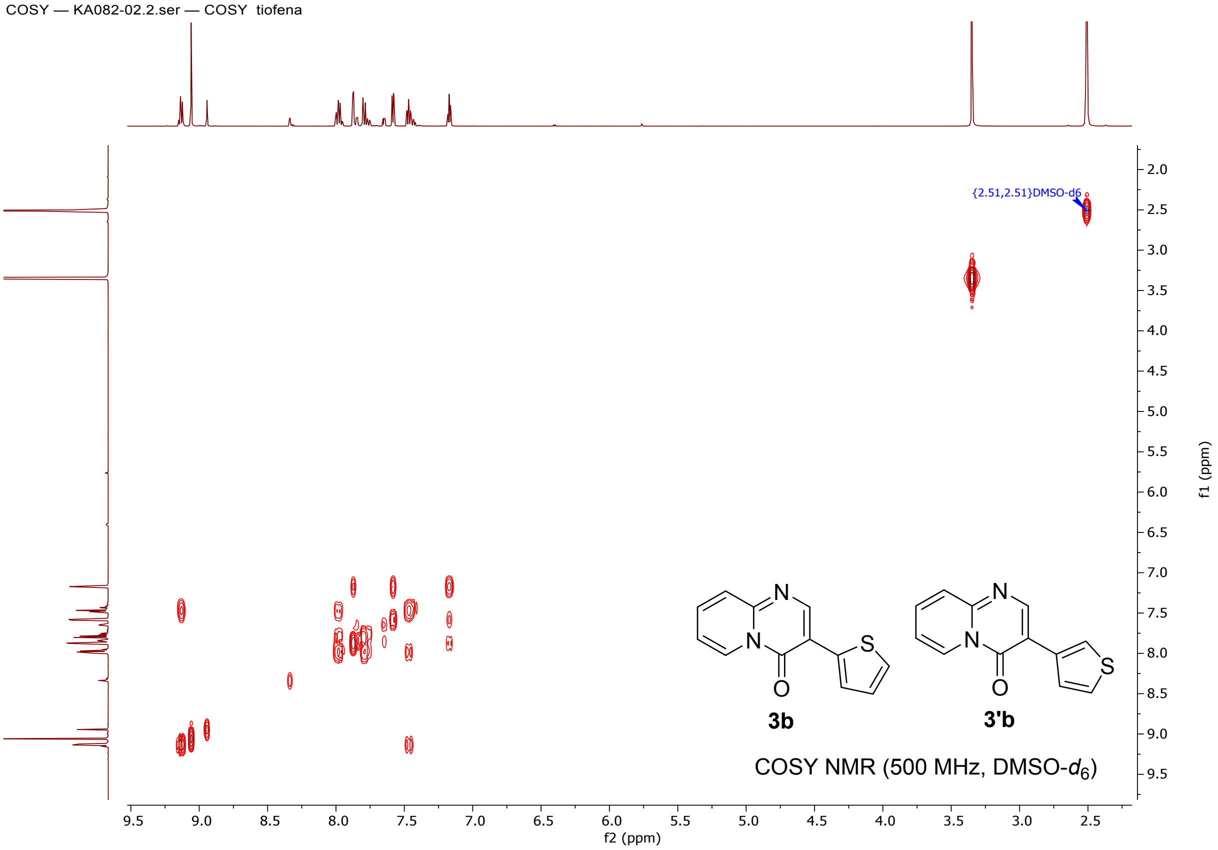

HSQC — KA082-02.3.ser — hsqc tiofena

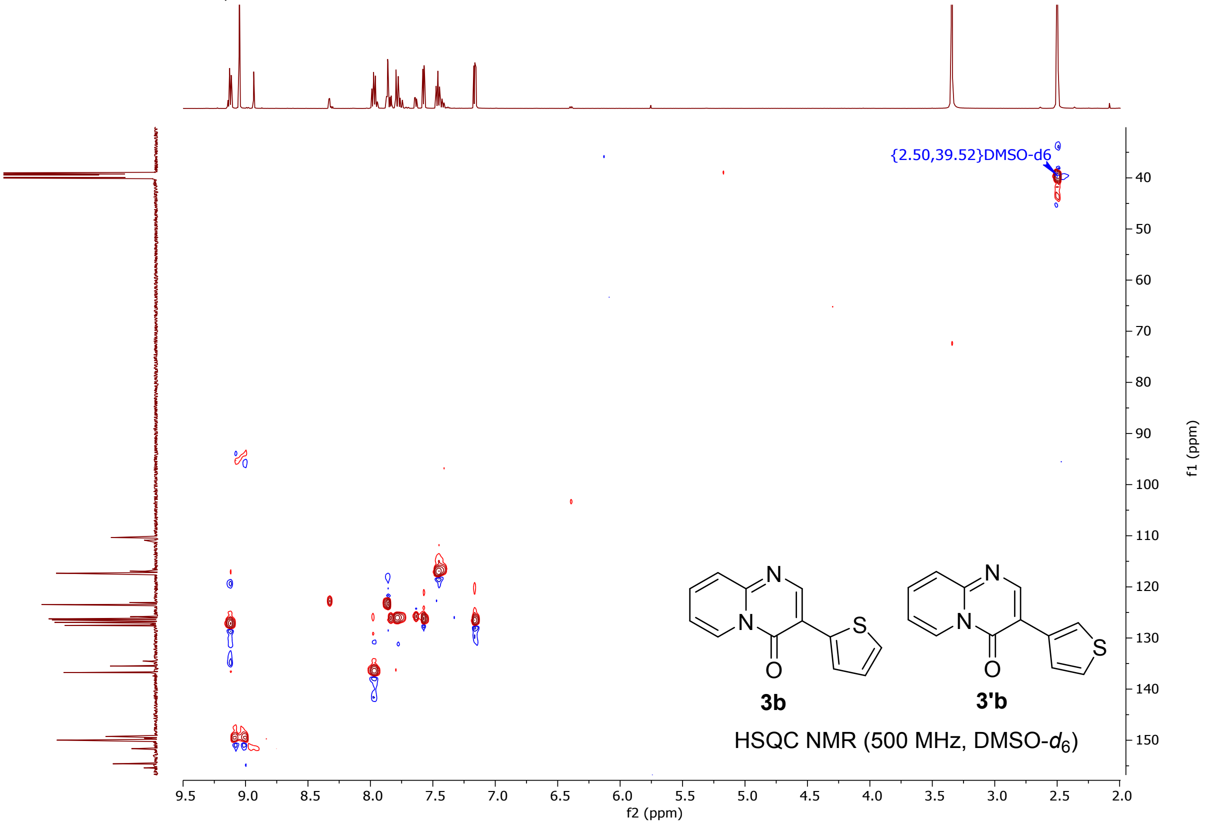

HMBC — KA082-02.4.ser — hmbc tiofena

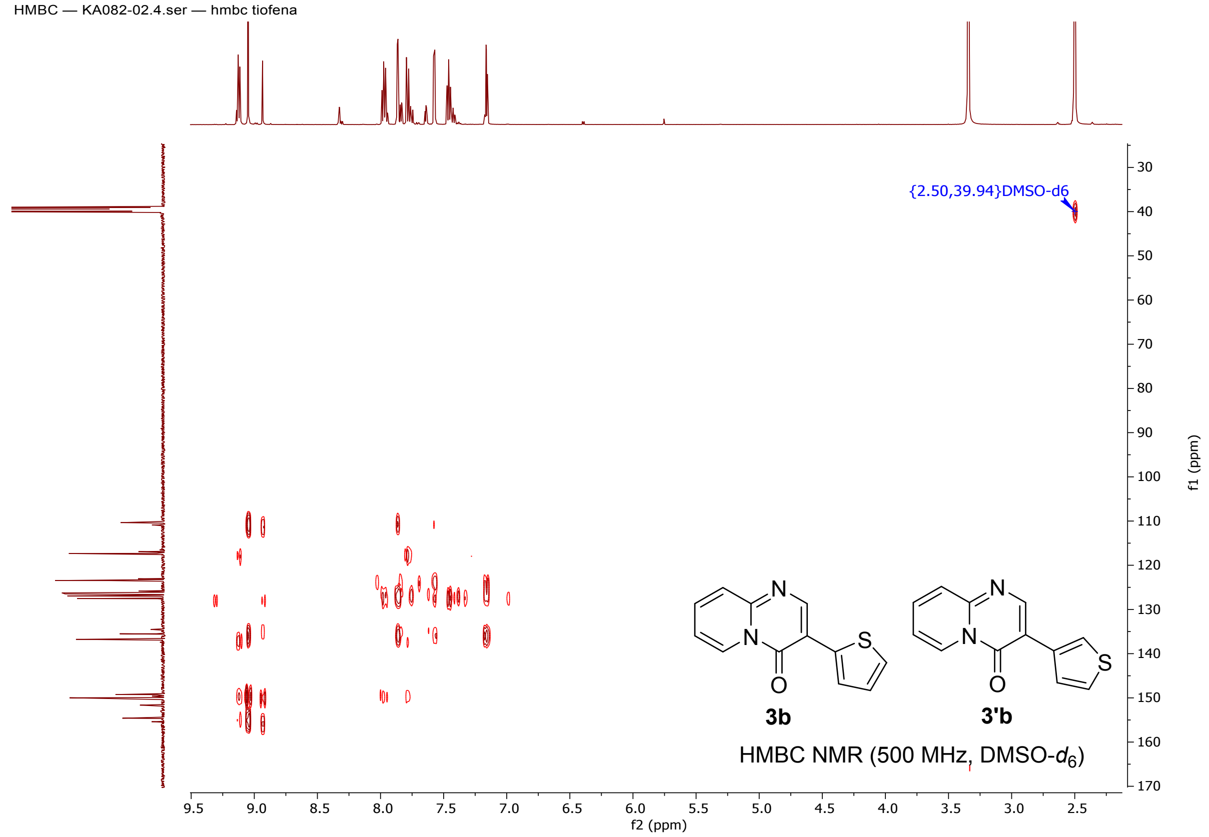

NOESY — KA082-02.5.ser — noesy tiofena

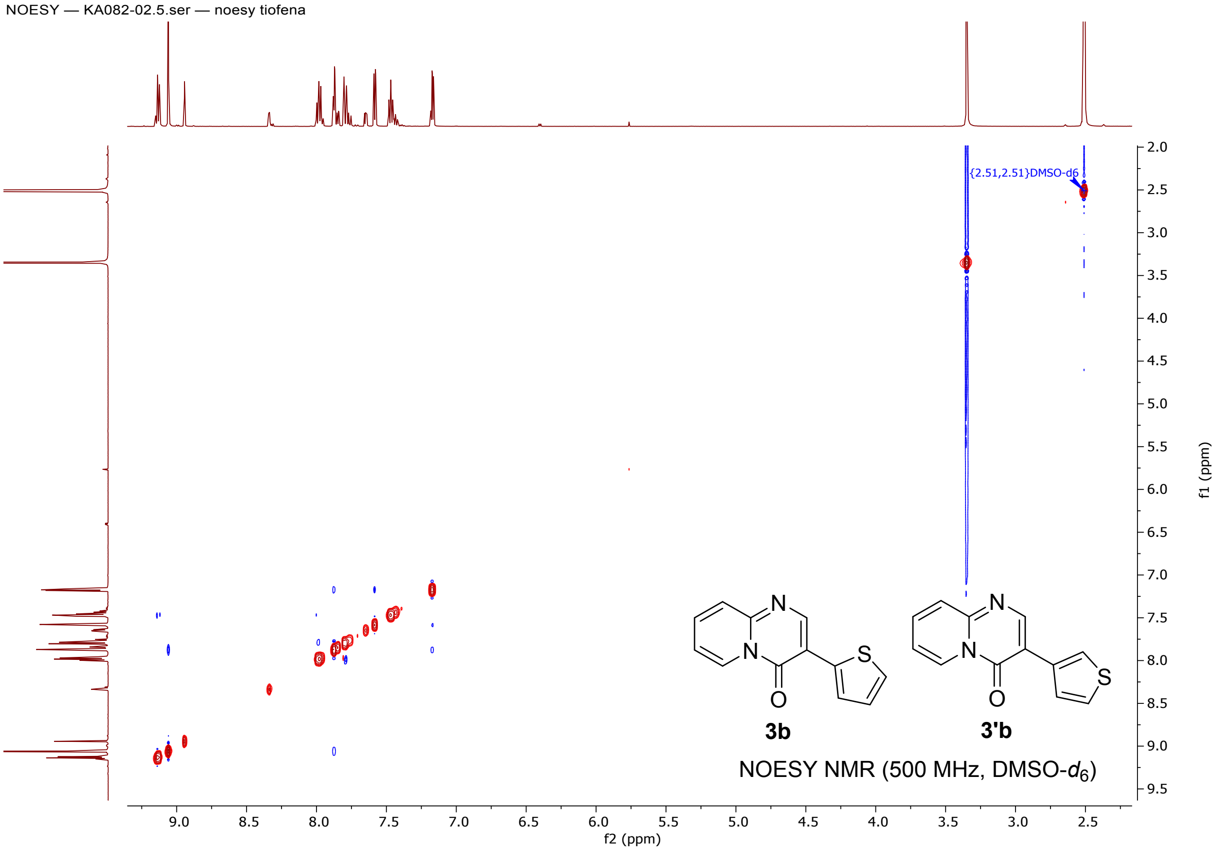

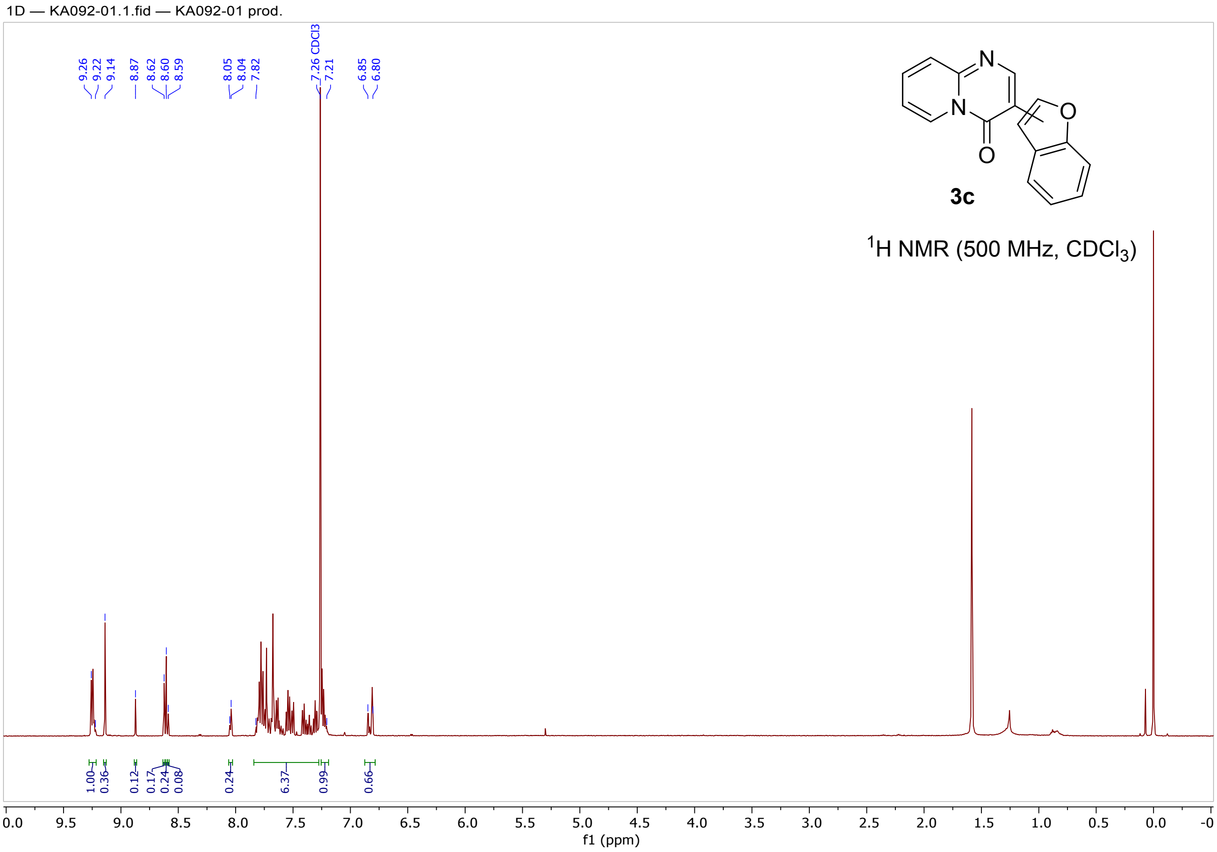

1D — KA092-01.1.fid — KA092-01 prod.
9.26
9.22
9.14
8.87
8.62
8.60
8.59
8.05
8.04
7.82
7.26 CDCl3
7.21
6.85
6.80
1.00
0.36
0.12
0.17
0.24
0.08
0.24
6.37
0.99
0.66
N
N
O
O
3c
1H NMR (500 MHz, CDCl3)
f1 (ppm)

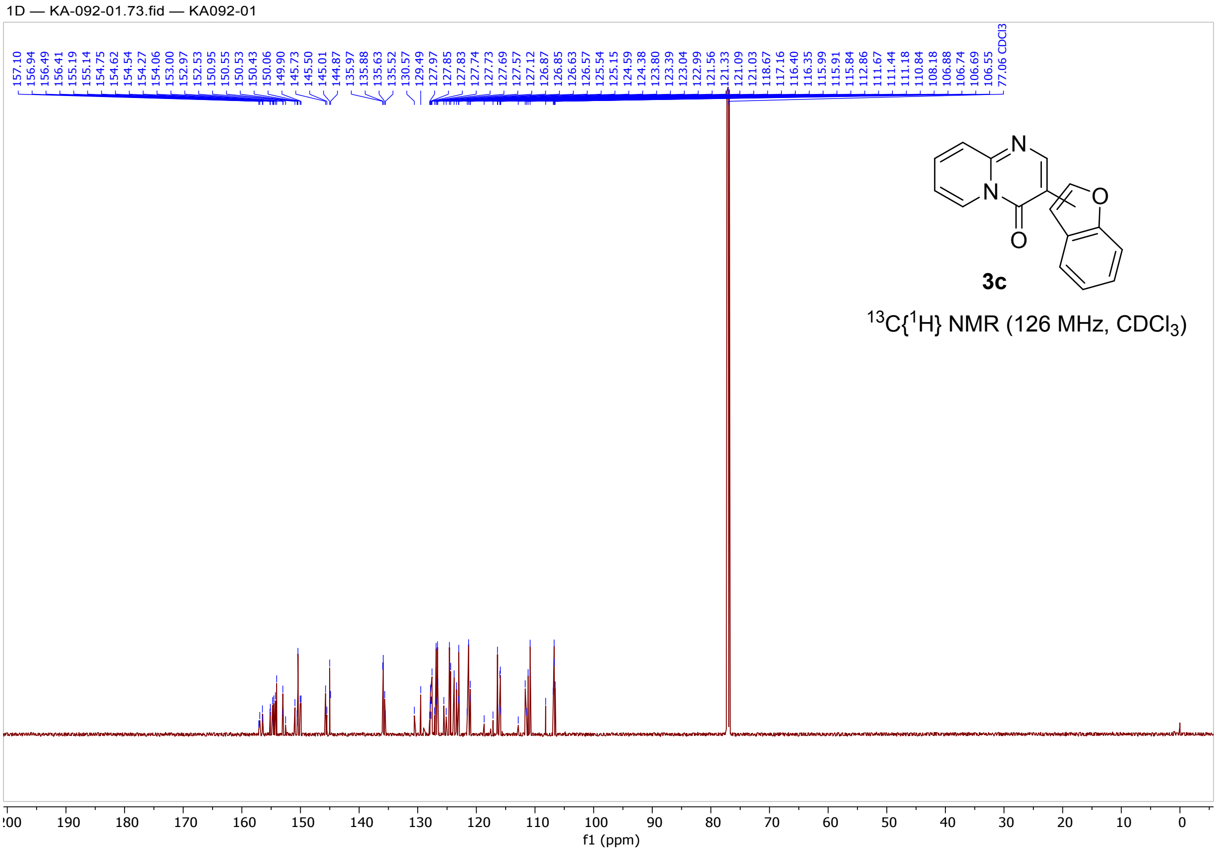

1D — KA-092-01.73.fid — KA092-01
157.10
156.94
156.49
156.41
155.19
155.14
154.75
154.62
154.54
154.27
154.06
153.00
152.97
152.53
150.95
150.55
150.53
150.43
150.06
149.90
145.73
145.50
145.01
144.87
135.97
135.88
135.63
135.52
130.57
129.49
127.97
127.85
127.83
127.74
127.73
127.69
127.57
127.12
126.87
126.85
126.63
126.57
125.54
125.15
124.59
124.38
123.80
123.39
123.04
122.99
121.56
121.33
121.09
121.03
118.67
117.16
116.40
116.35
115.99
115.91
115.84
112.86
111.67
111.44
111.18
110.84
108.18
106.88
106.74
106.69
106.55
77.06 CDCl3
N
N
O
O
3c
13C{1H} NMR (126 MHz, CDCl3)
f1 (ppm)

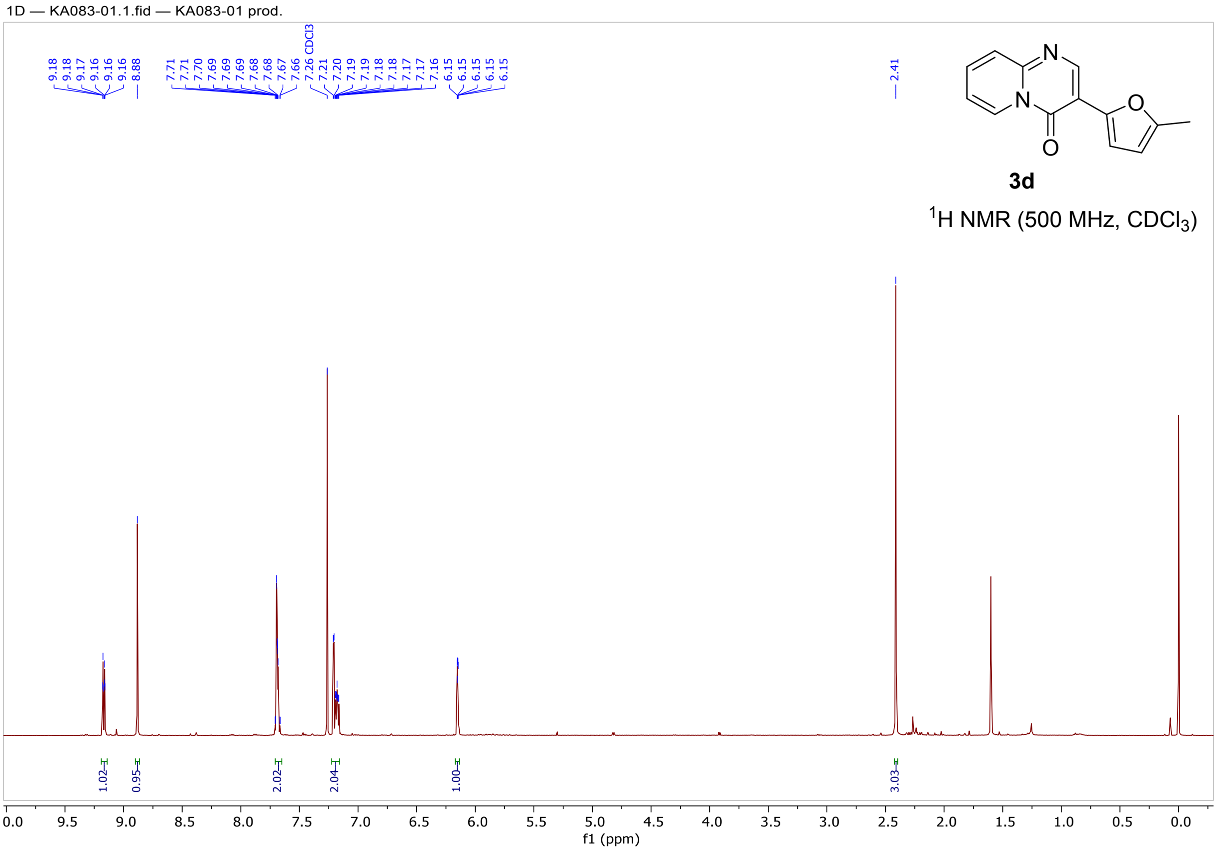
1D — KA083-01.1.fid — KA083-01 prod.
9.18 9.18 9.17 9.16 9.16 9.16 8.88 7.71 7.71 7.70 7.69 7.69 7.69 7.68 7.68 7.67 7.66 7.26 CDCl3 7.21 7.20 7.19 7.19 7.18 7.18 7.17 7.17 7.16 6.15 6.15 6.15 6.15 6.15 2.41
3d
1H NMR (500 MHz, CDCl3)
1.02 0.95 2.02 2.04 1.00 3.03
f1 (ppm)

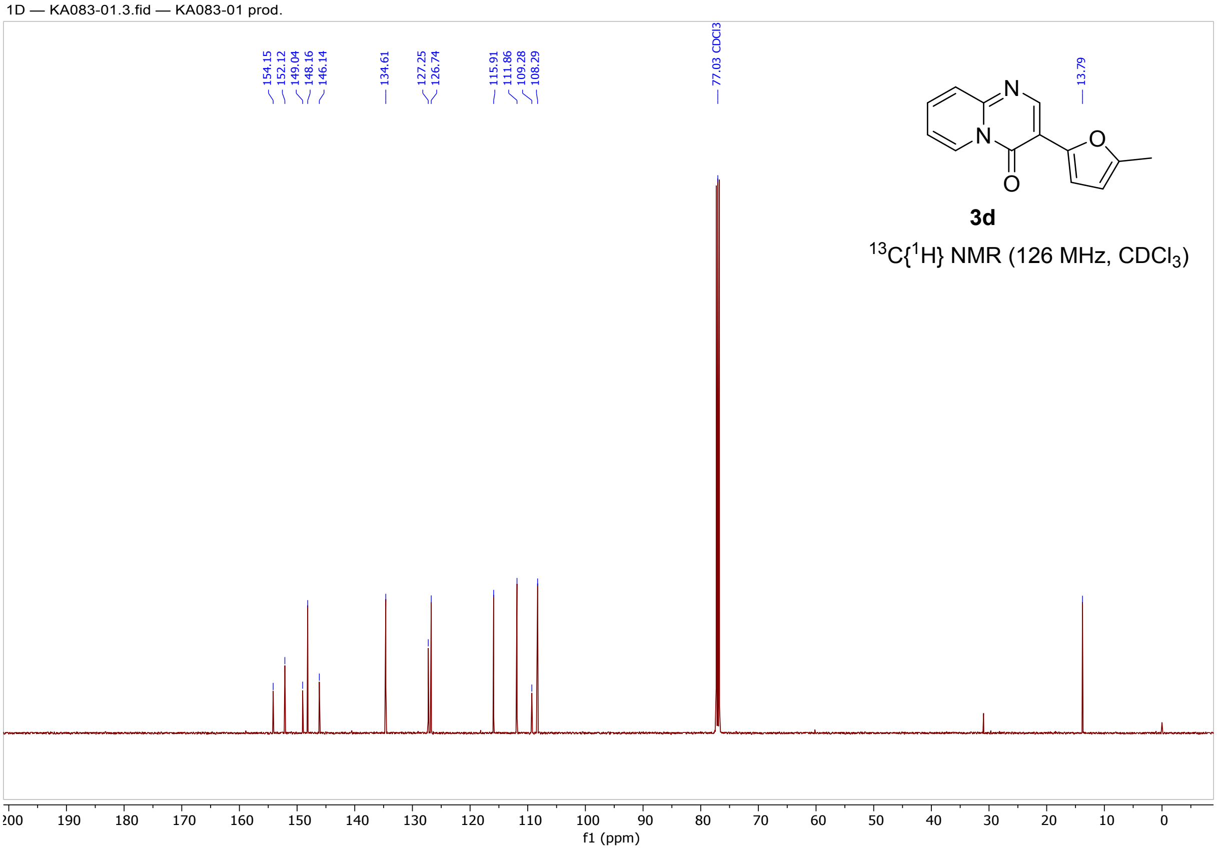
1D — KA083-01.3.fid — KA083-01 prod.
154.15 152.12 149.04 148.16 146.14 134.61 127.25 126.74 115.91 111.86 109.28 108.29 77.03 CDCl3 13.79
3d
13C{1H} NMR (126 MHz, CDCl3)
f1 (ppm)

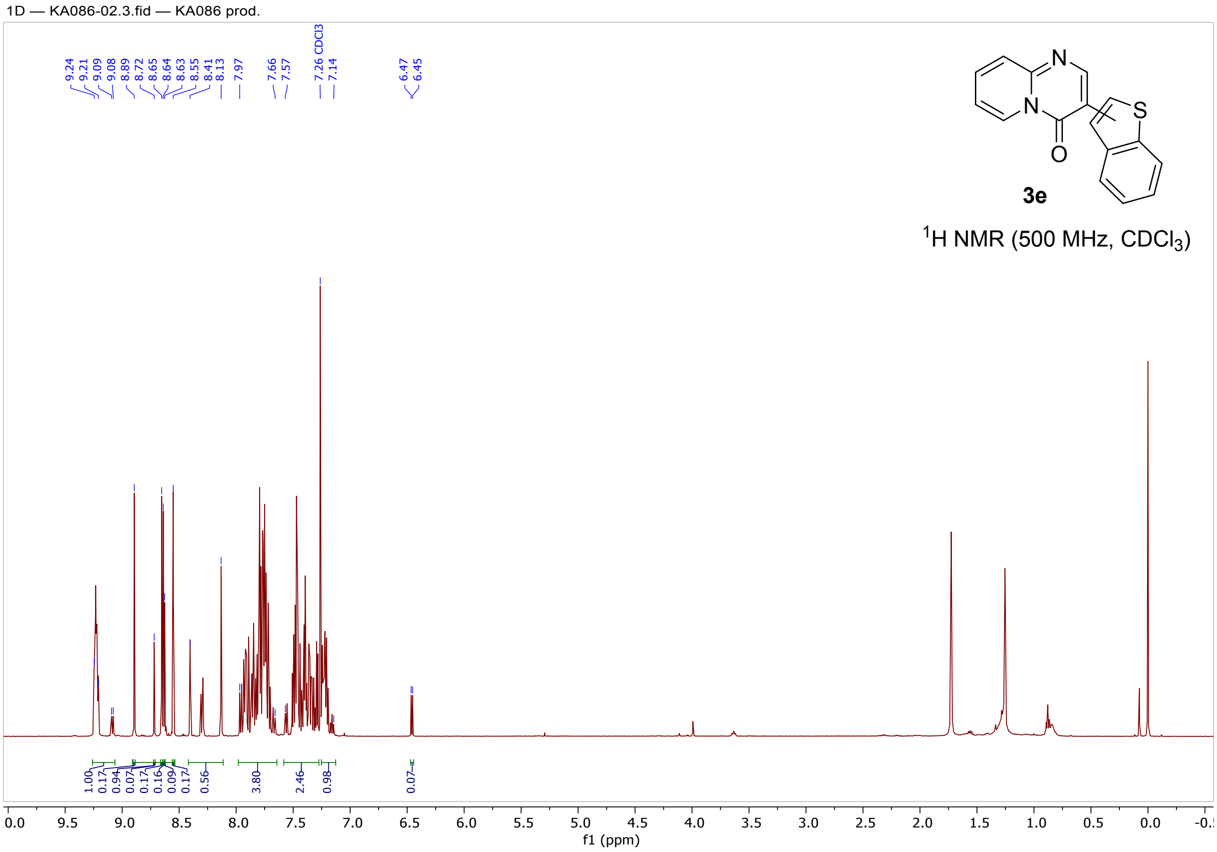
1D — KA086-02.3.fid — KA086 prod.
9.24 9.21 9.09 9.08 8.89 8.72 8.65 8.64 8.63 8.55 8.41 8.13 7.97 7.66 7.57 7.26 CDCl3 7.14 6.47 6.45
3e
^{1}H NMR (500 MHz, CDCl$_3$)
1.00 0.17 0.94 0.07 0.17 0.16 0.09 0.17 0.56 3.80 2.46 0.98 0.07
f1 (ppm)

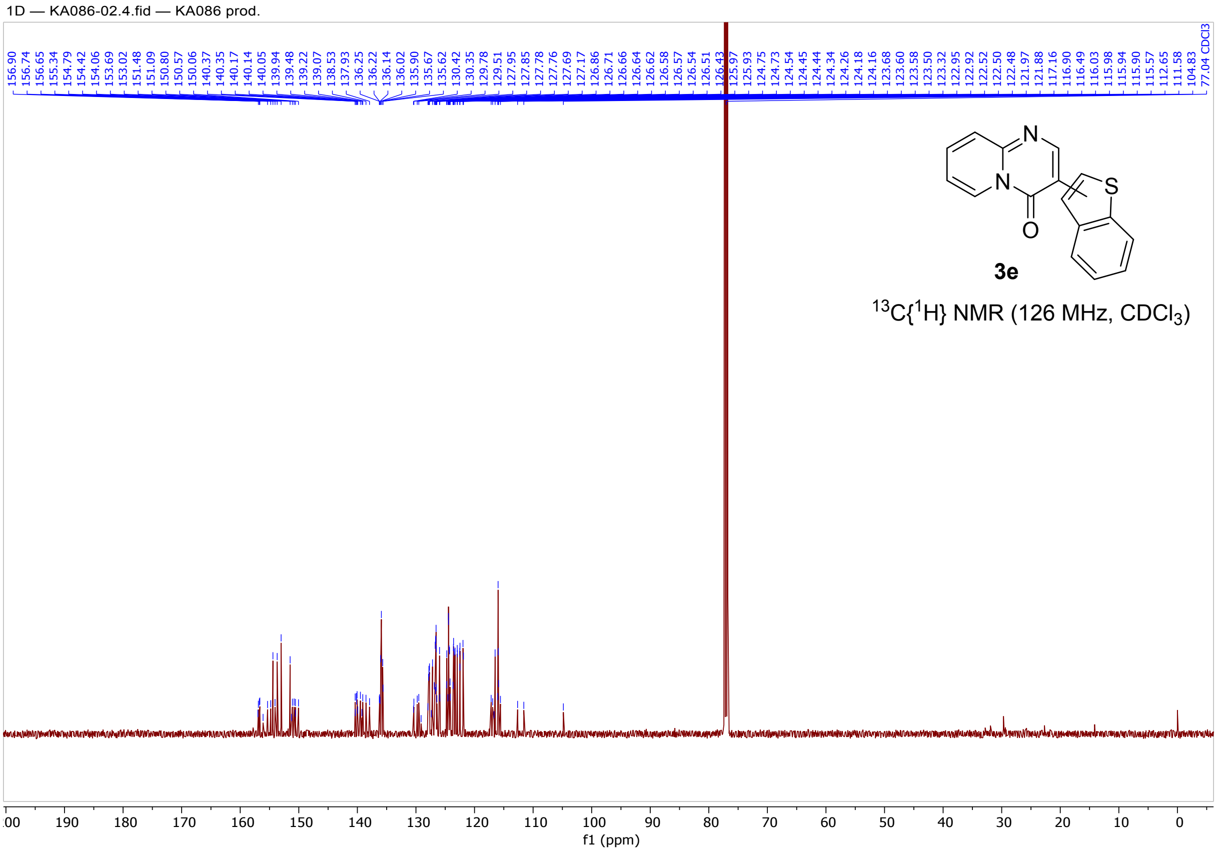
1D — KA086-02.4.fid — KA086 prod.
156.90 156.74 156.65 155.34 154.79 154.42 154.06 153.69 153.02 151.48 151.09 150.80 150.57 150.06 140.37 140.35 140.17 140.14 140.05 139.94 139.48 139.22 139.07 138.53 137.93 136.25 136.22 136.14 136.02 135.90 135.67 135.62 130.42 130.35 129.78 129.51 127.95 127.85 127.78 127.76 127.69 127.17 126.86 126.71 126.66 126.64 126.62 126.58 126.57 126.54 126.51 126.43 125.97 125.93 124.75 124.73 124.54 124.45 124.44 124.34 124.26 124.18 124.16 123.68 123.60 123.58 123.50 123.32 122.95 122.92 122.52 122.50 122.48 121.97 121.88 117.16 116.90 116.49 116.03 115.98 115.94 115.90 115.57 112.65 111.58 104.83 77.04 CDCl3
3e
^{13}C{^{1}H} NMR (126 MHz, CDCl$_3$)
f1 (ppm)

1D — KA088-01.2.fid — KA088 prod.

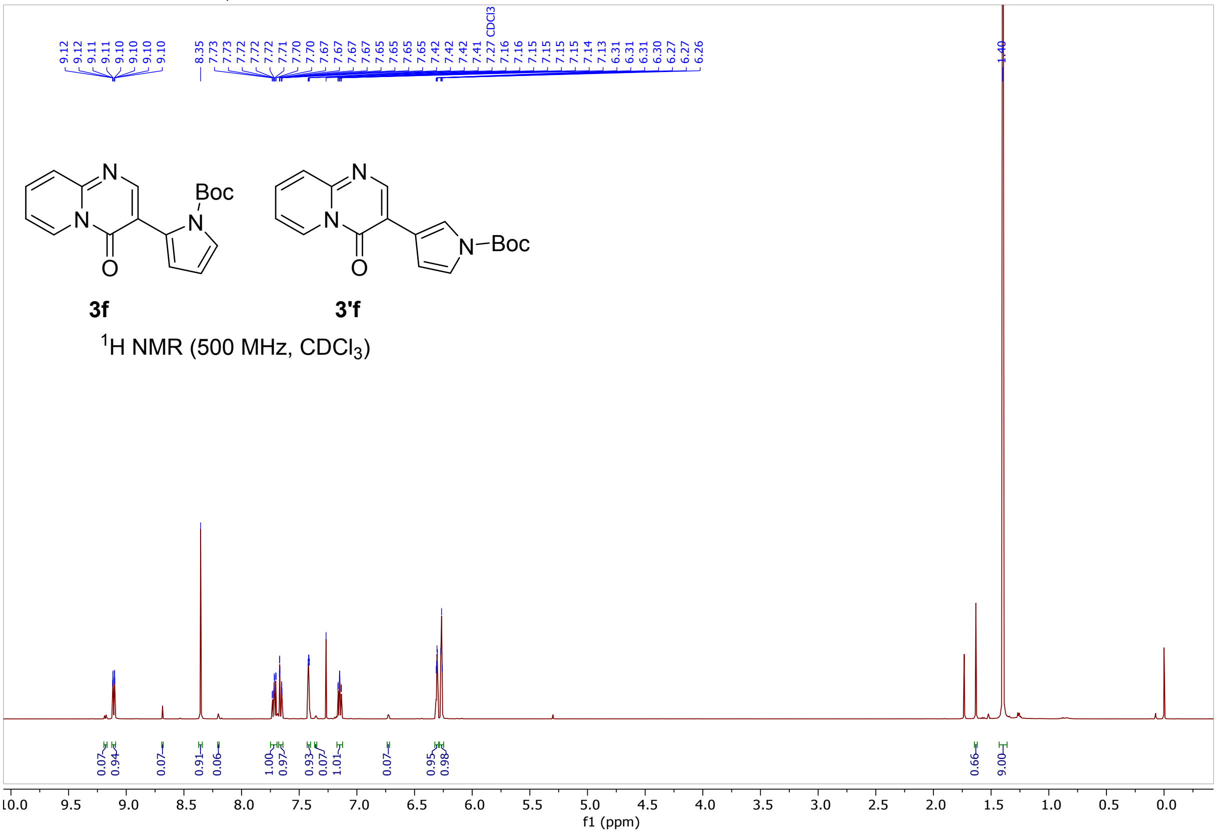

1D — KA088-01.3.fid — KA088 prod.

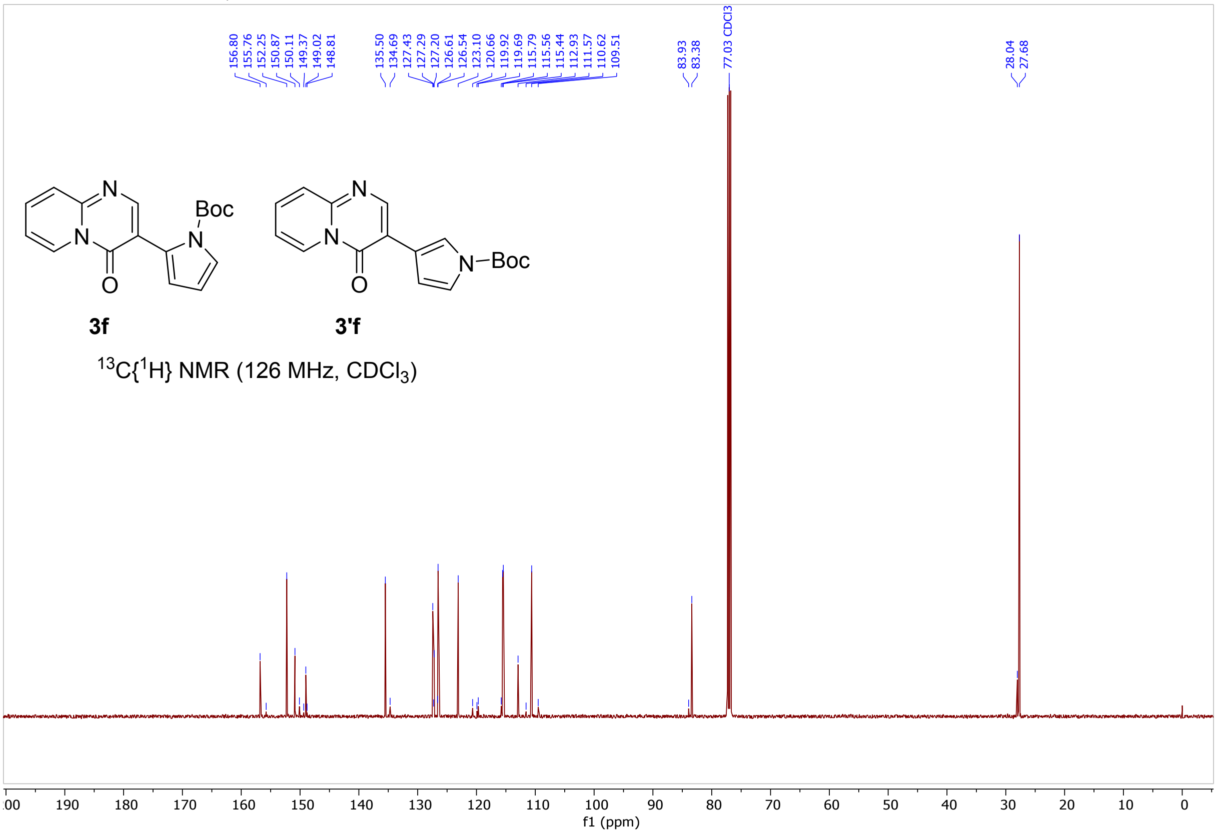

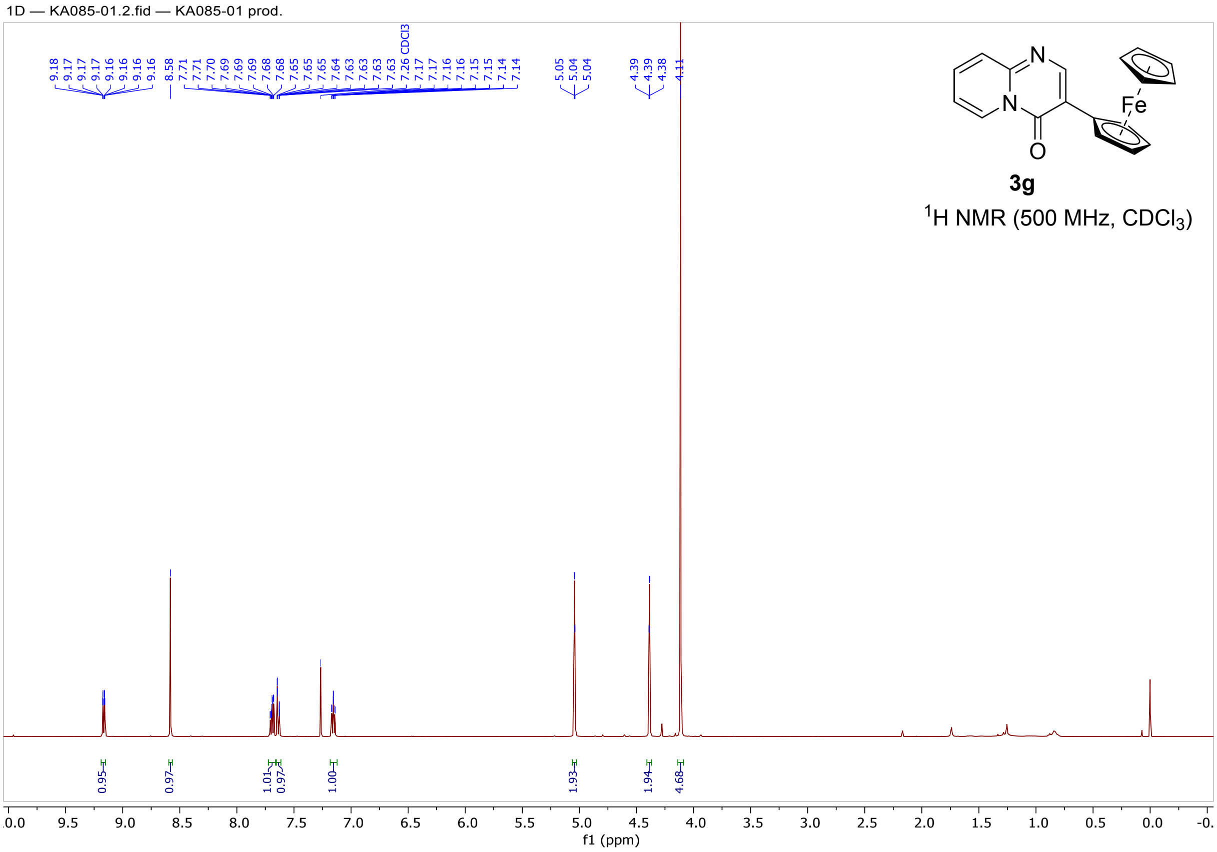
1D — KA085-01.2.fid — KA085-01 prod.
9.18 9.17 9.17 9.17 9.16 9.16 9.16 8.58 7.71 7.71 7.70 7.69 7.69 7.68 7.68 7.65 7.65 7.65 7.64 7.63 7.63 7.63 7.63 7.26 CDCl3 7.17 7.17 7.16 7.16 7.15 7.15 7.14 7.14
5.05 5.04 5.04
4.39 4.39 4.38 4.11
0.95 0.97 1.01 0.97 1.00 1.93 1.94 4.68
N
N
O
Fe
3g
1H NMR (500 MHz, CDCl3)
f1 (ppm)

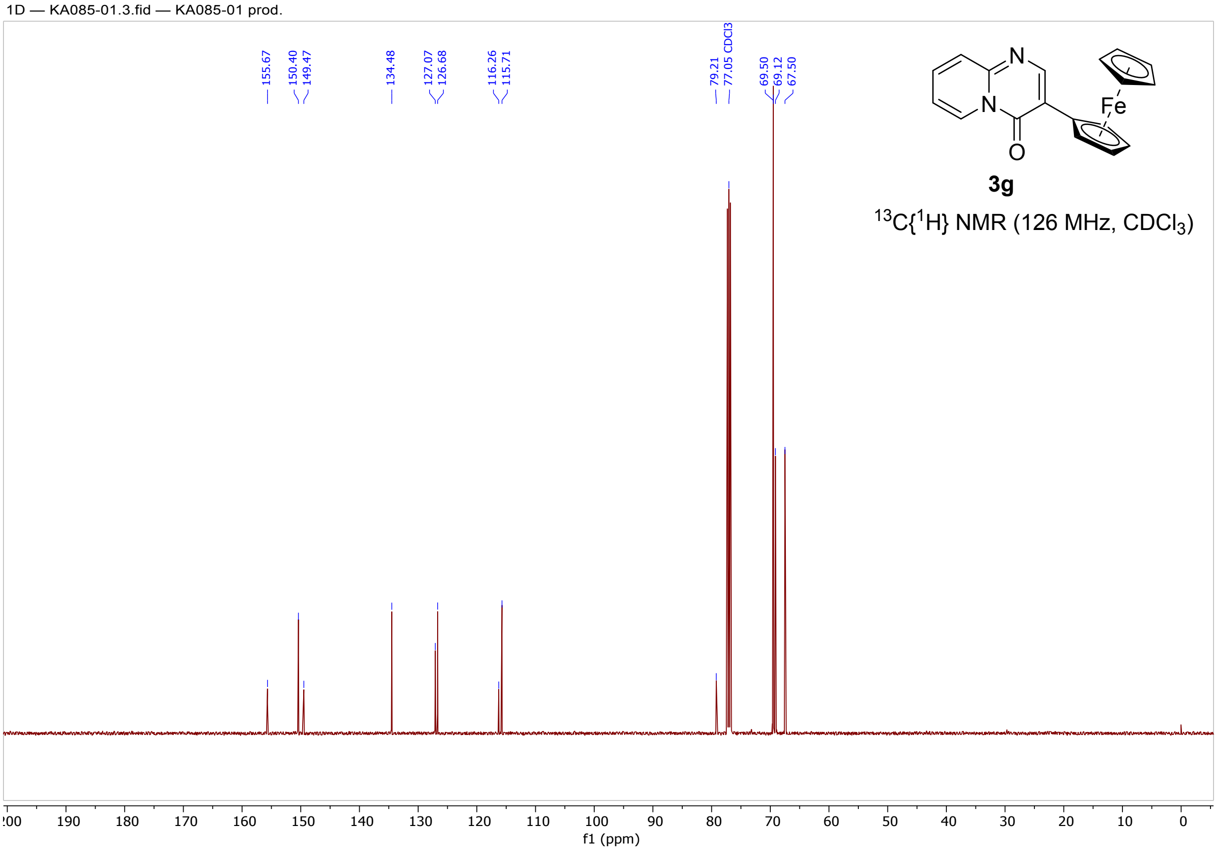
1D — KA085-01.3.fid — KA085-01 prod.
155.67 150.40 149.47 134.48 127.07 126.68 116.26 115.71
79.21 77.05 CDCl3 69.50 69.12 67.50
N
N
O
Fe
3g
13C{1H} NMR (126 MHz, CDCl3)
f1 (ppm)

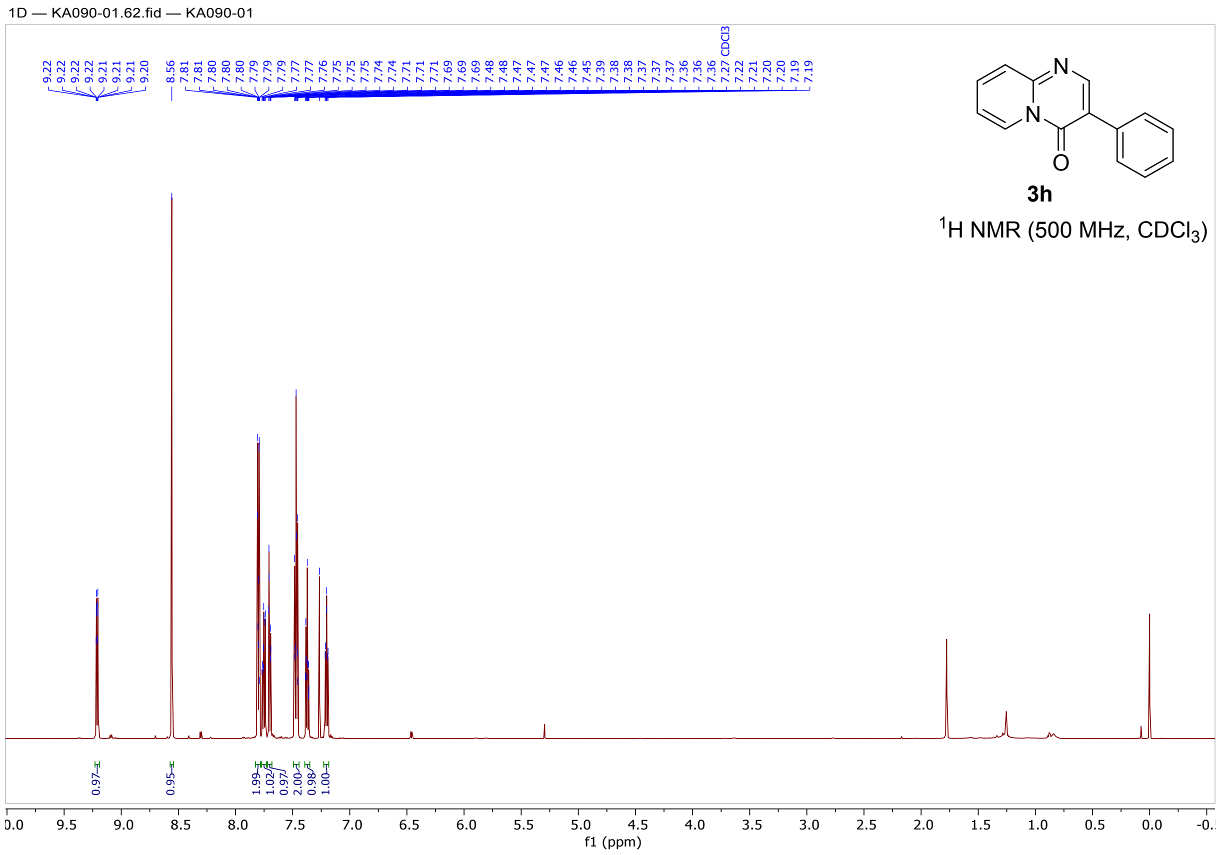
1D — KA090-01.62.fid — KA090-01
9.22 9.22 9.22 9.22 9.21 9.21 9.21 9.20
8.56
7.81 7.81 7.80 7.80 7.80 7.79 7.79 7.79 7.77 7.77 7.76 7.75 7.75 7.75 7.74 7.74 7.71 7.71 7.71 7.69 7.69 7.69 7.48 7.48 7.47 7.47 7.47 7.46 7.46 7.45 7.39 7.38 7.38 7.37 7.37 7.37 7.36 7.36 7.36 7.27 CDCl3 7.22 7.21 7.20 7.20 7.19 7.19
N
N
O
3h
1H NMR (500 MHz, CDCl3)
0.97
0.95
1.99
1.02
0.97
2.00
0.98
1.00
f1 (ppm)

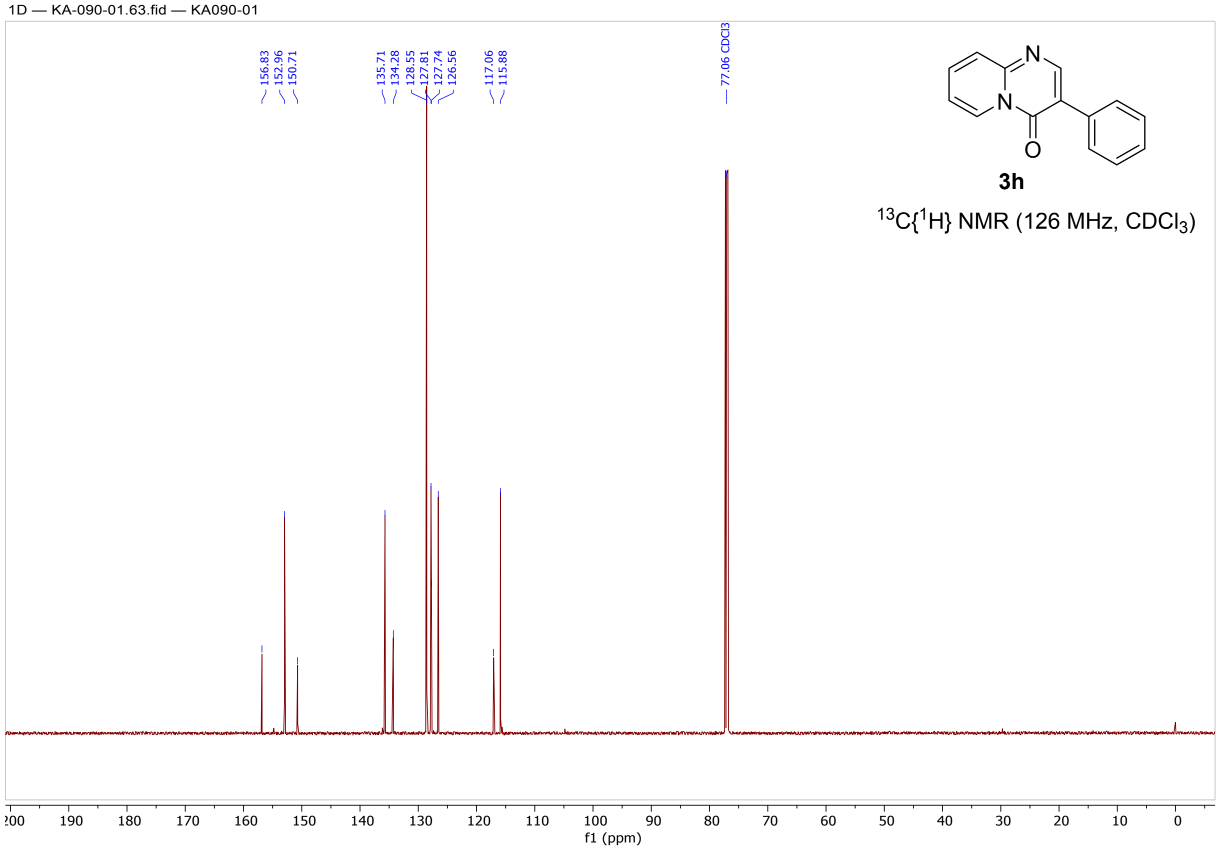
1D — KA-090-01.63.fid — KA090-01
156.83
152.96
150.71
135.71
134.28
128.55
127.81
127.74
126.56
117.06
115.88
77.06 CDCl3
N
N
O
3h
13C{1H} NMR (126 MHz, CDCl3)
f1 (ppm)

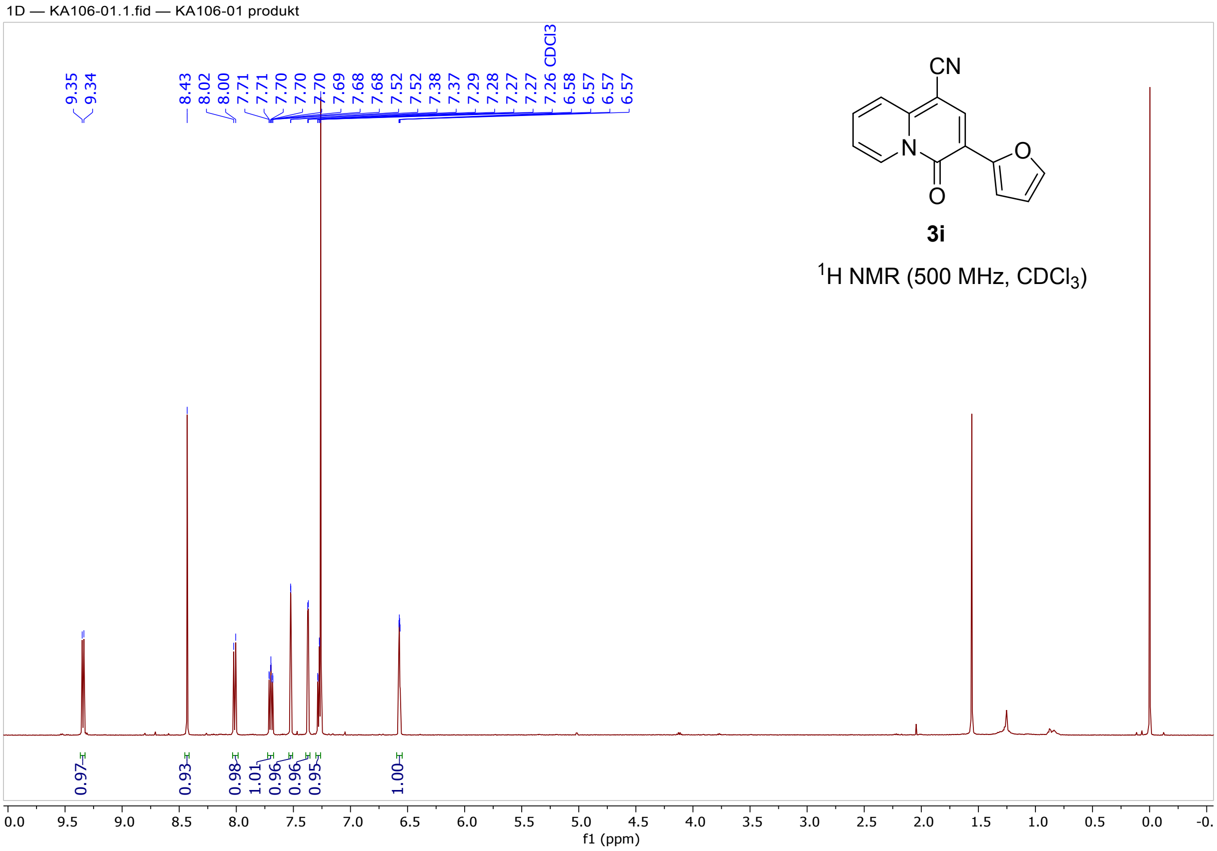
1D — KA106-01.1.fid — KA106-01 produkt
9.35 9.34
8.43 8.02 8.00 7.71 7.71 7.70 7.70 7.70 7.69 7.68 7.68 7.52 7.52 7.38 7.37 7.29 7.28 7.27 7.27 7.26 CDCl3 6.58 6.57 6.57 6.57
CN
N
O
O
3i
^{1}H NMR (500 MHz, $CDCl_3$)
0.97 0.93 0.98 1.01 0.96 0.96 0.95 1.00
f1 (ppm)

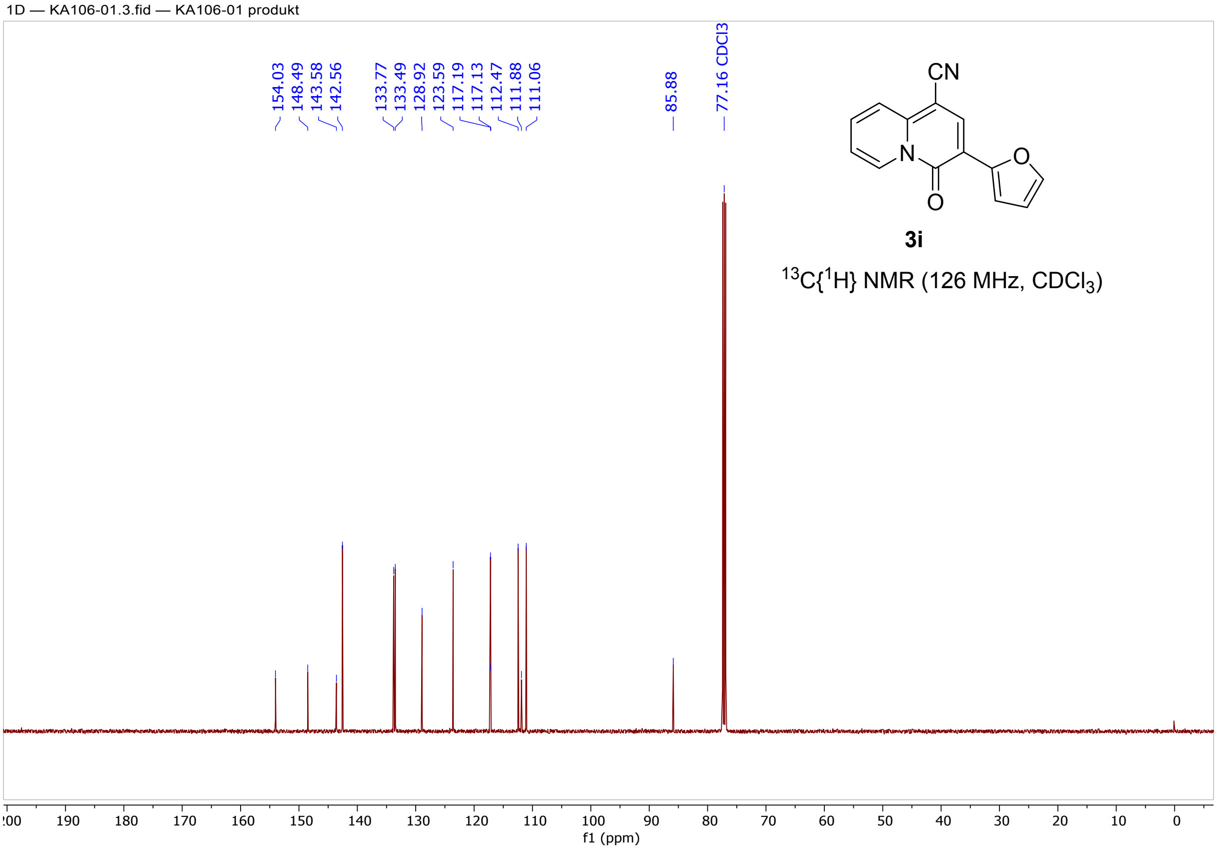
1D — KA106-01.3.fid — KA106-01 produkt
154.03 148.49 143.58 142.56
133.77 133.49 128.92 123.59 117.19 117.13 112.47 111.88 111.06
85.88
77.16 CDCl3
CN
N
O
O
3i
^{13}C{^{1}H} NMR (126 MHz, $CDCl_3$)
f1 (ppm)

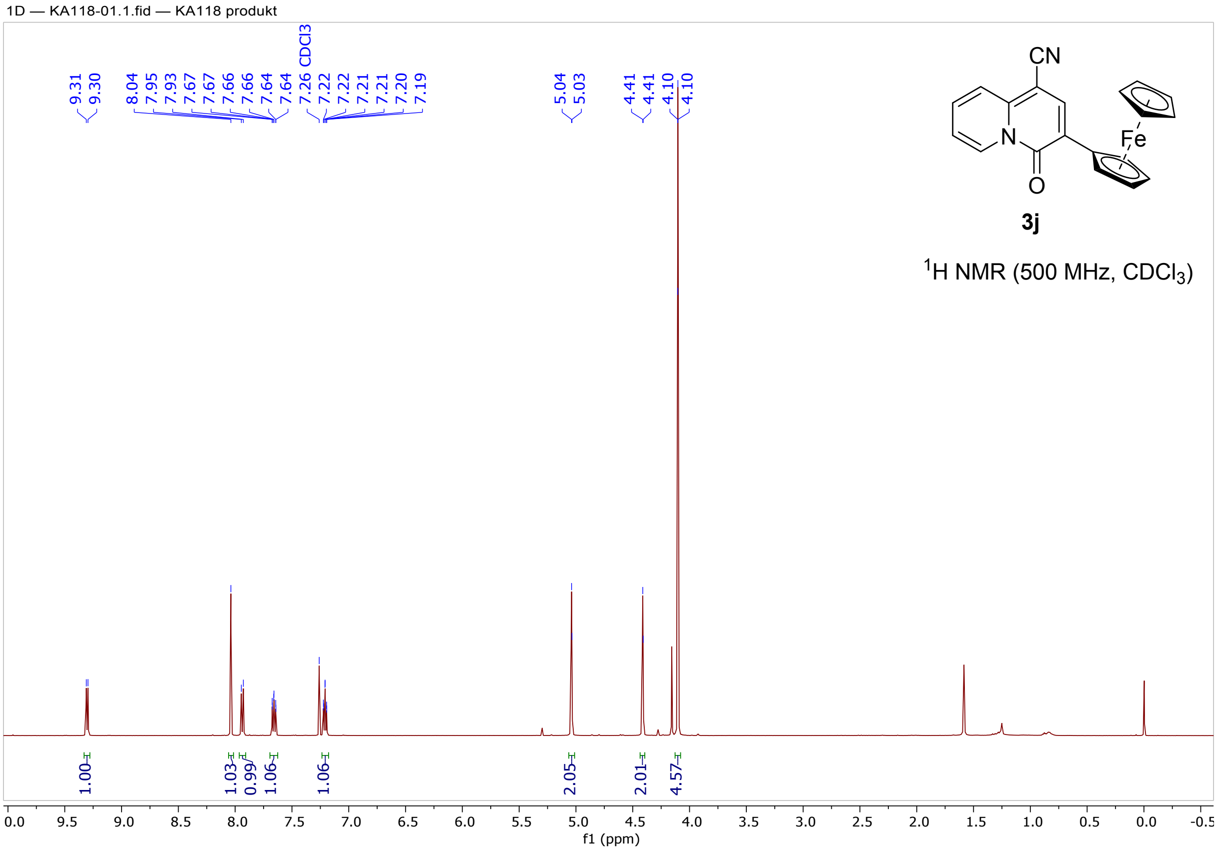
1D — KA118-01.1.fid — KA118 produkt
9.31
9.30
8.04
7.95
7.93
7.67
7.67
7.66
7.66
7.64
7.64
7.26 CDCl3
7.22
7.22
7.21
7.21
7.20
7.19
5.04
5.03
4.41
4.41
4.10
4.10
1.00
1.03
0.99
1.06
1.06
2.05
2.01
4.57
CN
N
O
Fe
3j
^{1}H NMR (500 MHz, $CDCl_3$)
f1 (ppm)

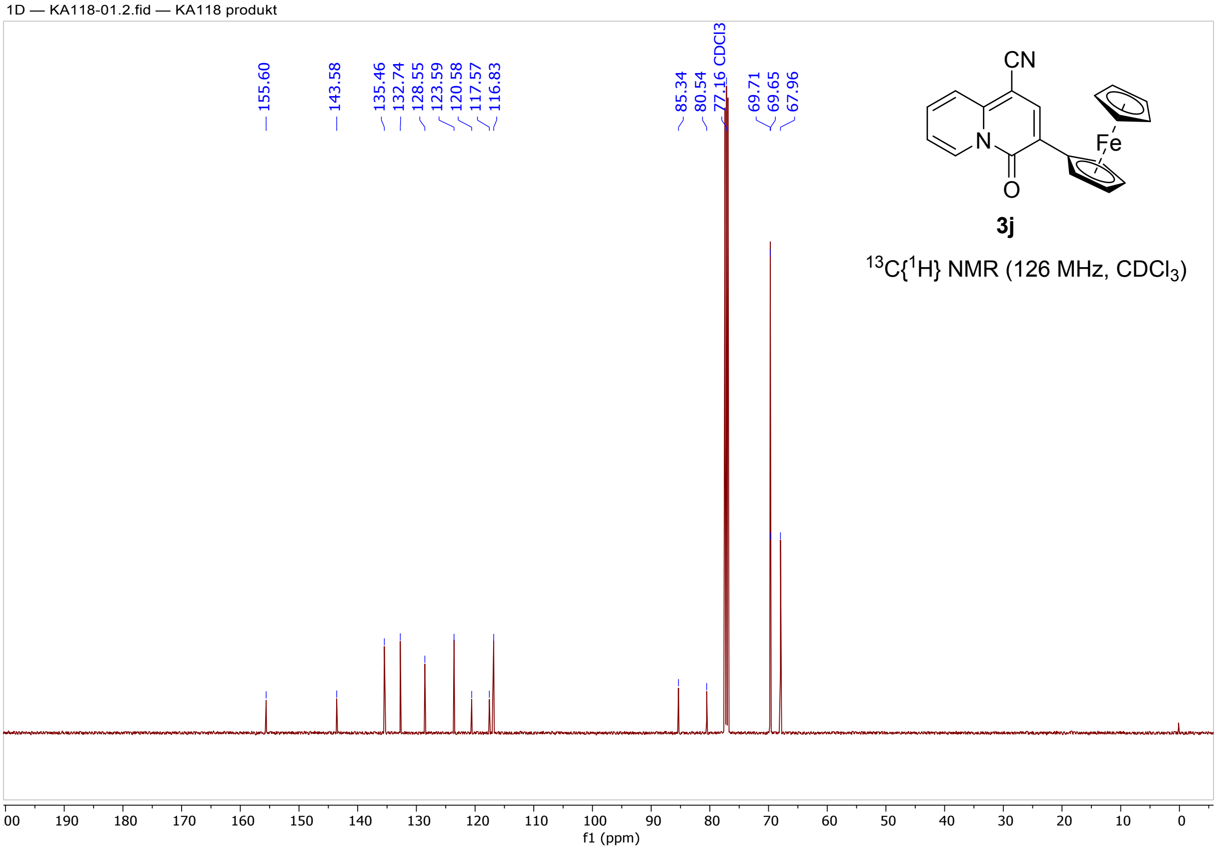
1D — KA118-01.2.fid — KA118 produkt
155.60
143.58
135.46
132.74
128.55
123.59
120.58
117.57
116.83
85.34
80.54
77.16 CDCl3
69.71
69.65
67.96
CN
N
O
Fe
3j
^{13}C{^{1}H} NMR (126 MHz, $CDCl_3$)
f1 (ppm)

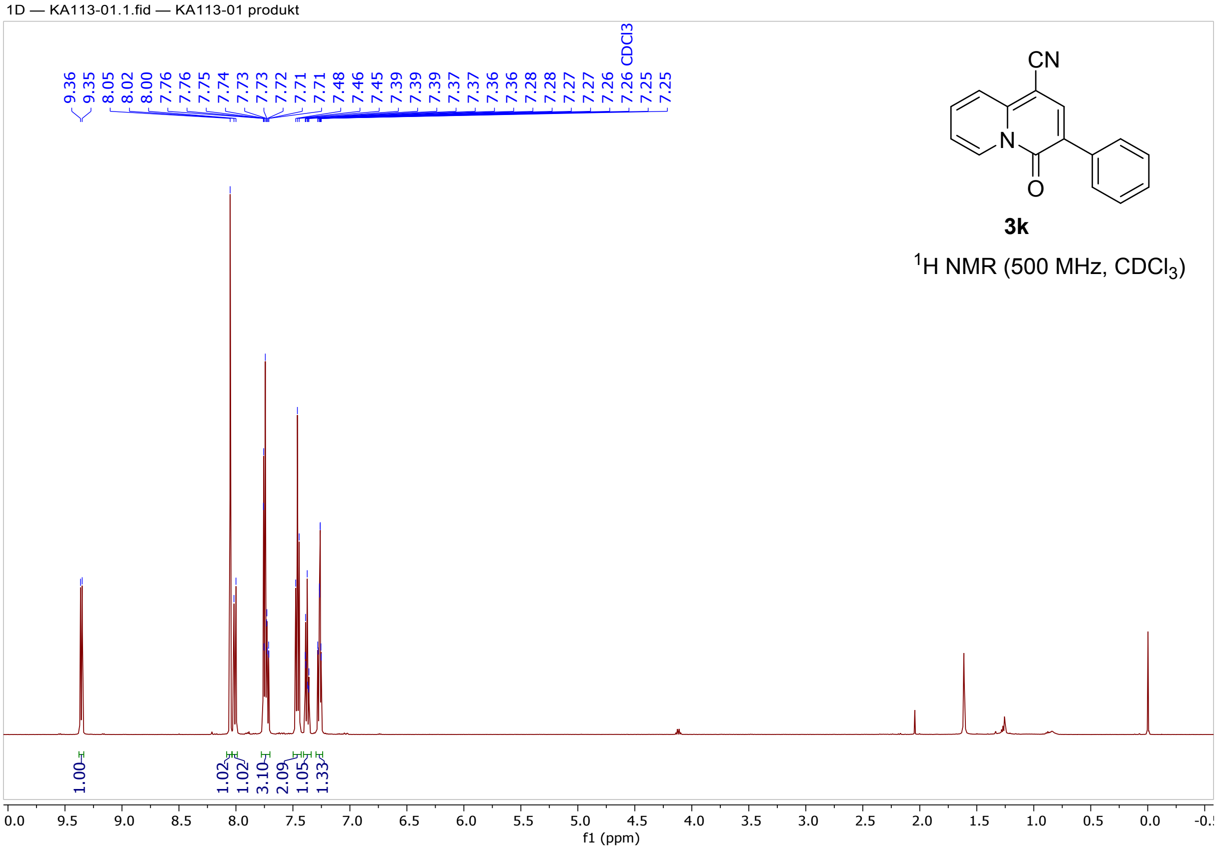
1D — KA113-01.1.fid — KA113-01 produkt
9.36 9.35 8.05 8.02 8.00 7.76 7.76 7.75 7.74 7.73 7.73 7.72 7.71 7.71 7.48 7.46 7.45 7.39 7.39 7.39 7.37 7.37 7.36 7.36 7.28 7.28 7.27 7.27 7.26 CDCl3 7.25 7.25
CN
N
O
3k
1H NMR (500 MHz, CDCl3)
1.00 1.02 1.02 3.10 2.09 1.05 1.33
f1 (ppm)

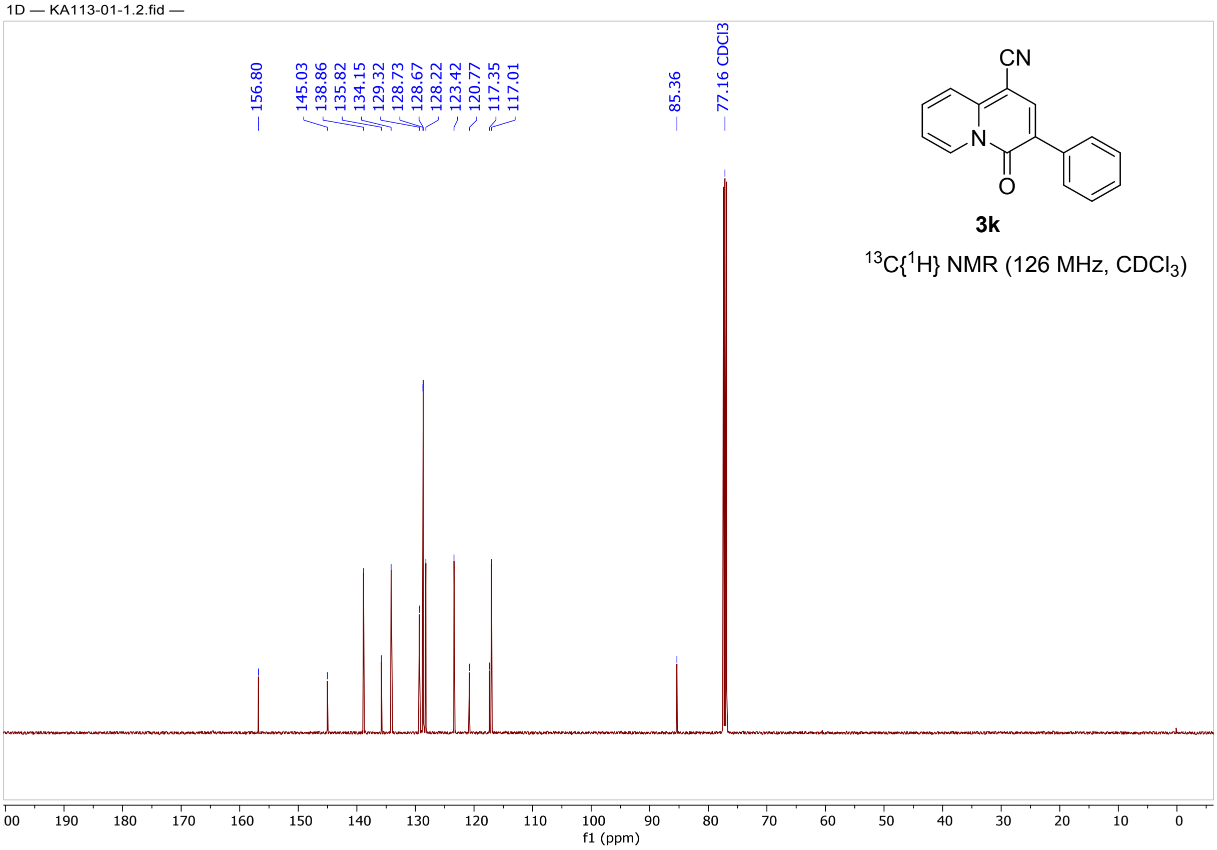
1D — KA113-01-1.2.fid —
156.80 145.03 138.86 135.82 134.15 129.32 128.73 128.67 128.22 123.42 120.77 117.35 117.01 85.36 77.16 CDCl3
CN
N
O
3k
13C{1H} NMR (126 MHz, CDCl3)
f1 (ppm)

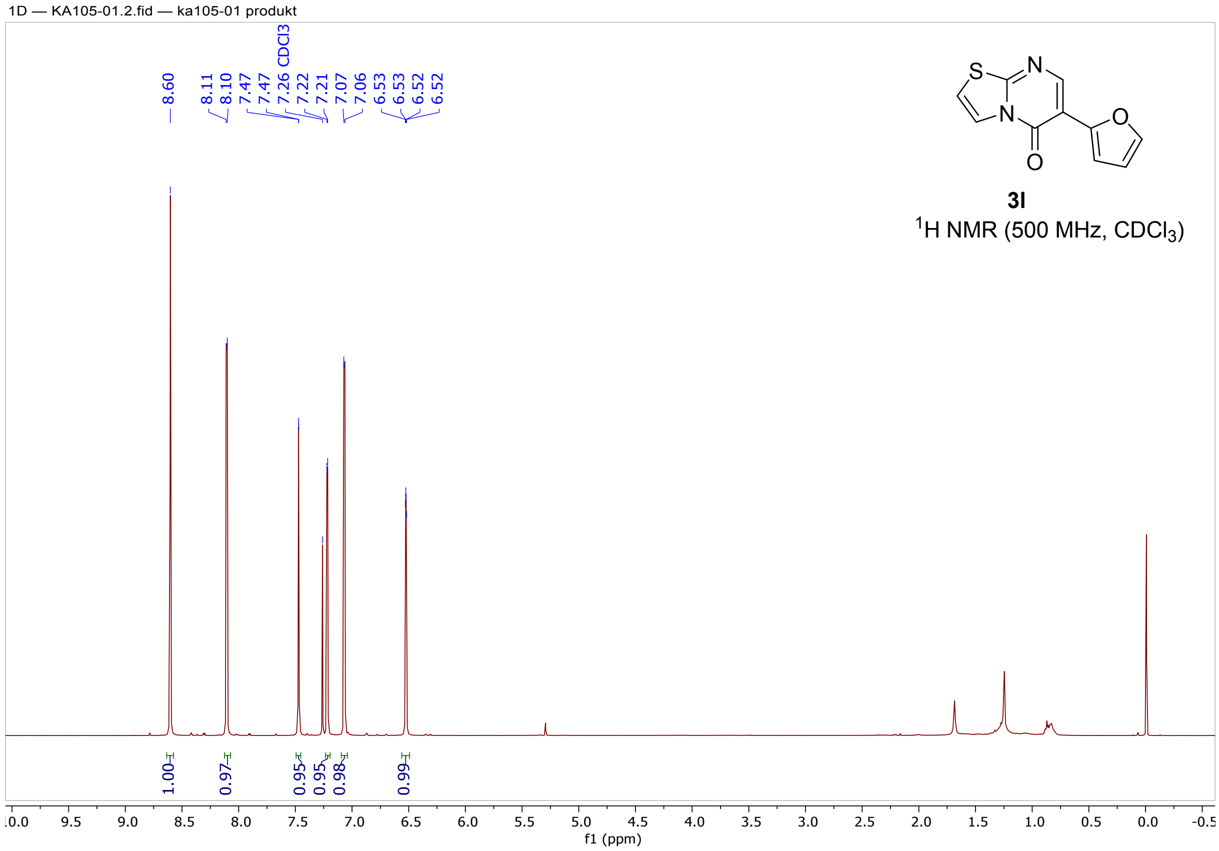
1D — KA105-01.2.fid — ka105-01 produkt
8.60
8.11
8.10
7.47
7.47
7.26 CDCl3
7.22
7.21
7.07
7.06
6.53
6.53
6.52
6.52
S
N
N
O
O
3l
1H NMR (500 MHz, CDCl3)
1.00
0.97
0.95
0.95
0.98
0.99
f1 (ppm)

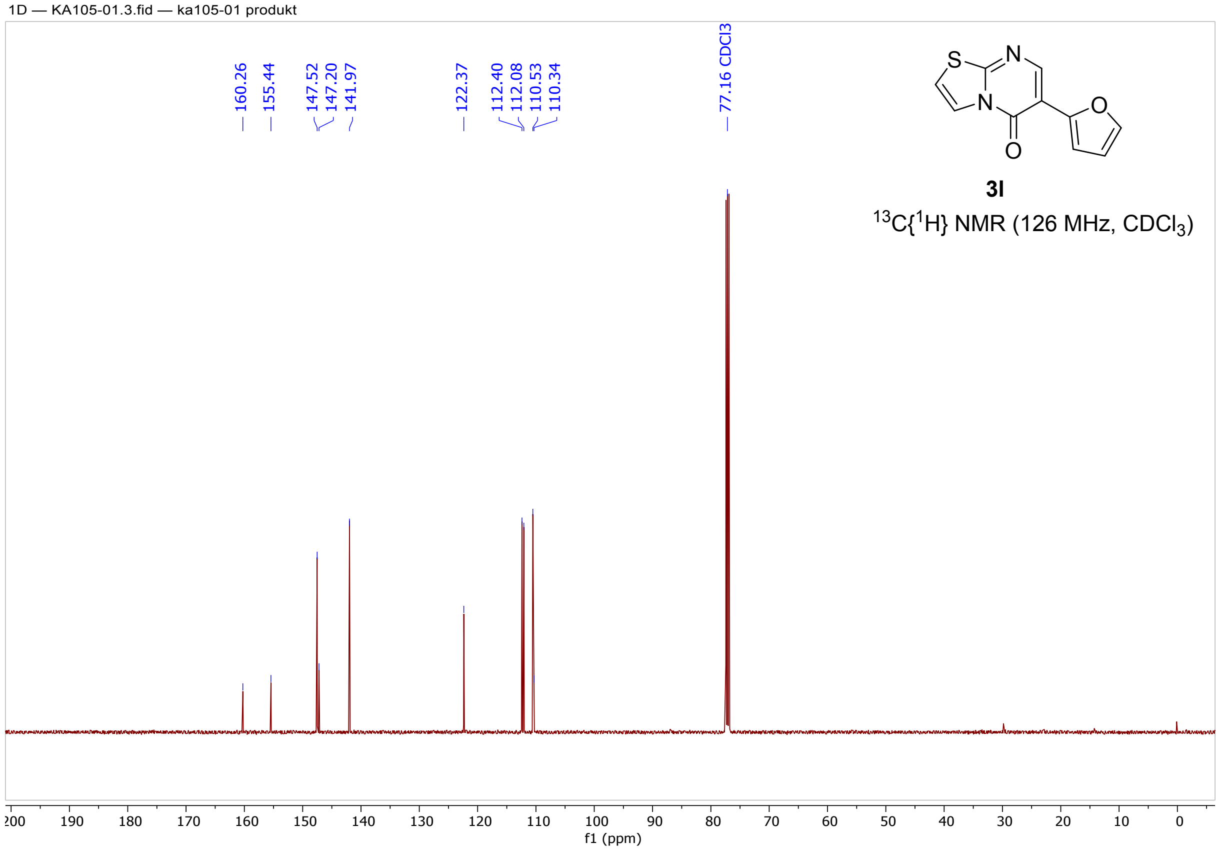
1D — KA105-01.3.fid — ka105-01 produkt
160.26
155.44
147.52
147.20
141.97
122.37
112.40
112.08
110.53
110.34
77.16 CDCl3
S
N
N
O
O
3l
13C{1H} NMR (126 MHz, CDCl3)
f1 (ppm)

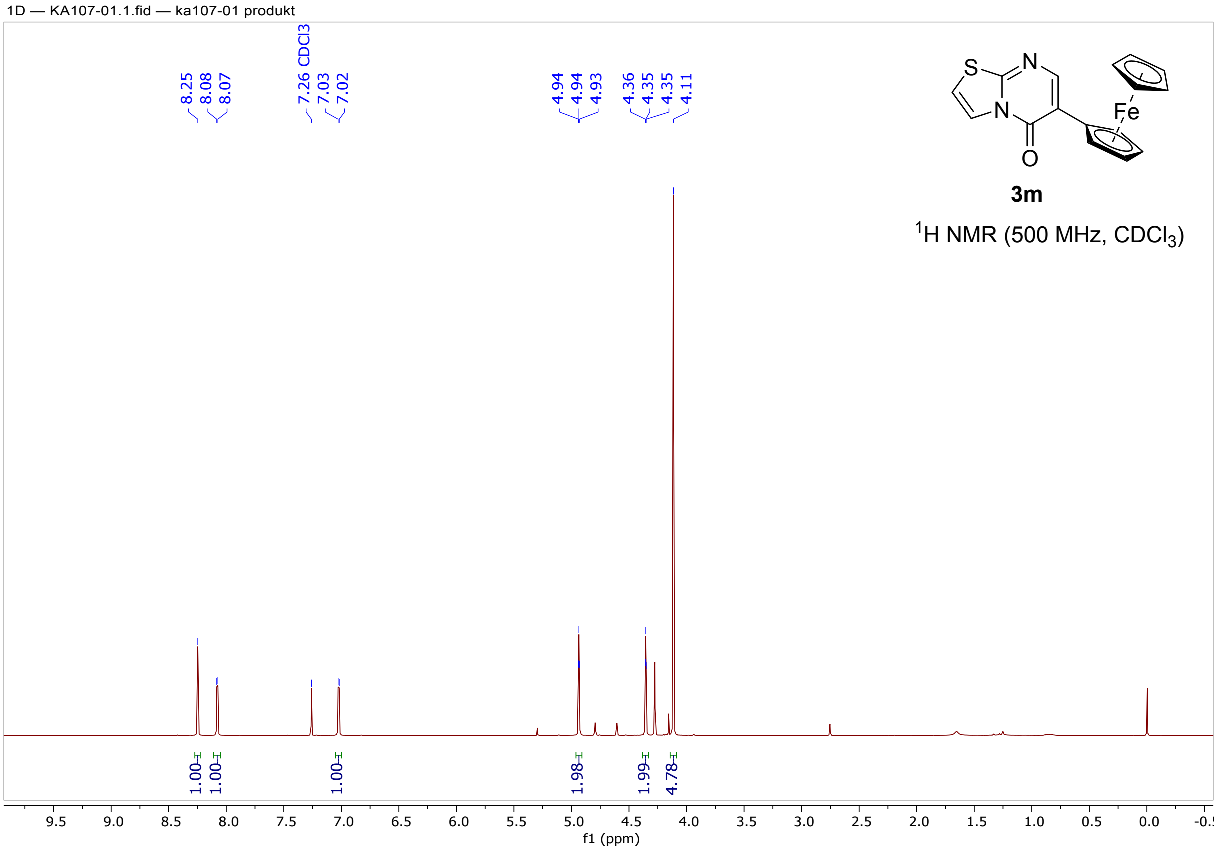
1D — KA107-01.1.fid — ka107-01 produkt
8.25
8.08
8.07
7.26 CDCl3
7.03
7.02
4.94
4.94
4.93
4.36
4.35
4.35
4.11
S
N
N
O
Fe
3m
^{1}H NMR (500 MHz, CDCl$_3$)
1.00
1.00
1.00
1.98
1.99
4.78
9.5 9.0 8.5 8.0 7.5 7.0 6.5 6.0 5.5 5.0 4.5 4.0 3.5 3.0 2.5 2.0 1.5 1.0 0.5 0.0
f1 (ppm)

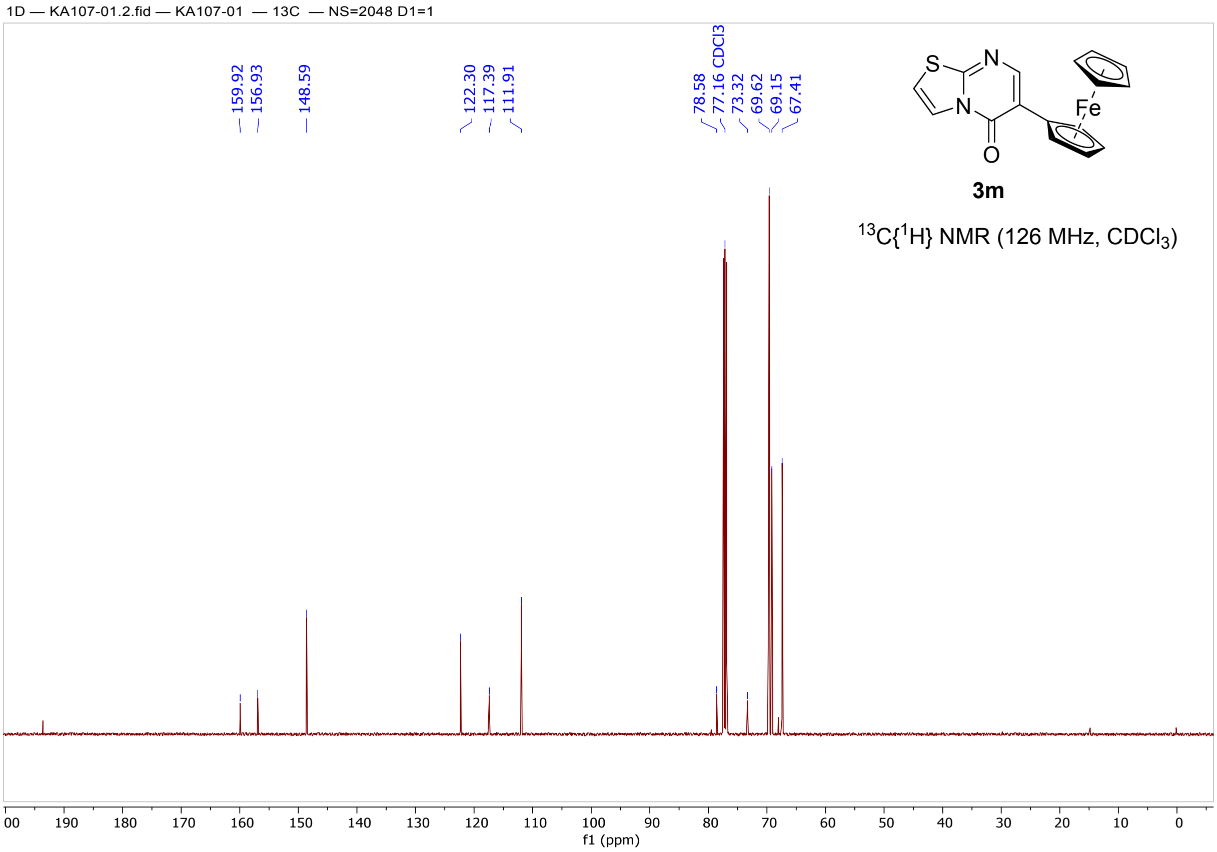
1D — KA107-01.2.fid — KA107-01 — 13C — NS=2048 D1=1
159.92
156.93
148.59
122.30
117.39
111.91
78.58
77.16 CDCl3
73.32
69.62
69.15
67.41
S
N
N
O
Fe
3m
^{13}C{^{1}H} NMR (126 MHz, CDCl$_3$)
190 180 170 160 150 140 130 120 110 100 90 80 70 60 50 40 30 20 10 0
f1 (ppm)

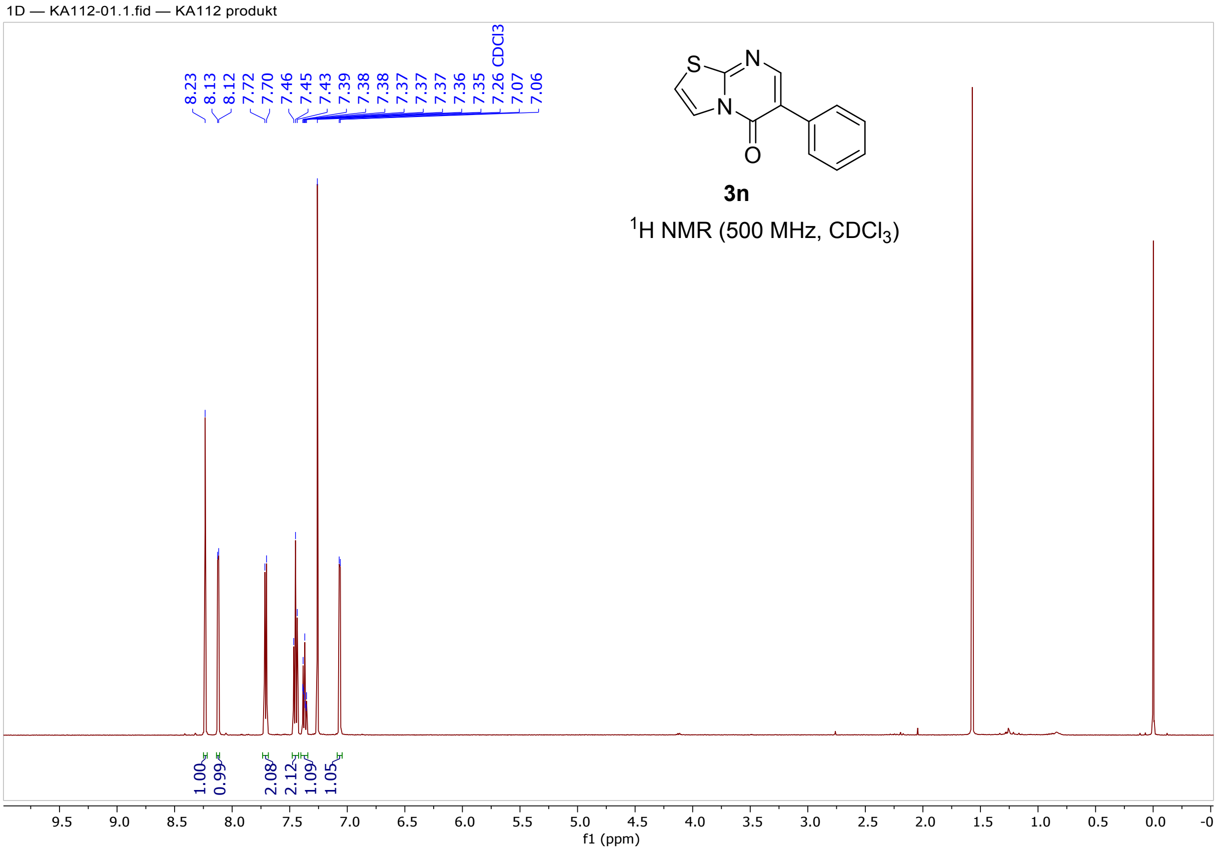
1D — KA112-01.1.fid — KA112 produkt
8.23
8.13
8.12
7.72
7.70
7.46
7.45
7.43
7.39
7.38
7.38
7.37
7.37
7.37
7.36
7.35
7.26 CDCl3
7.07
7.06
3n
¹H NMR (500 MHz, CDCl3)
1.00
0.99
2.08
2.12
1.09
1.05
f1 (ppm)

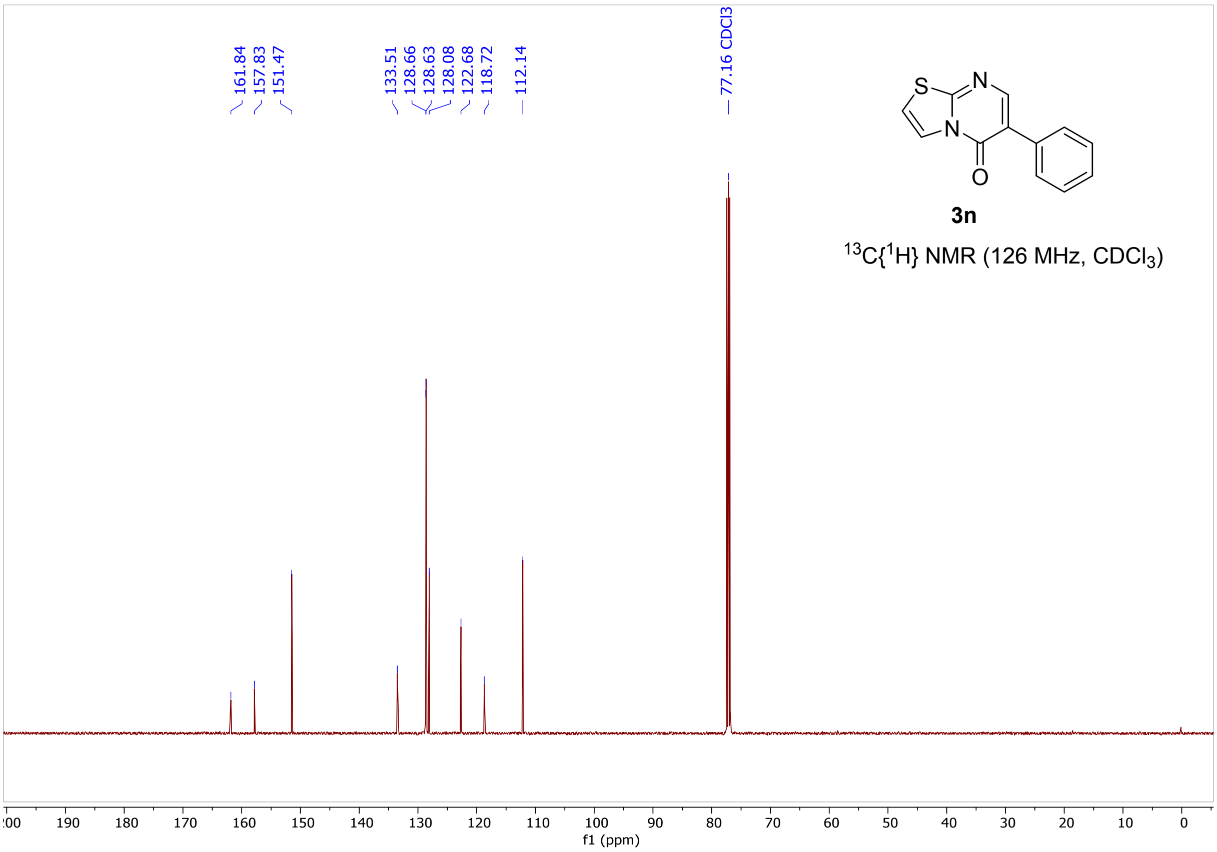
161.84
157.83
151.47
133.51
128.66
128.63
128.08
122.68
118.72
112.14
77.16 CDCl3
3n
¹³C{¹H} NMR (126 MHz, CDCl3)
f1 (ppm)

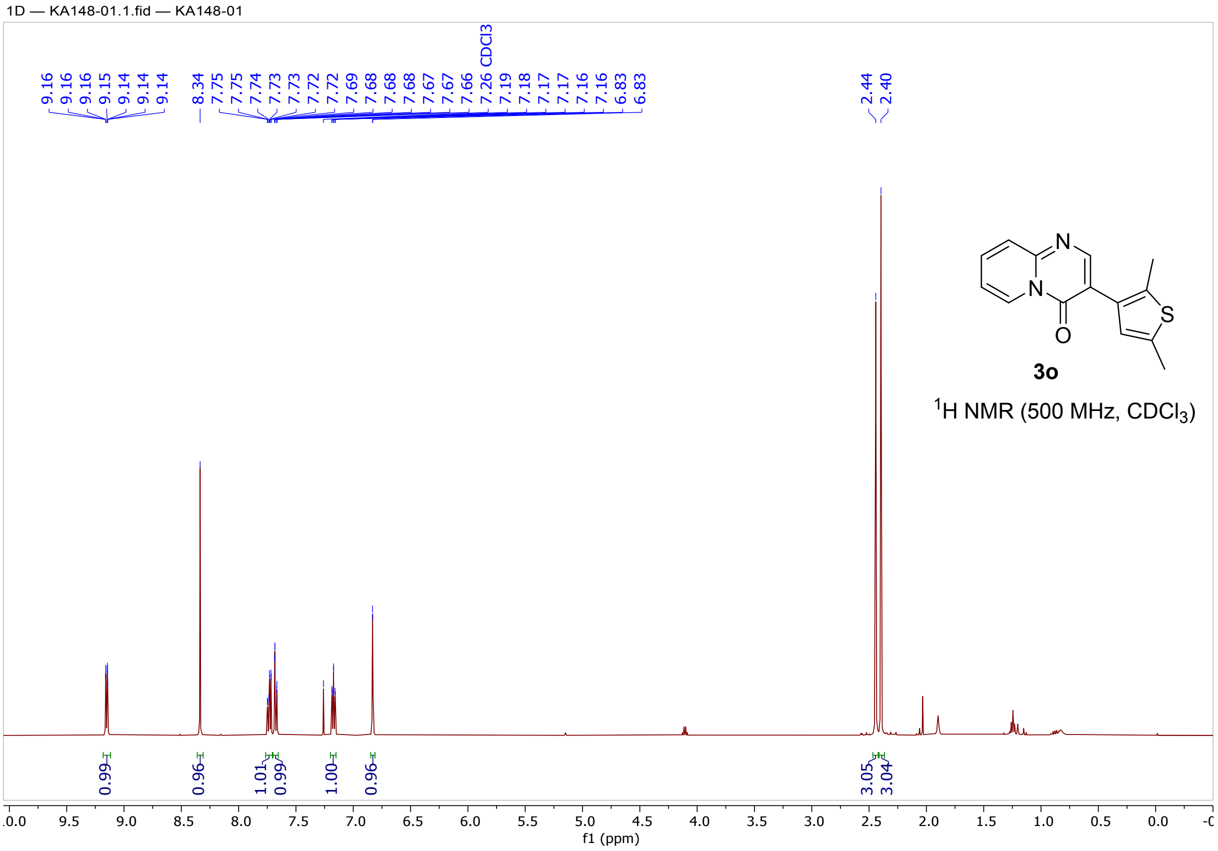
1D — KA148-01.1.fid — KA148-01
9.16 9.16 9.15 9.14 9.14 9.14 8.34 7.75 7.75 7.74 7.73 7.73 7.72 7.72 7.69 7.68 7.68 7.68 7.67 7.67 7.66 7.26 CDCl3 7.19 7.18 7.17 7.17 7.16 7.16 6.83 6.83 2.44 2.40
3o
1H NMR (500 MHz, CDCl3)
0.99 0.96 1.01 0.99 1.00 0.96 3.05 3.04
f1 (ppm)

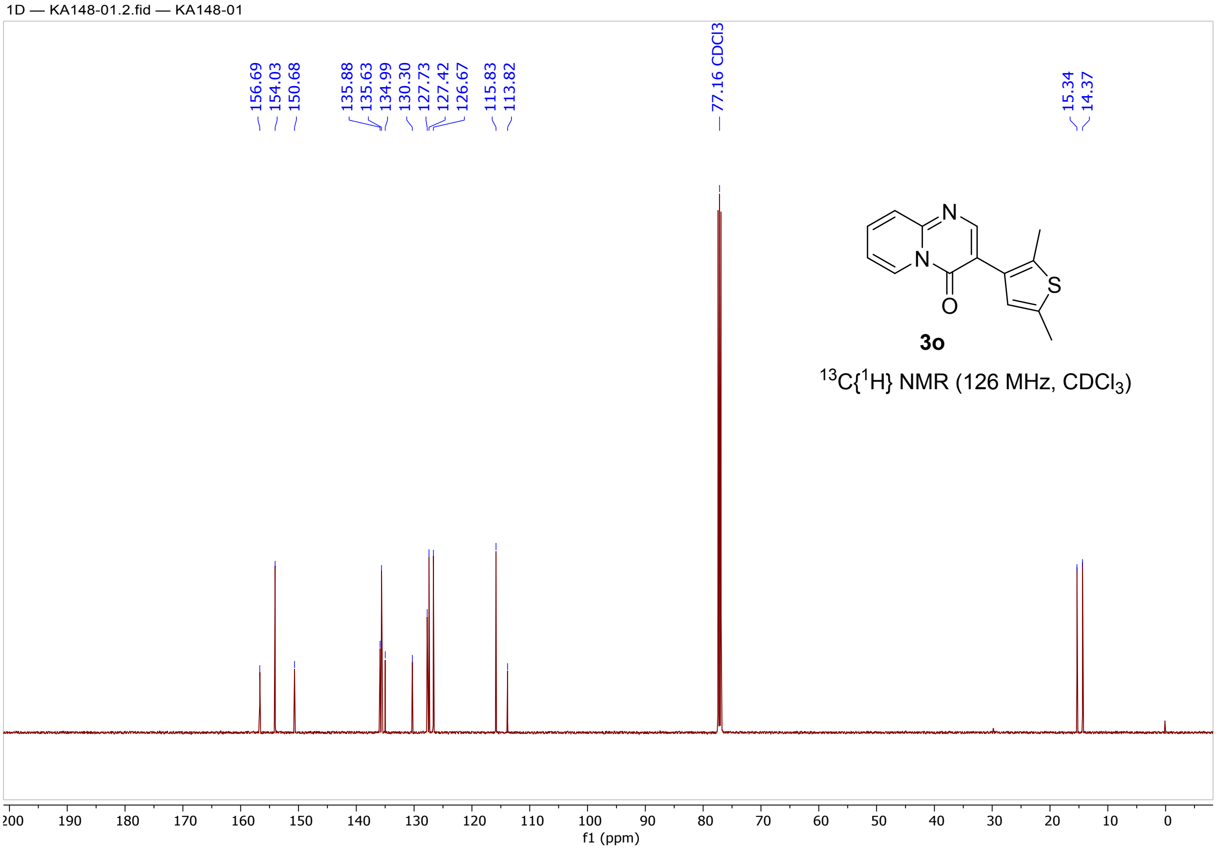
1D — KA148-01.2.fid — KA148-01
156.69 154.03 150.68 135.88 135.63 134.99 130.30 127.73 127.42 126.67 115.83 113.82 77.16 CDCl3 15.34 14.37
3o
13C{1H} NMR (126 MHz, CDCl3)
f1 (ppm)

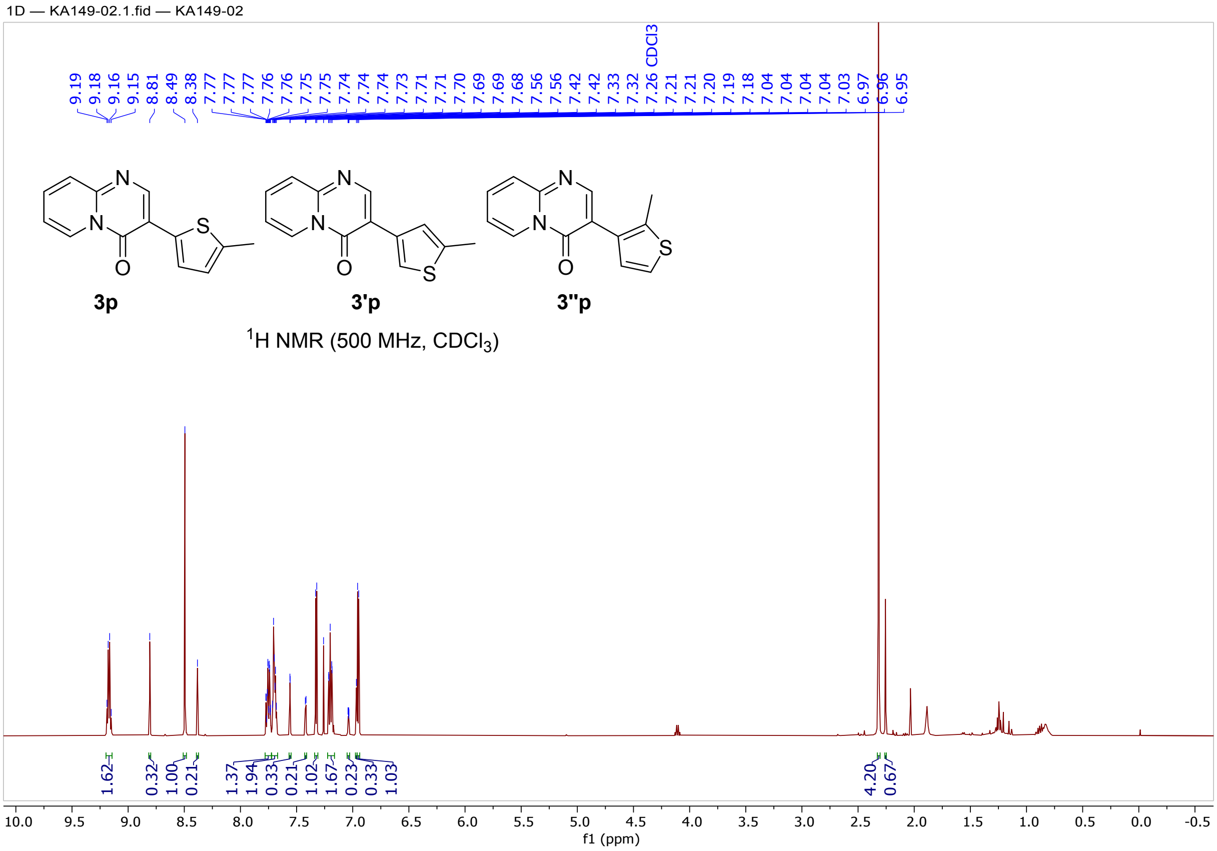
1D — KA149-02.1.fid — KA149-02
9.19 9.18 9.16 9.15 8.81 8.49 8.38 7.77 7.77 7.76 7.76 7.75 7.75 7.74 7.74 7.74 7.73 7.71 7.71 7.70 7.69 7.69 7.68 7.56 7.56 7.42 7.42 7.33 7.32 7.26 CDCl3 7.21 7.21 7.20 7.19 7.18 7.04 7.04 7.04 7.03 6.97 6.96 6.95
3p
3'p
3''p
1H NMR (500 MHz, CDCl3)
1.62 0.32 1.00 0.21 1.37 1.94 0.33 0.21 1.02 1.67 0.23 0.33 1.03 4.20 0.67
f1 (ppm)

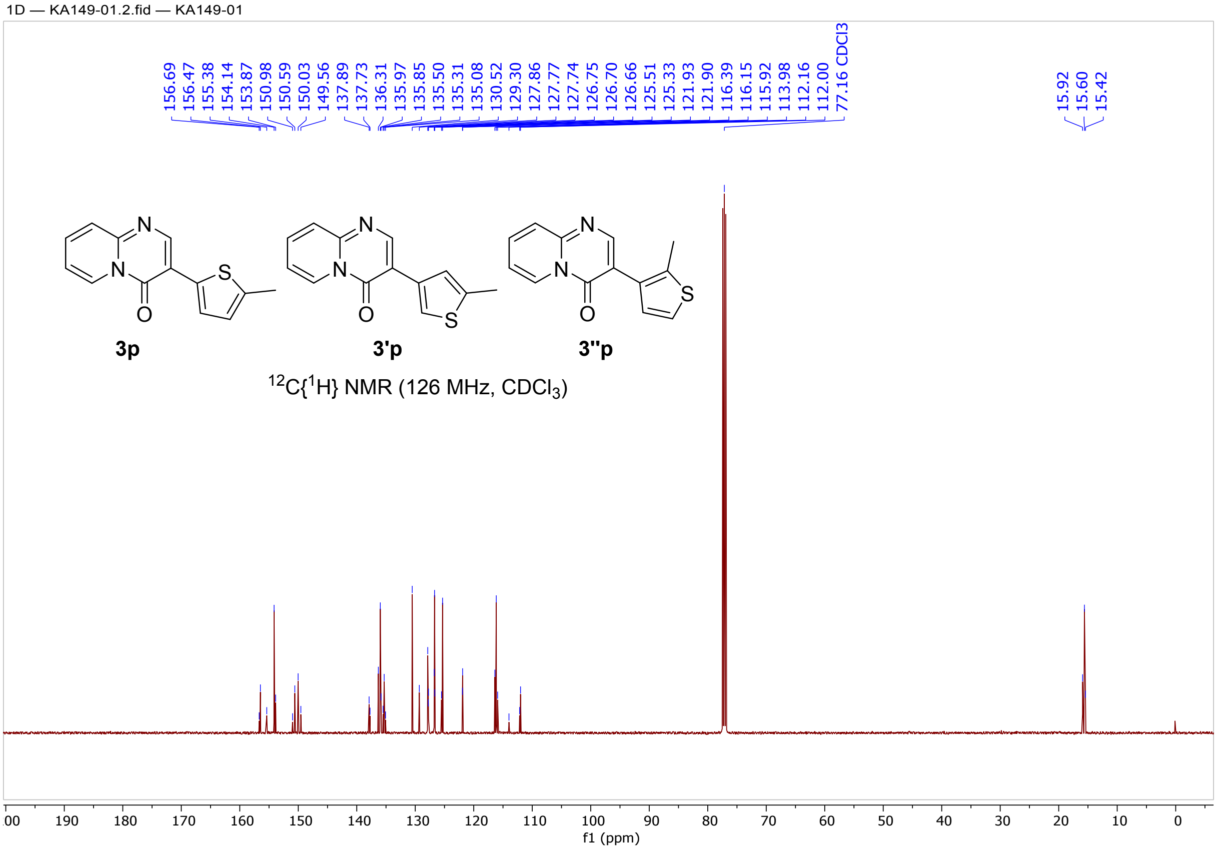
1D — KA149-01.2.fid — KA149-01
156.69 156.47 155.38 154.14 153.87 150.98 150.59 150.03 149.56 137.89 137.73 136.31 135.97 135.85 135.50 135.31 135.08 130.52 129.30 127.86 127.74 126.75 126.70 126.66 125.51 125.33 121.93 121.90 116.39 116.15 115.92 113.98 112.16 112.00 77.16 CDCl3
15.92 15.60 15.42
3p
3'p
3''p
12C{1H} NMR (126 MHz, CDCl3)
f1 (ppm)

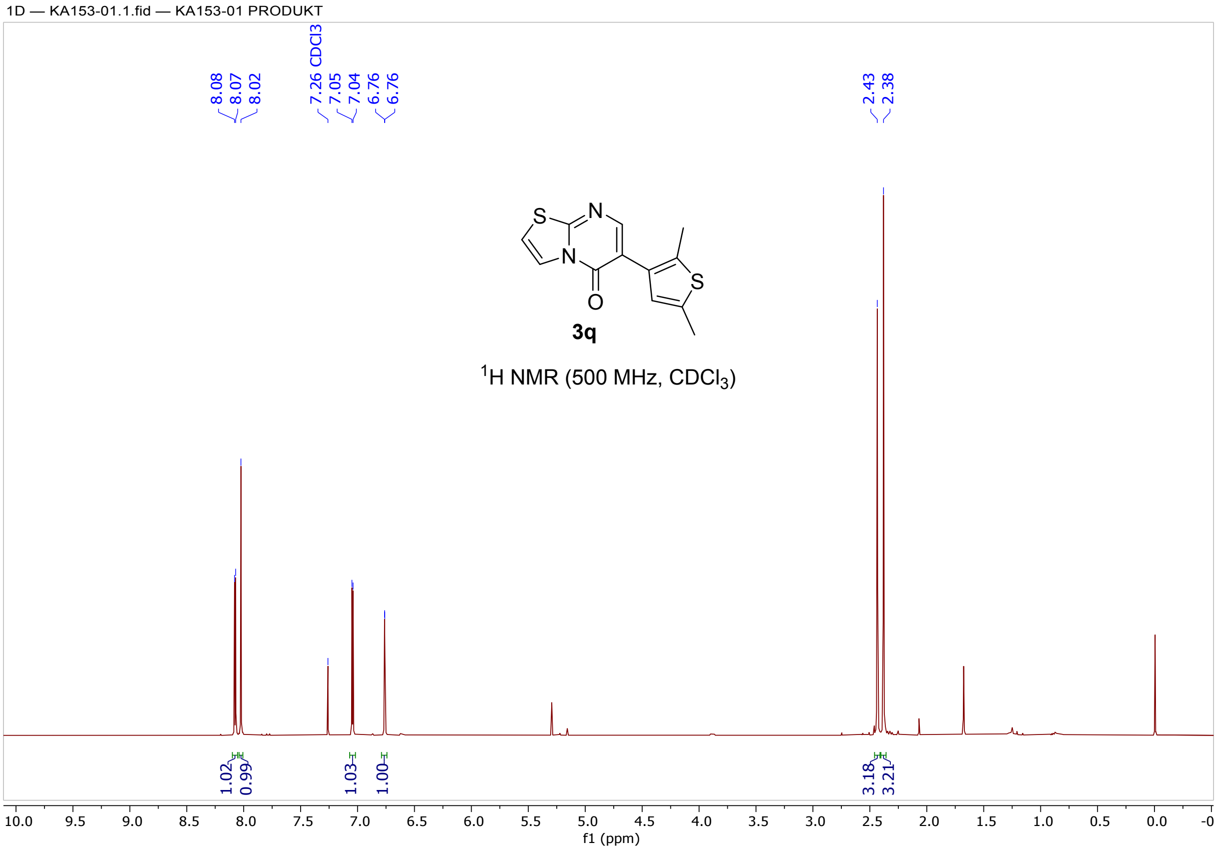
1D — KA153-01.1.fid — KA153-01 PRODUKT
8.08
8.07
8.02
7.26 CDCl3
7.05
7.04
6.76
6.76
2.43
2.38
3q
1H NMR (500 MHz, CDCl3)
1.02
0.99
1.03
1.00
3.18
3.21
f1 (ppm)

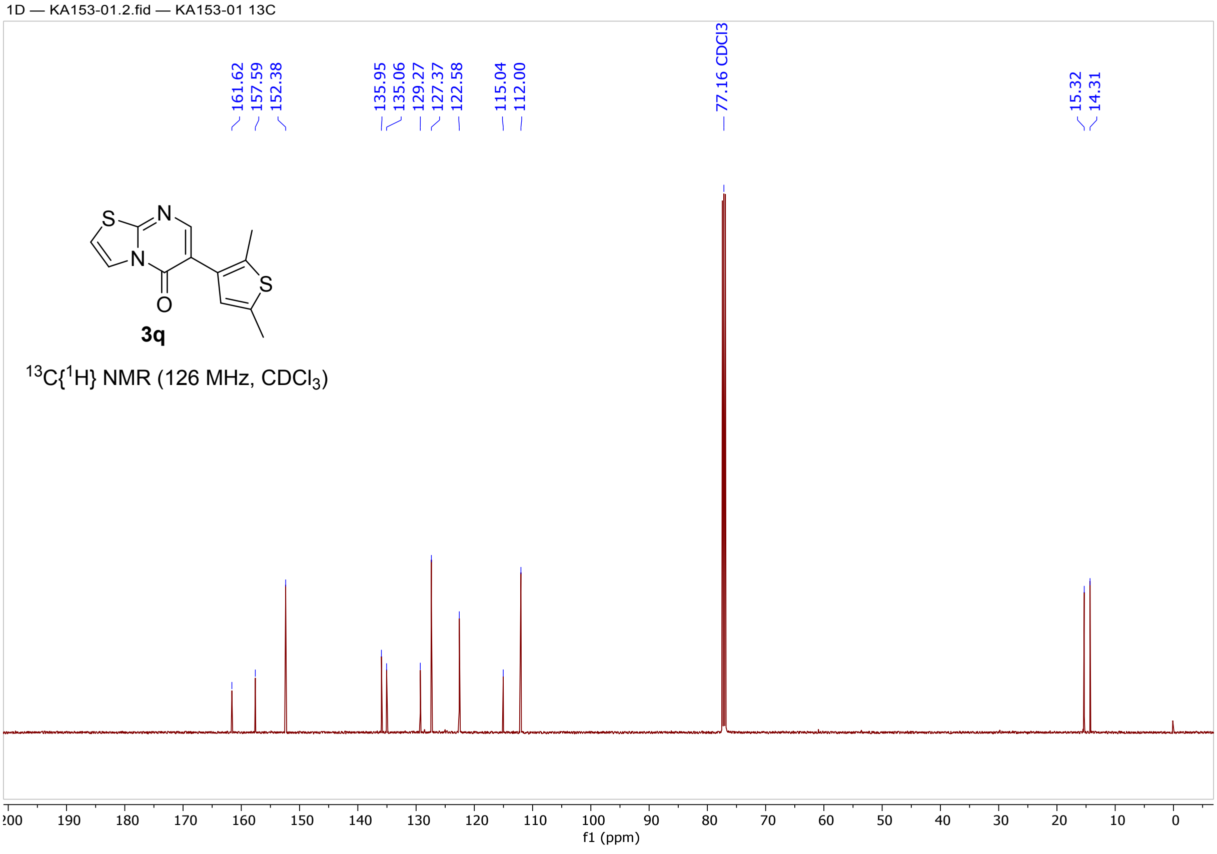
1D — KA153-01.2.fid — KA153-01 13C
161.62
157.59
152.38
135.95
135.06
129.27
127.37
122.58
115.04
112.00
77.16 CDCl3
15.32
14.31
3q
13C{1H} NMR (126 MHz, CDCl3)
f1 (ppm)

1D — KA152-01.1.fid — KA152 FR 2-7

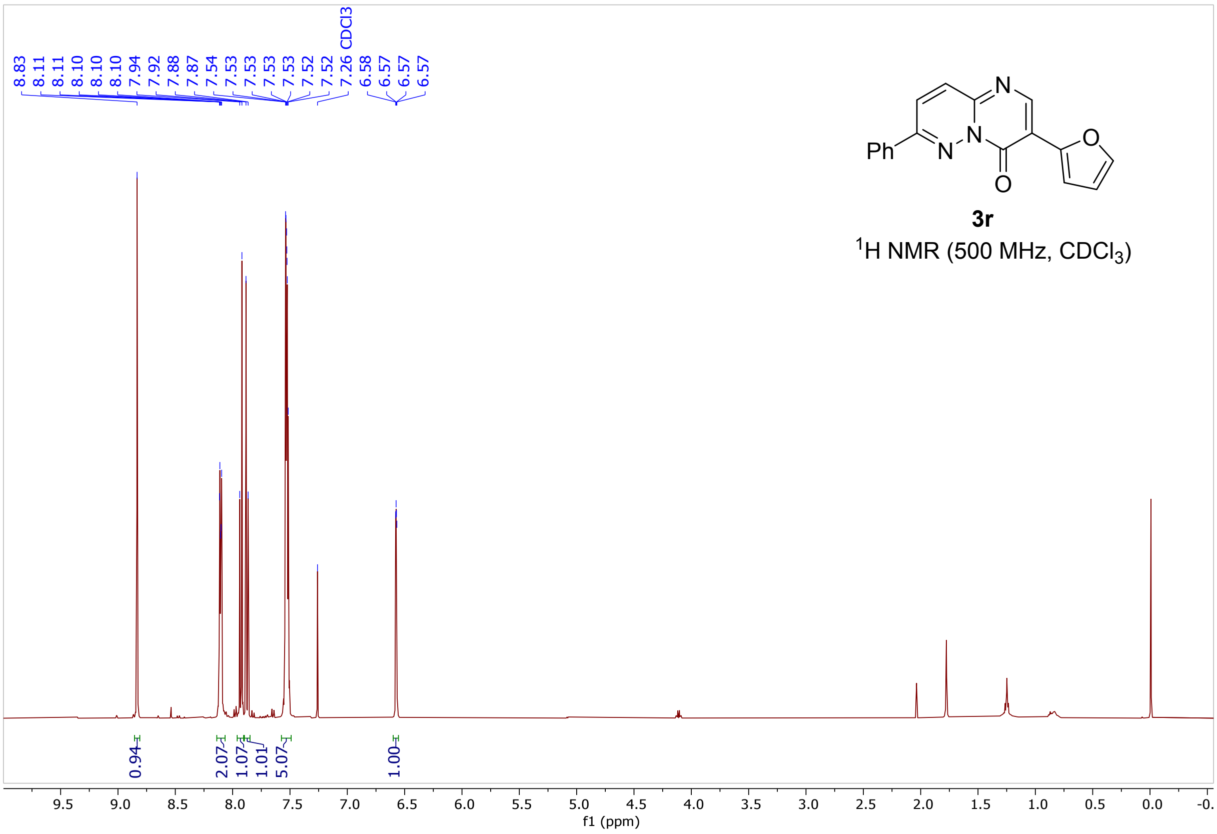

3r

^{1}H NMR (500 MHz, $CDCl_3$)

1D — KA152-01.2.fid — KA152-01

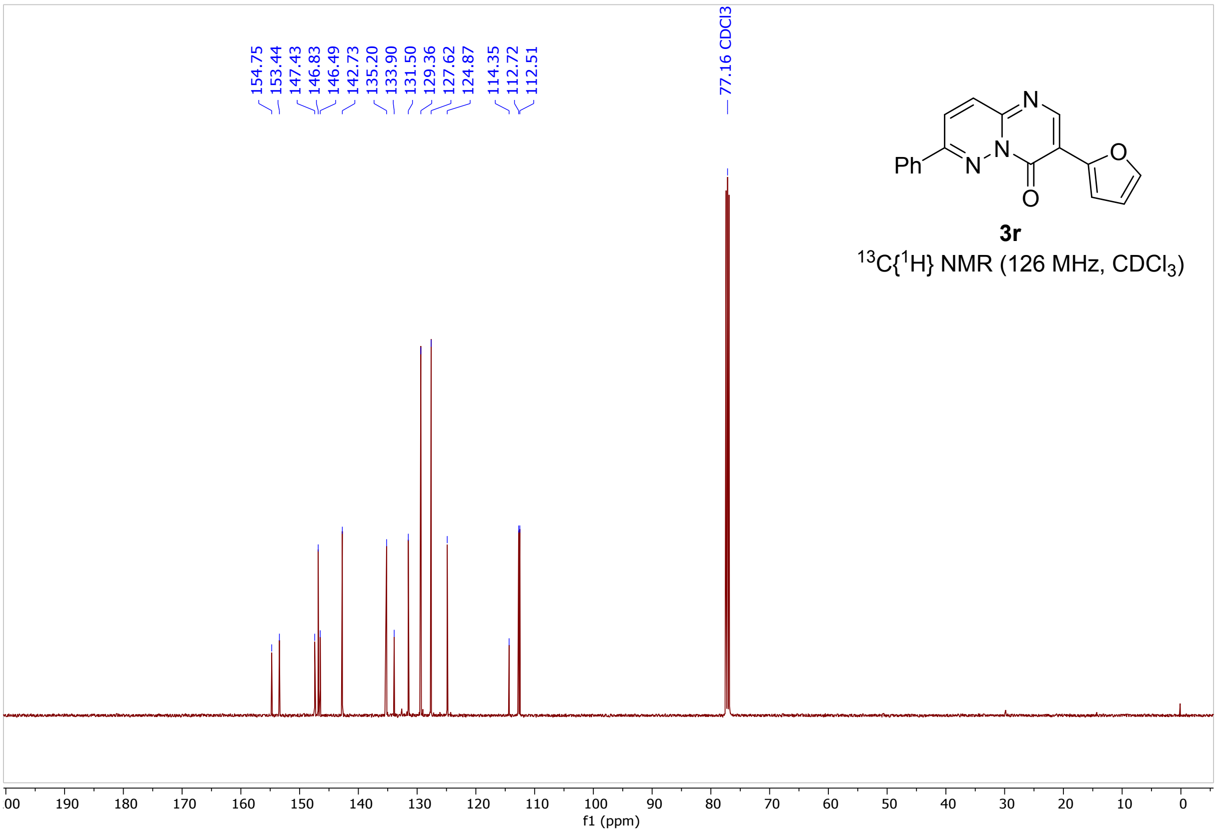

3r

^{13}C{^{1}H} NMR (126 MHz, $CDCl_3$)

3. Copies of IR spectra.

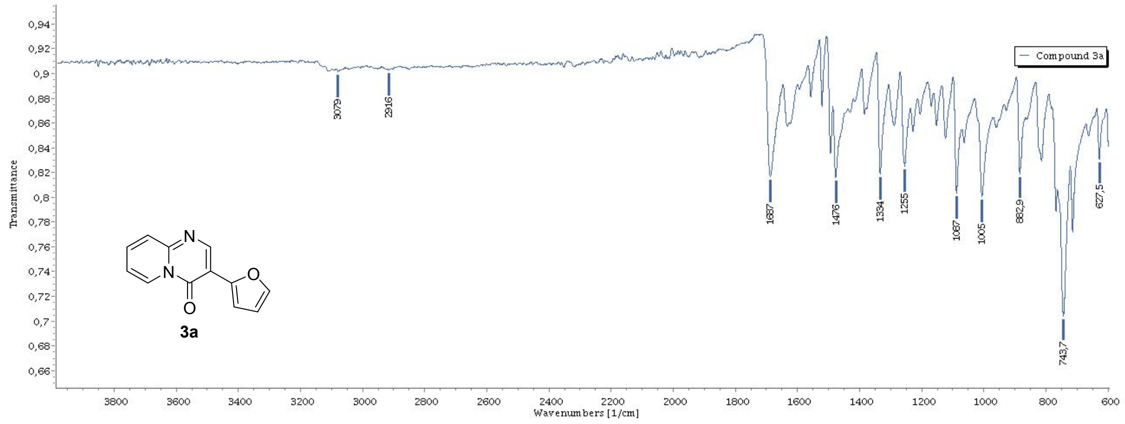

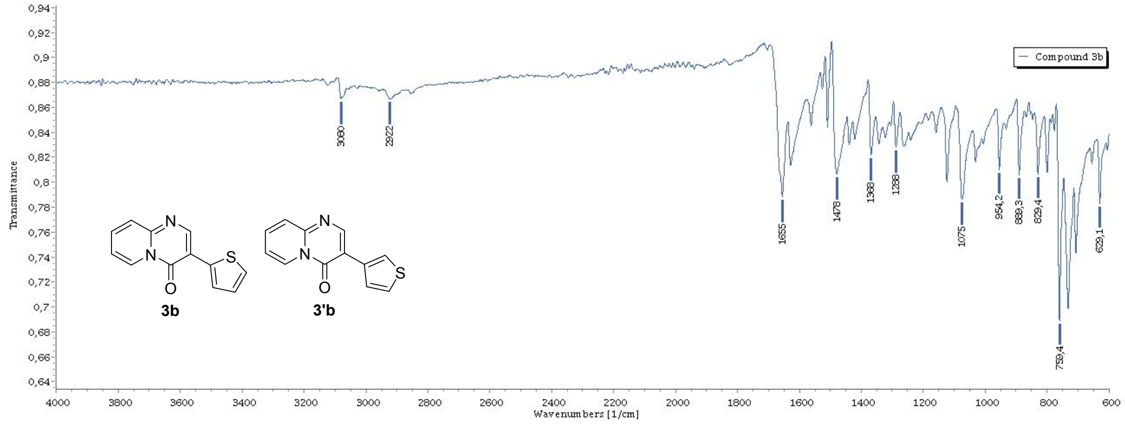

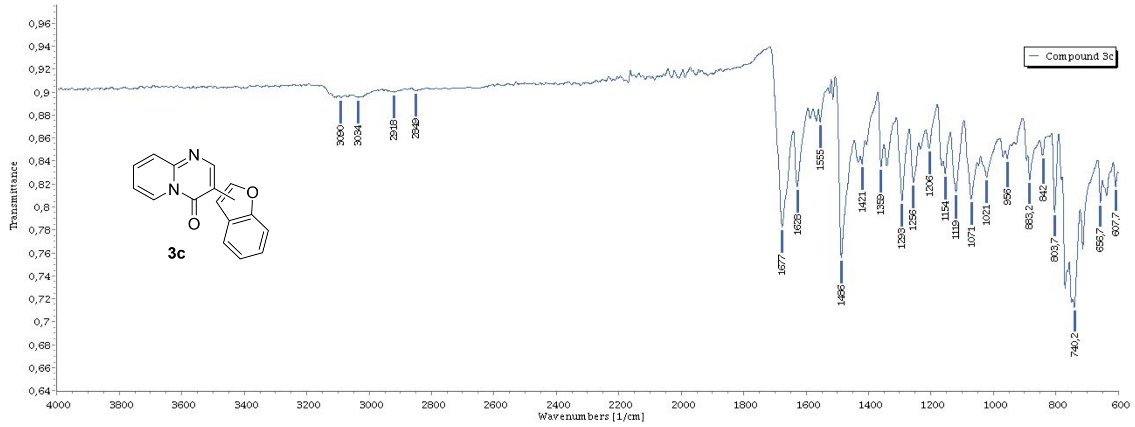

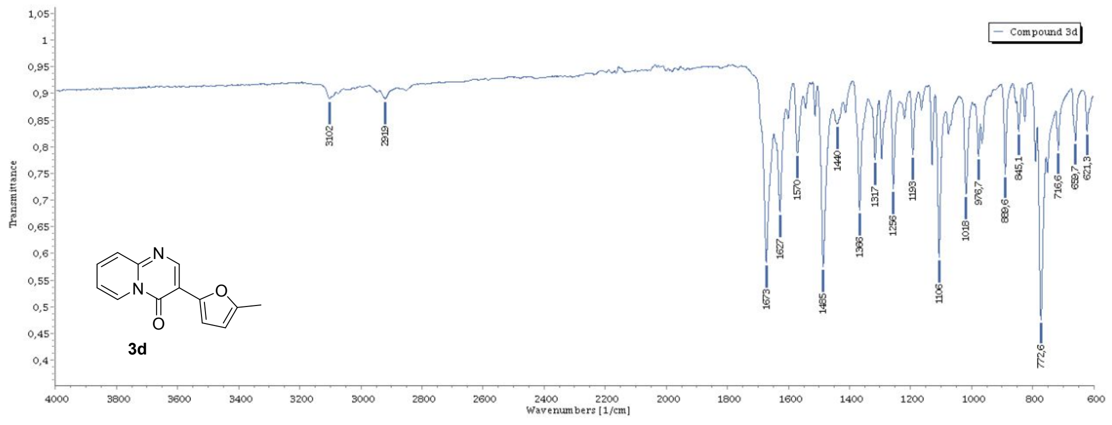

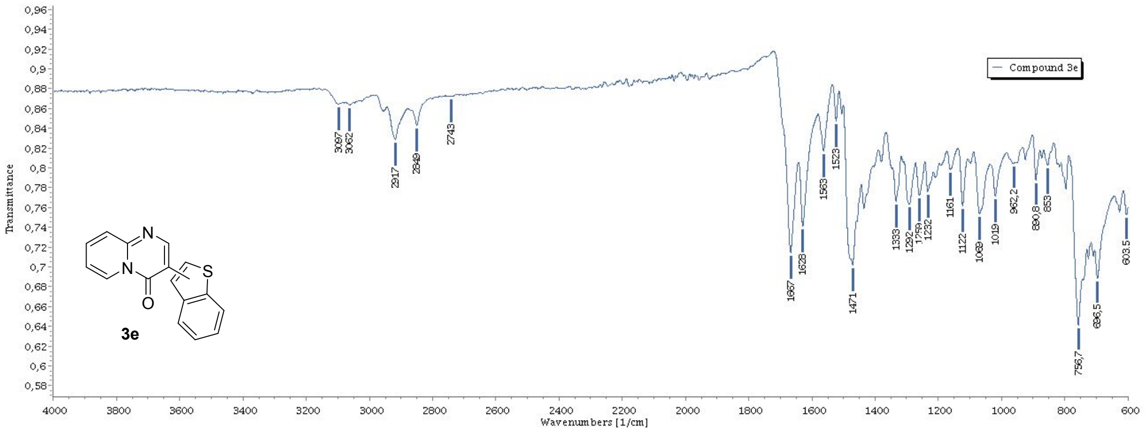
Compound 3e
Transmittance
Wavenumbers [1/cm]
3097
3062
2917
2849
2743
1667
1628
1563
1523
1471
1333
1292
1269
1232
1161
1122
1069
1019
962,2
890,8
853
756,7
696,5
603,5
3e

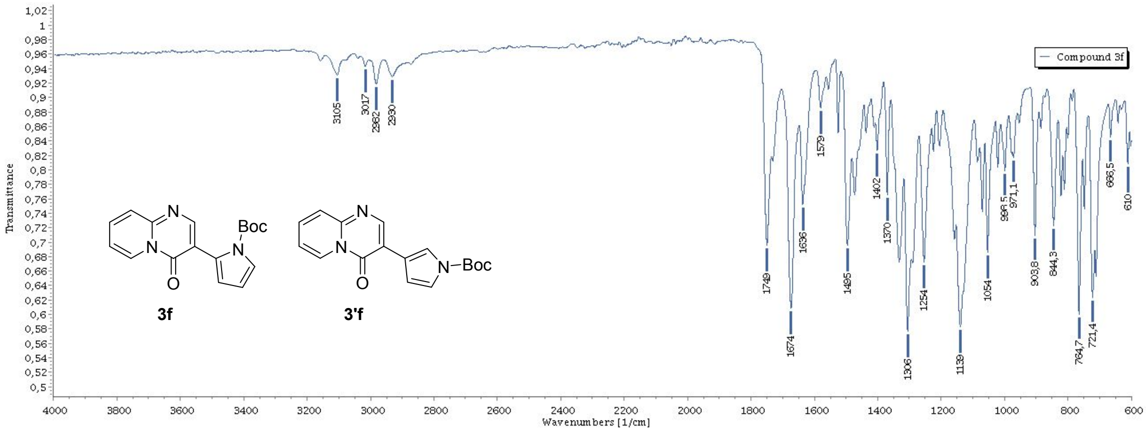
Compound 3f
Transmittance
Wavenumbers [1/cm]
3105
3017
2982
2930
1749
1674
1636
1579
1495
1402
1370
1306
1254
1139
1054
996,5
971,1
903,8
844,3
764,7
721,4
666,5
610
Boc
3f
3'f

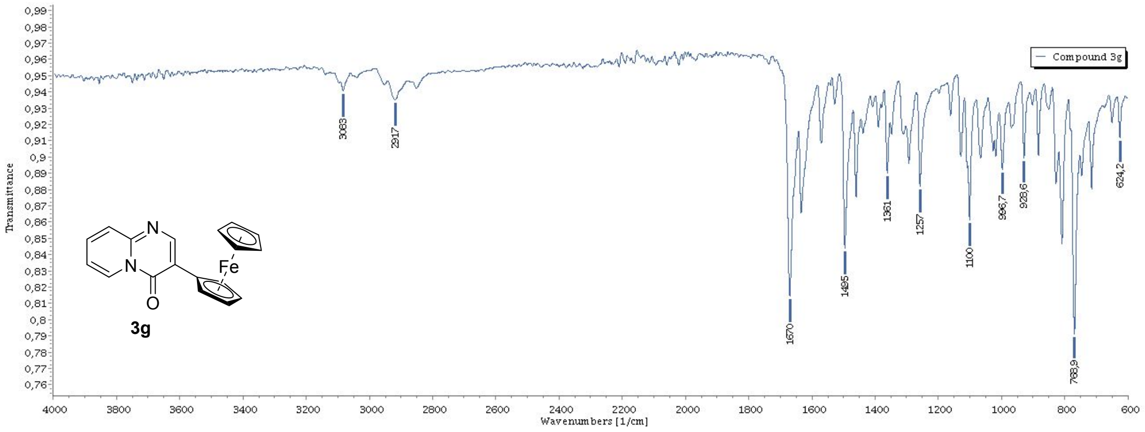
Compound 3g
Transmittance
Wavenumbers [1/cm]
3083
2917
1670
1495
1361
1257
1100
996,7
928,6
783,9
624,2
Fe
3g

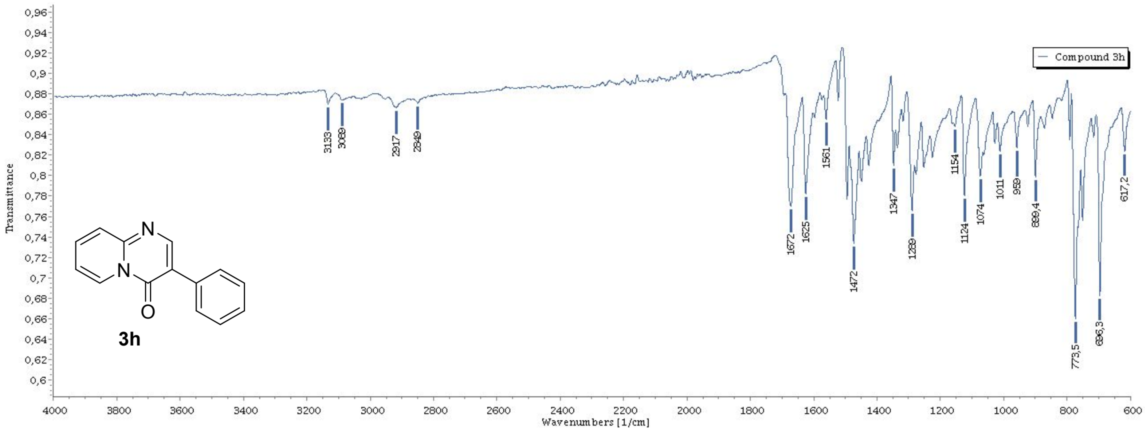
Compound 3h
Transmittance
Wavenumbers [1/cm]
3133
3089
2917
2849
1672
1625
1561
1472
1347
1289
1154
1124
1074
1011
959
899,4
773,5
696,3
617,2
3h

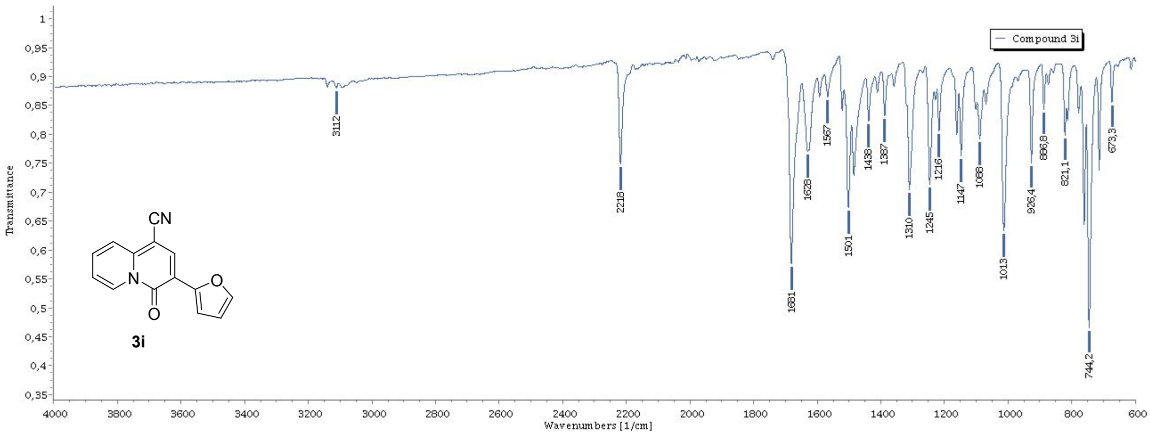
Compound 3i
Transmittance
Wavenumbers [1/cm]
CN
N
O
3i
3112
2218
1681
1628
1587
1501
1438
1387
1310
1245
1216
1147
1088
1013
926,4
886,8
821,1
744,2
673,3

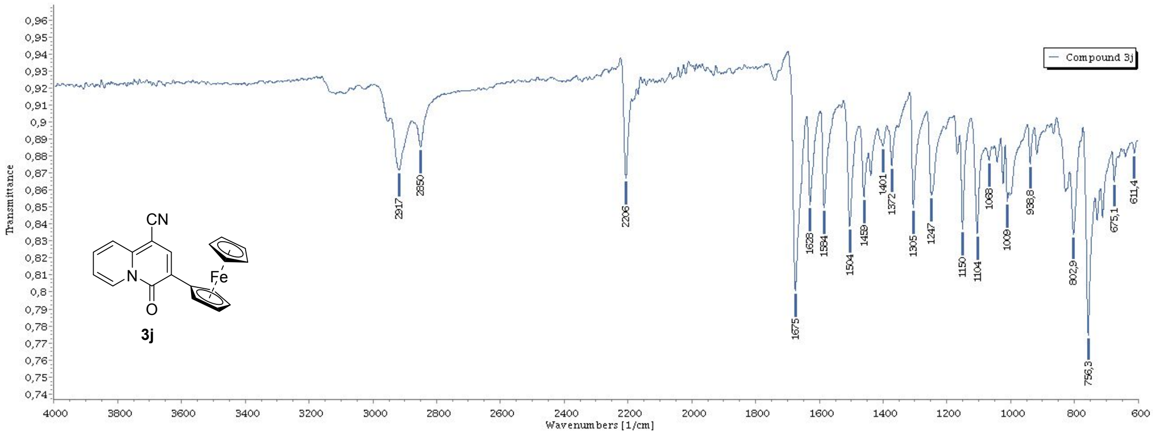
Compound 3j
Transmittance
Wavenumbers [1/cm]
CN
N
O
Fe
3j
2917
2850
2206
1675
1628
1584
1504
1459
1401
1372
1305
1247
1150
1104
1068
1009
938,8
802,9
756,3
675,1
611,4

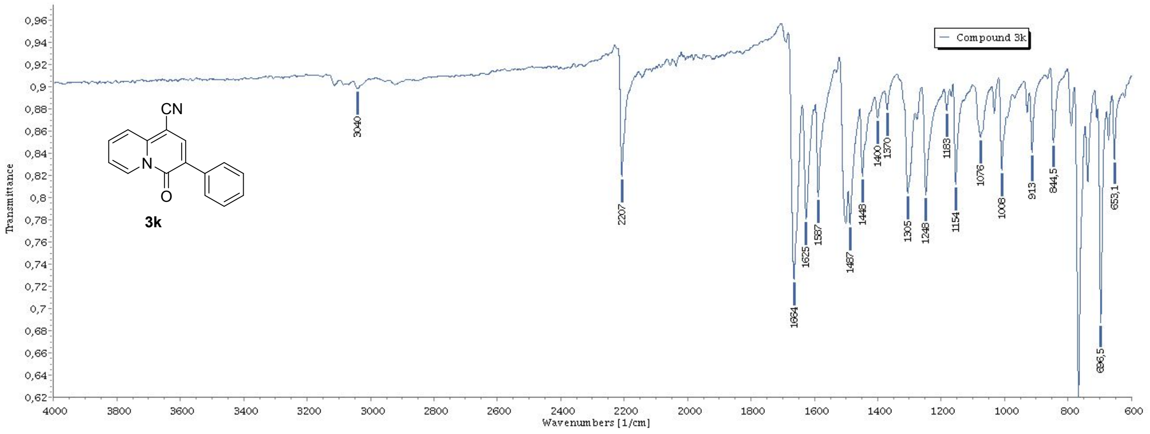
Compound 3k
Transmittance
Wavenumbers [1/cm]
CN
N
O
3k
3040
2207
1664
1625
1587
1487
1448
1400
1370
1305
1248
1183
1154
1076
1008
913
844,5
696,5
653,1

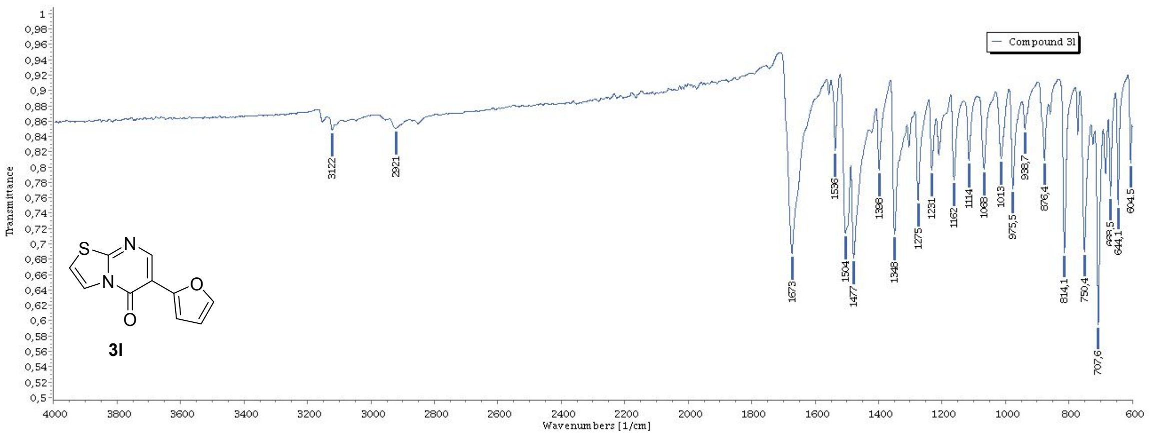
Compound 3l
Transmittance
Wavenumbers [1/cm]
S
N
N
O
3l
3122
2921
1673
1536
1504
1477
1396
1348
1275
1231
1162
1114
1068
1013
975,5
938,7
876,4
814,1
750,4
707,6
644,1
604,5

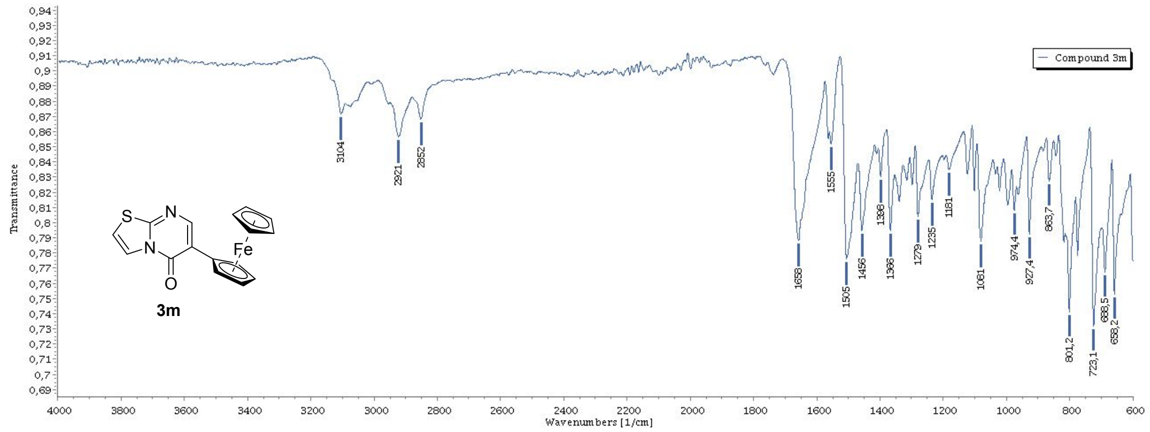

Compound 3m
Transmittance
Wavenumbers [1/cm]
3104
2921
2852
1658
1555
1505
1456
1398
1366
1279
1235
1181
1061
974,4
927,4
863,7
801,2
723,1
688,5
658,2
Fe
3m

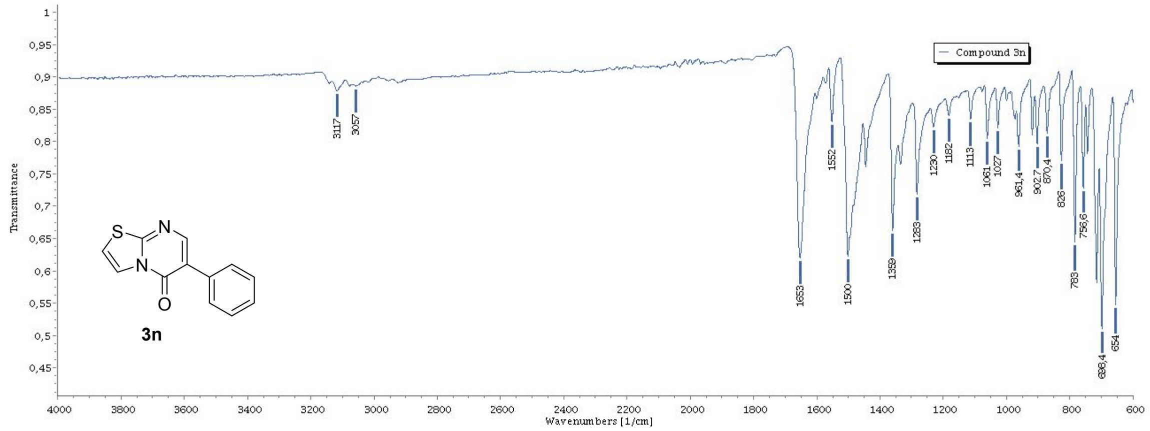

Compound 3n
Transmittance
Wavenumbers [1/cm]
3117
3057
1653
1552
1500
1359
1283
1230
1182
1113
1061
1027
961,4
902,7
870,4
826
783
756,6
698,4
654
3n

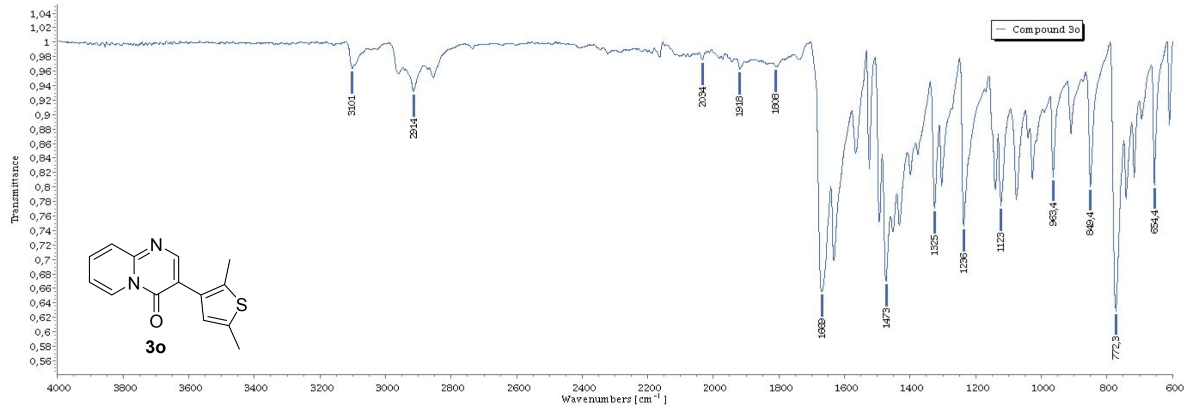

Compound 3o
Transmittance
Wavenumbers [cm⁻¹]
3101
2914
2034
1918
1806
1669
1473
1325
1236
1123
963,4
849,4
772,3
654,4
3o

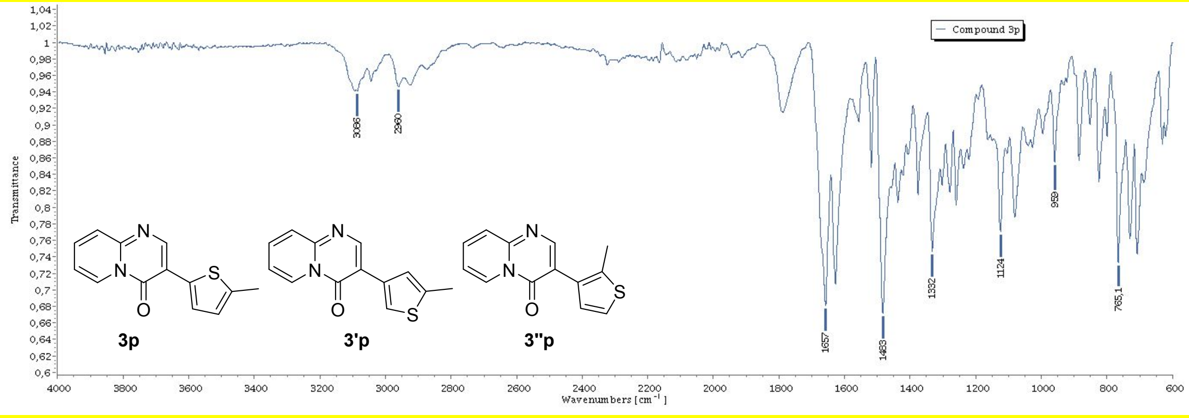

Compound 3p
Transmittance
Wavenumbers [cm⁻¹]
3086
2960
1657
1483
1332
1124
959
765,1
3p
3'p
3''p

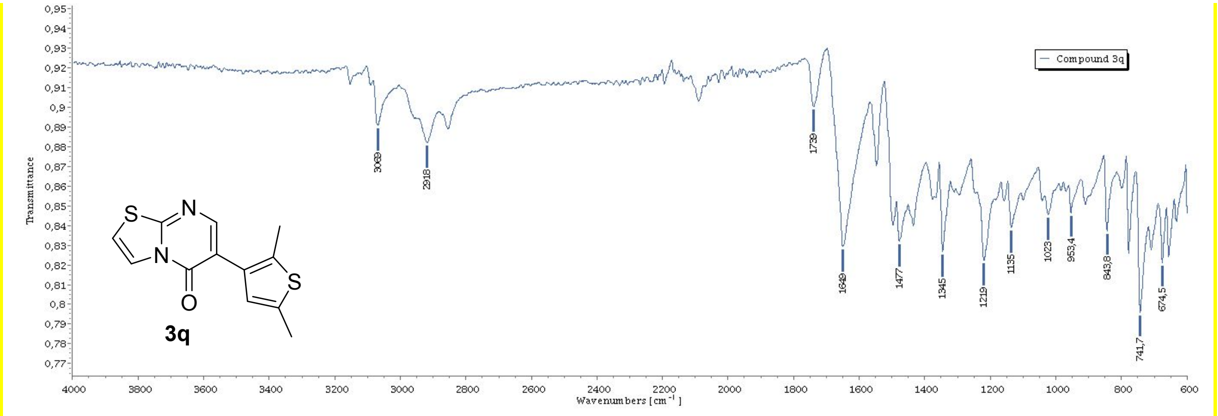
Transmittance
Wavenumbers [cm⁻¹]
Compound 3q
3069
2918
1739
1649
1477
1345
1219
1135
1023
953,4
843,8
741,7
674,5
S
N
N
S
O
3q

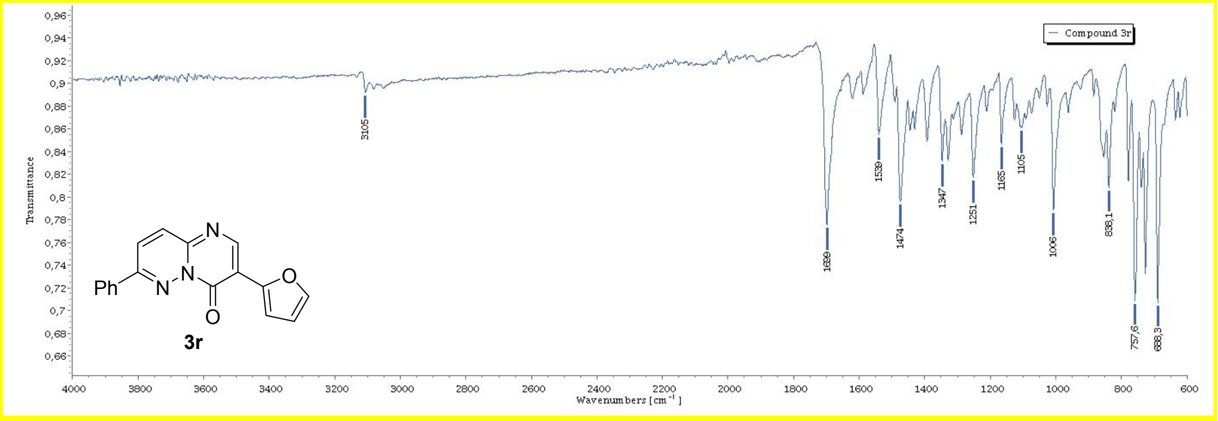
Transmittance
Wavenumbers [cm⁻¹]
Compound 3r
3105
1699
1539
1474
1347
1251
1165
1105
1006
838,1
757,6
698,3
Ph
N
N
N
O
O
3r

4. Structure determination by NMR.

The structures of novel compounds **3a–g**, **3i**, **3j**, **3l**, and **3n–r**, were determined by spectroscopic methods [IR, NMR spectroscopy (^{1}H- and ^{13}C-NMR, COSY, HSQC, HMBC and NOESY spectroscopy), and MS-HRMS] and by elemental analyses for C, H, and N. Physical and spectral data of known arylation products **3h**,[1–3] **3k**,[4] and **3n**[5] were in agreement with the literature data. Ratios of isomers in arylation products **3** were determined from the relative intensities of well-resolved characteristic signals in their ^{1}H- and ^{13}C-NMR spectra (see Section 5, "Copies of NMR spectra"). The structures of each isomer, **3b,f,p**, **3'b,f,p**, and **3''p** in mixtures of regioisomers **3b/3'b**, **3f/3'f**, and **3p/3'p/3''p** were determined by NMR spectroscopy. The structures of model compounds **3b** and **3b'** and assignments of signals for protons and carbon nuclei of each isomer in their ^{1}H- and ^{13}C-NMR spectra were determined by COSY, HSQC, HMBC and NOESY spectroscopy. The structures of **3f,p**, **3'f,p**, and **3''p** were determined by correlation of ^{1}H- and ^{13}C-NMR data with the data for **3b** and **3'b**.Correlation of characteristic δ_H and δ_C chemical shifts and vicinal coupling constants, $^{3}J_{H–H}$, for 4*H*-pyrido[1,2-*a*]pyrimidin-4-one core of compounds **3a**,**b**,**d**,**f**–**h**, **3'b**, and **3'f** is given in Table S1 and Table S2. δ_H Chemical shifts and vicinal coupling constants, $^{3}J_{H–H}$, for compounds **3** and **3'** in $CDCl_3$ are then summarized in Figure S2.

Table S1. Selected ^{1}H NMR spectroscopic data for 4*H*-pyrido[1,2-*a*]pyrimidin-4-one core of compounds **3a,b,d,f–h** and **3'b,f** in $CDCl_3$.

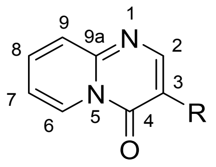

3a,b,d,f-h and **3'b,f**

Compd.	δ (ppm)					$^3J_{H-H}$ (Hz)					
	7–H	9–H	8–H	2–H	6–H	6–7	6–8	6–9	7–8	7–9	8–9
**3a**	7.20	~7.7[a,b]	~7.7[a,b]	8.92	9.18	7.2	1.2	1.2	5.6	2.5	[a,b]
**3b**	7.22	~7.7[a,b]	~7.7[a,b]	8.87	9.21	7.2	1.2	1.2	6.1	1.9	[a,b]
**3b**[c]	7.46	7.79	7.97	9.05	9.12	7.1	1.5	0.8	6.7	1.3	8.5
**3'b**	7.19	~7.7[a,b]	~7.7[a,b]	8.75	9.21	7.2	1.6	1.2	6.3	1.6	[a,b]
**3'b**[c]	7.42	7.75	7.96	8.94	9.13	7.1	1.6	0.8	6.7	1.3	8.6
**3d**	7.17	~7.7[a,b]	~7.7[a,b]	8.88	9.16	7.2	1.1	1.1	5.1	2.9	[a,b]
**3f**	7.15	7.66	7.72	8.35	9.11	7.2	1.6	0.9	6.5	1.5	9.0
**3'f**	7.18	~7.7[a,b]	~7.7[a,b]	8.68	9.18	7.2	1.2	1.2	5.2	2.8	[a,b]
**3g**	7.15	7.64	7.69	8.58	9.17	7.2	1.5	0.9	6.5	1.5	9.0
**3h**[2c]	7.20	7.70	7.75	8.56	9.21	7.2	1.6	0.9	6.5	1.5	9.0

[a]) Multiplet. [b]) Overlaped by other signals. [c]) In DMSO-d_6.

Table S2. δ_C Chemical shifts for 4*H*-pyrido[1,2-*a*]pyrimidin-4-one core of compounds **3** and **3'**.

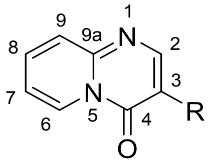

3a,b,d,f-h and **3'b,f**

Compd.	Solvent	δ (ppm)							
		2–C	3–C	4–C	6–C	7–C	8–C	9–C	9a–C
**3a**	$CDCl_3$	149.0	108.9	154.2	127.4	116.1	135.1	126.8	147.9
**3b**	$CDCl_3$	150.1	112.0	155.4	127.7	116.3	135.3	126.7	149.6
**3b**	DMSO-d_6	150.0[a]	110.4	154.6	127.5[a]	117.3[a]	136.8[a]	126.2[a]	149.3
**3'b**	$CDCl_3$	151.5	112.6	156.2	127.6	115.9	135.7	126.6	149.9
**3'b**	DMSO-d_6	151.7[a]	110.9	155.4	127.4	116.9	136.7	126.2	149.6
**3d**	$CDCl_3$	148.2	109.3	154.2	127.3	115.9	134.6	126.7	146.1
**3f**	$CDCl_3$	152.3	112.9	156.8	127.2	115.4	135.5	126.5	149.0
**3'f**	$CDCl_3$	150.1	111.6	155.8	127.3	115.8	134.7	126.6	148.8
**3g**	$CDCl_3$	150.4	116.3	155.7	127.1	115.7	134.5	126.7	149.5
**3h**	$CDCl_3$	153.0	117.1[a]	156.8	127.7	115.9[a]	135.7	126.6	150.7
**3h**	DMSO-d_6	153.0	115.4	156.3	127.7	117.2	137.4	126.4	150.7

[a]) Cross-peak in HSQC NMR spectrum.

Chemical shifts and vicinal coupling constants for protons of the 4*H*-pyrido[1,2-*a*]pyrimidin-4-one core of compounds **3a,b,d,f** and **3'b,f** were almost unaffected by heteroaryl substituents at position 3. Typical δ chemical shifts, 7.2 ppm (7–H) < 7.7 ppm (8–H and 9–H) < 8.9 ppm (2–H) < 9.2 ppm (6–H), as well as vicinal coupling constants, $^3J_{7H-8H} \sim 6$ Hz < $^3J_{6H-7H} \sim 7$ Hz < $^3J_{8H-9H} \sim 9$ Hz, were in line with the literature values for related 4*H*-pyrido[1,2-*a*]pyrimidin-4-one derivatives (Figure S2A).[1–3,6,7] Also δ_H chemical shifts and vicinal coupling

constants, $^3J_{H\text{–}H}$, for protons of the 3-(hetero)aryl residues of compounds **3a,b,d,f** and **3'b,f** were in agreement with characteristic literature data for the corresponding pyrrole, furan, and thiophene derivatives (Figure S2B).[8–11] In the furan series (compounds **3a** and **3d**), chemical shifts, δ_H 4'–H < 3'–H < 5–H and vicinal coupling constants, $^3J_{3H\text{–}5H}$ = 0.8 Hz < $^3J_{4H\text{–}5H}$ = 1.8 Hz < $^3J_{3H\text{–}4H}$ ~ 3.3 Hz, were also in line with typical literature values for 2-substituted furan derivatives (Figure S2B, central column).[9,11] In the thiophene series (regioisomeric compounds **3b** and **3'b**), chemical shifts, δ_H 7.15 ppm (4'–H) < 7.41 ppm (5'–H) < 7.7 ppm (5'–H) and vicinal coupling constants, $^3J_{3H\text{–}5H}$ = 1.1 Hz < $^3J_{3H\text{–}4H}$ = 3.7 Hz < $^3J_{4H\text{–}5H}$ = 5.2 Hz in the 2'-substituted isomer **3b**, as well as chemical shifts, δ_H 7.4 ppm (5'–H) < 7.63 ppm (4'–H) < 8.23 ppm (2'–H) and vicinal coupling constants, $^3J_{2H\text{–}4H}$ = 1.3 Hz < $^3J_{2H\text{–}5H}$ = 3.0 Hz < $^3J_{4H\text{–}5H}$ = 5.1 Hz in 3'-substituted isomer **3'b** were in agreement with literature data for the respective 2- and 3-substituted thiophene derivatives (Figure S2B, right column).[10,11] In the pyrrole series (regioisomeric compounds **3f** and **3'f**), chemical shifts, δ_H 6.27 ppm (4'–H) ≤ 6.31 ppm (3'–H) < 7.42 ppm (5–H) and vicinal coupling constants, $^3J_{3H\text{–}5H}$ ~ 1.8 Hz < $^3J_{3H\text{–}4H}$ = $^3J_{4H\text{–}5H}$ = 3.3 Hz in 2'-substituted isomer **3f**, as well as chemical shifts, δ_H 6.73 ppm (4'–H) < 7.36 ppm (5'–H) < 8.20 ppm (2'–H) and vicinal coupling constants, $^3J_{2H\text{–}4H}$ = $^3J_{2H\text{–}5H}$ = 1.9 Hz < $^3J_{4H\text{–}5H}$ = 3.4 Hz in 3'-substituted compound **3'f** were in agreement with literature data for the respective 2- and 3-substituted 1-acylpyrrole derivatives (Figure S2B, left column).[8,11]

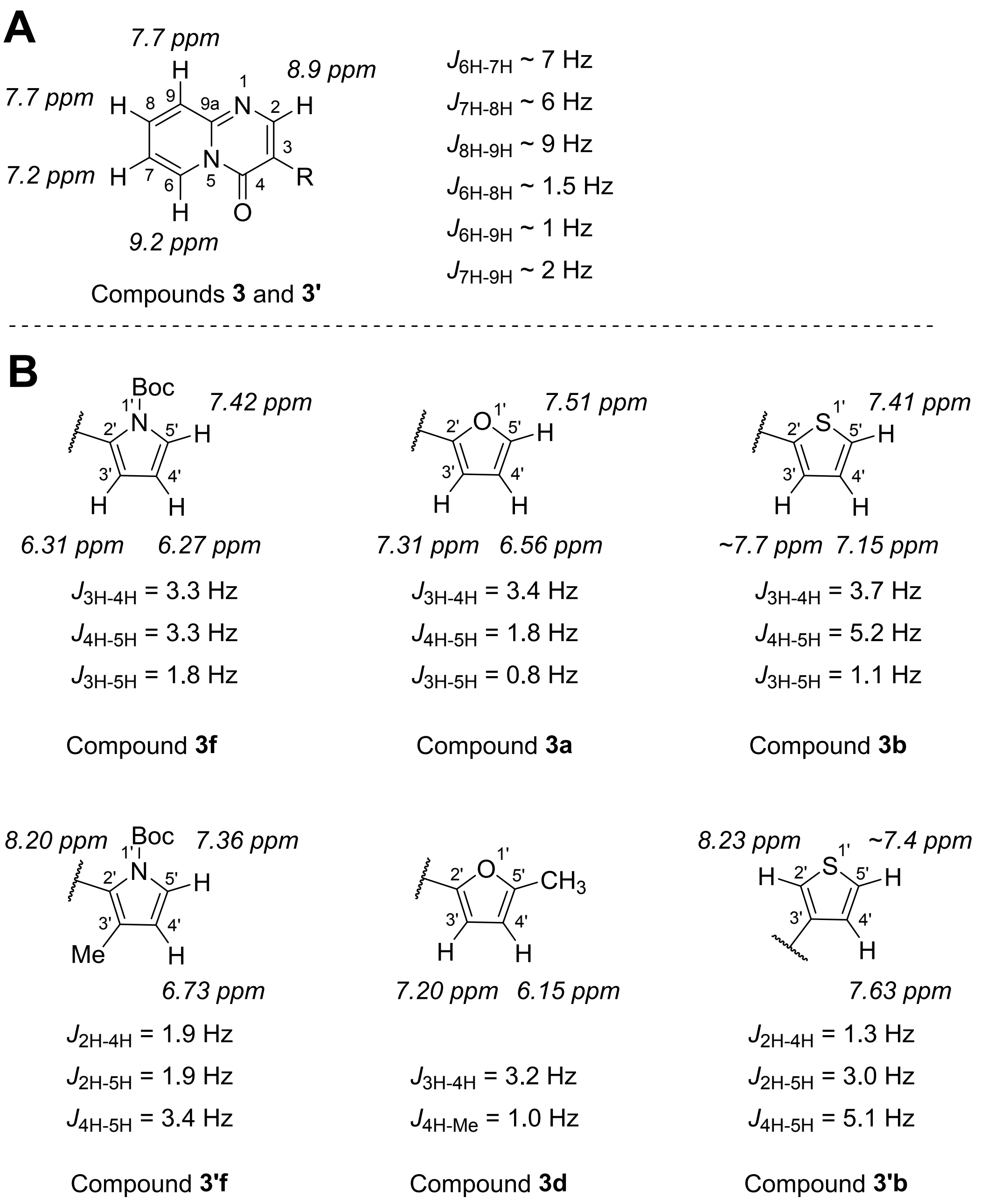

Figure S2. A: Characteristic δ_H chemical shifts and vicinal coupling constants, $^3J_{H\text{–}H}$, for the 4*H*-pyrido[1,2-*a*]pyrimidin-4-one core of compounds **3** and **3’** in $CDCl_3$. **B**: Characteristic δ_H chemical shifts and vicinal coupling constants, $^3J_{H\text{–}H}$, for the 3-heteroaryl residues of compounds **3a,b,d,f** and **3’b,f** in $CDCl_3$.

In the same way as described above for compounds **3b** and **3’b** (cf. Figure S2), also the structures of regioisomeric arylation products **3p**, **3’p**, and **3’’p** were determined by ^1H NMR.

Chemical shifts and vicinal coupling constants for compounds **3p**, **3’p**, and **3’’p** were in agreement with spectral data for compounds **3b** and **3’b** (cf. Figure S2B) and with literature data for the respective 2- and 3-substituted thiophene derivatives (Figure S3).[10,11]

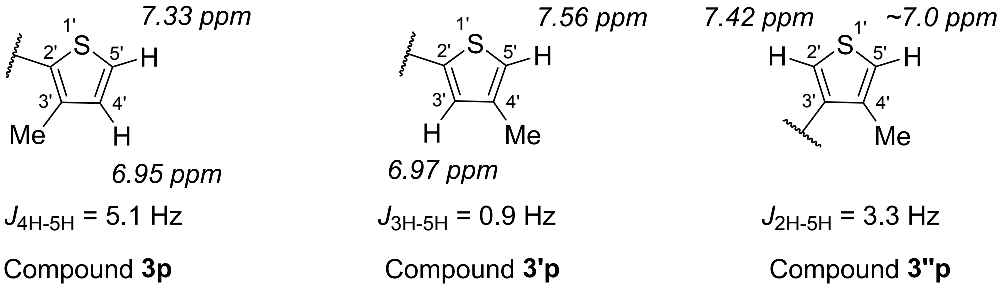

Figure S3. Characteristic δ_H chemical shifts and vicinal coupling constants, $^3J_{H\text{–}H}$, for compounds **3p**, **3’p**, and **3’’p** in $CDCl_3$.

The structures of regioisomeric thiophene derivatives **3b** and **3’b** were additionally determined by 2D NMR spectroscopy. ^{1}H NMR, ^{13}C NMR, COSY, NOESY, HSQC, and HMBC spectra of a 81:19 mixture of the major isomer **3b** and the minor isomer **3’b** were taken in DMSO-d_6. First, assignments of the vicinally coupled protons (cf. Table S2, Figure S4) were confirmed by COSY experiment. As shown in Figure S4, cross-peaks between the coupled protons are in agreement with the proposed assignment of the signals for both isomers, **3b** and **3’b**. In the pyrido[1,2-*a*]pyrimidine core the signal for 7-H at 7.46 ppm has cross-peaks with signals for 8-H at 7.98 ppm and 6-H at 9.12 ppm, the signal for 9-H at 7.79 ppm correlates with the signal for 8-H at 7.98 ppm, whereas 2-H does not correlate with other protons. In the 2-thienyl residue of the major isomer **3b**, the signal for 4’-H at 7.16 ppm correlates with signals for 5’-H at 7.57 ppm and 3’-H at 7.86 ppm. In the 3-thienyl residue of the minor isomer **3’b**, the signal for 5’-H at 7.64 ppm correlates with signals for 4’-H at 7.84 ppm and 2’-H at 8.33 ppm (Figure S4, cf. Figure S2, Table S1).

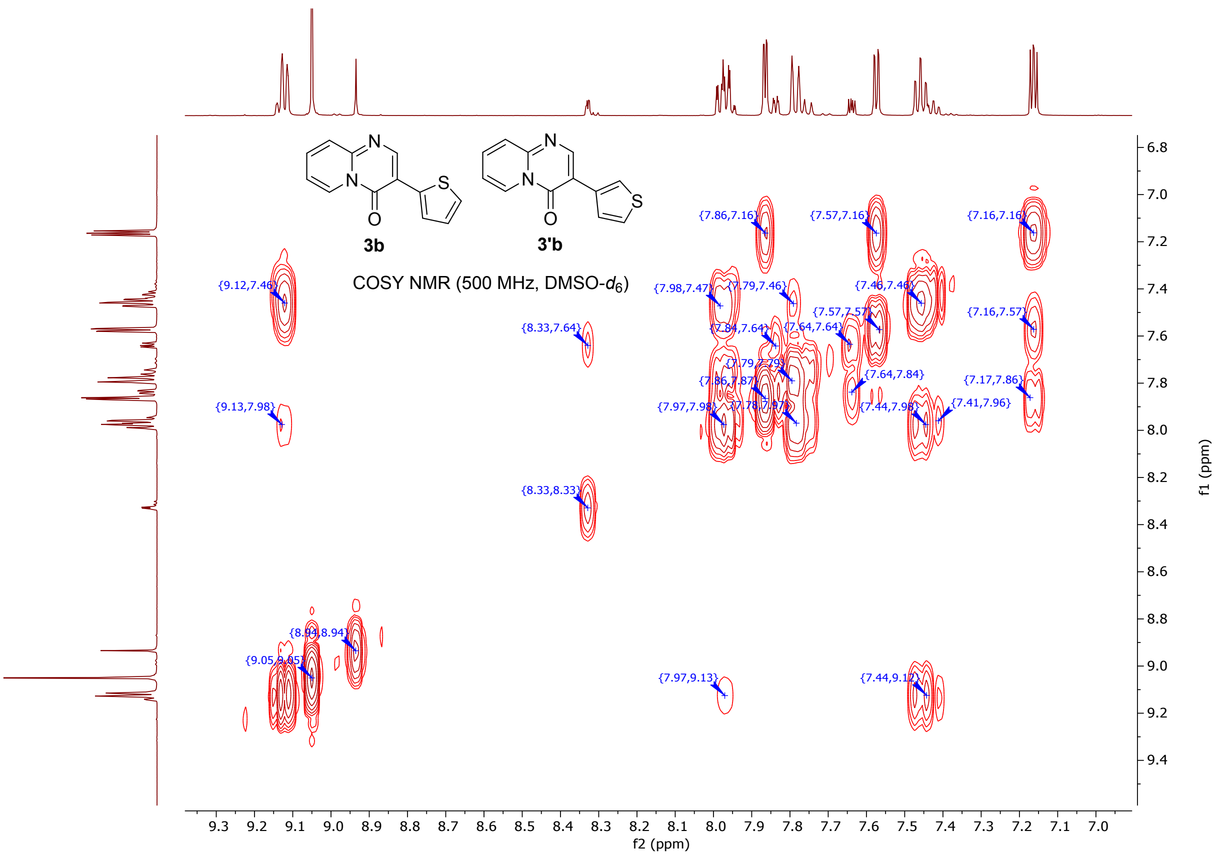

Figure S4. Partial COSY spectrum of a 4:1 mixture of compounds **3b** and **3'b** in DMSO-d_6.

The regiochemistry of compound **5b** was confirmed by NOESY spectroscopy. NOE between 2–H at 9.05 ppm and 3'–H at 7.86 ppm in the major isomer **5b** was in agreement with the 2'-thienyl isomer **5b** (Figure S5).

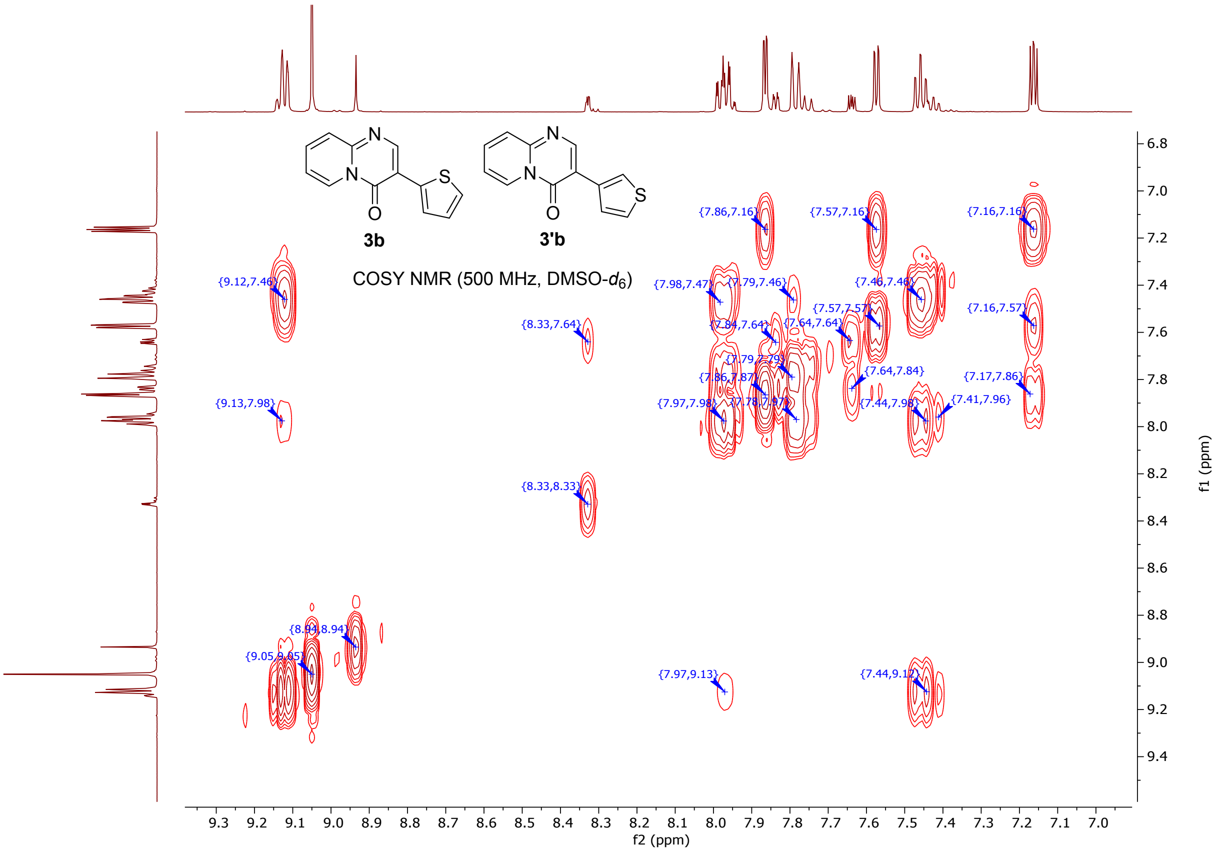

Figure S5. Partial COSY spectrum of a 4:1 mixture of compounds **3b** and **3'b** in DMSO-d_6.

The regiochemistry of compound **3b** was confirmed by NOESY spectroscopy. NOE between 2–H at 9.05 ppm and 3'–H at 7.86 ppm in the major isomer **3b** was in agreement with the 2'-thienyl isomer **3b** (Figure S6).

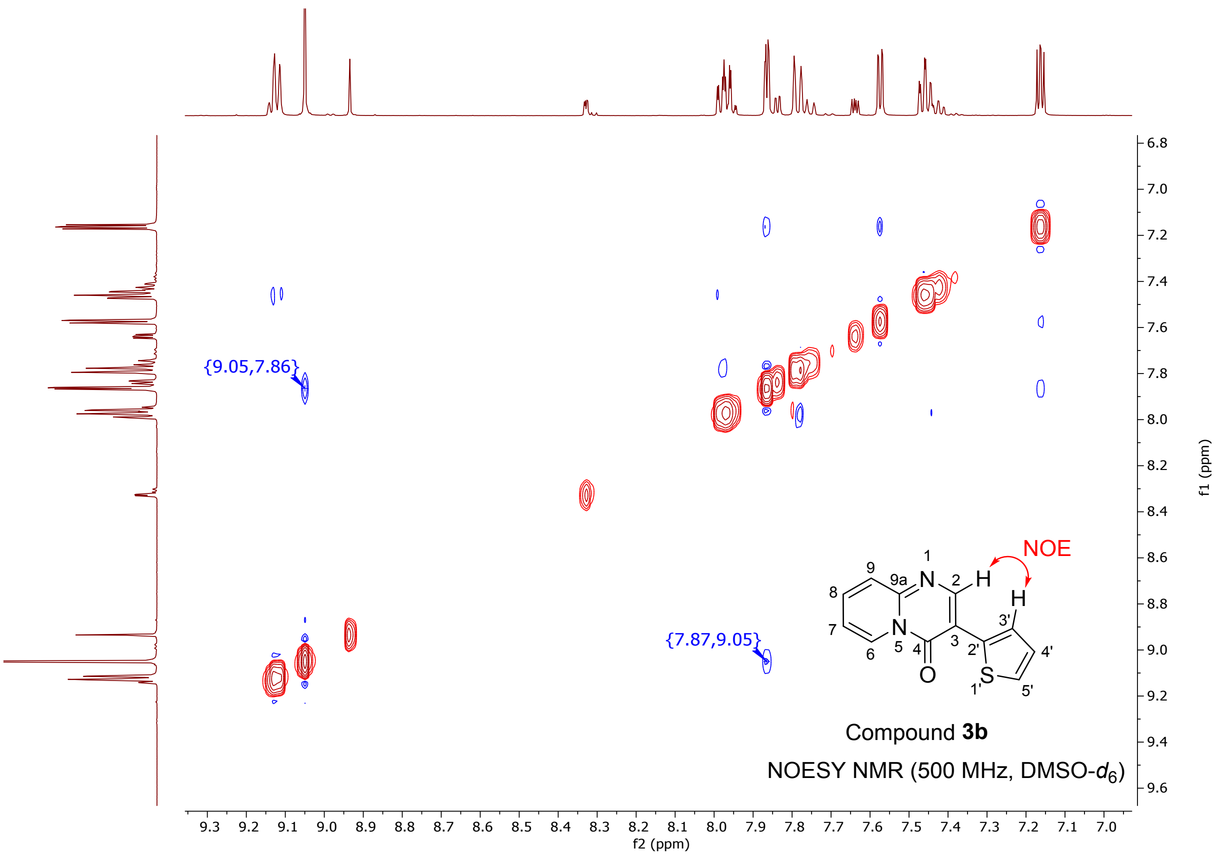

Figure S6. Partial NOESY spectrum of a 4:1 mixture of compounds **3b** and **3’b** in DMSO-d_6 and determination of a structure of compound **3b** by NOE between 2-H and 3’-H.

Next, the methine carbon nuclei (C–H) for compounds **3b** and **3’b** were assigned by HSQC spectroscopy. Partial HSQC spectrum of a 81:19 mixture of compounds **3b** and **3’b** is presented in Figure S7 and the results are summarized in Table S3. In this manner, the signals of methine carbon nuclei in ^{13}C NMR spectrum at positions 2, 6–9, and 3’–5’ for the major isomer **3b** and at positions 2, 6–9, 2’, 4’, and 5’ for the minor isomer **3’b** were assigned (Figure S7).

A

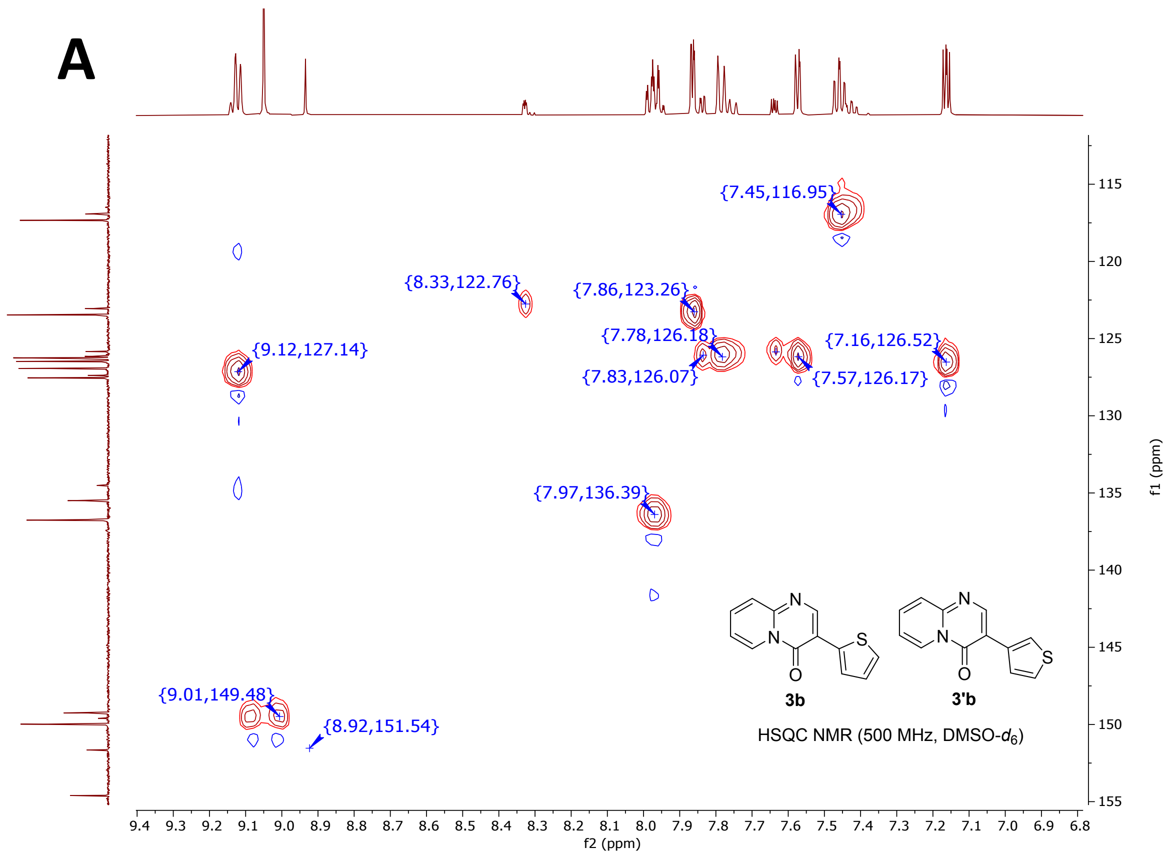

B

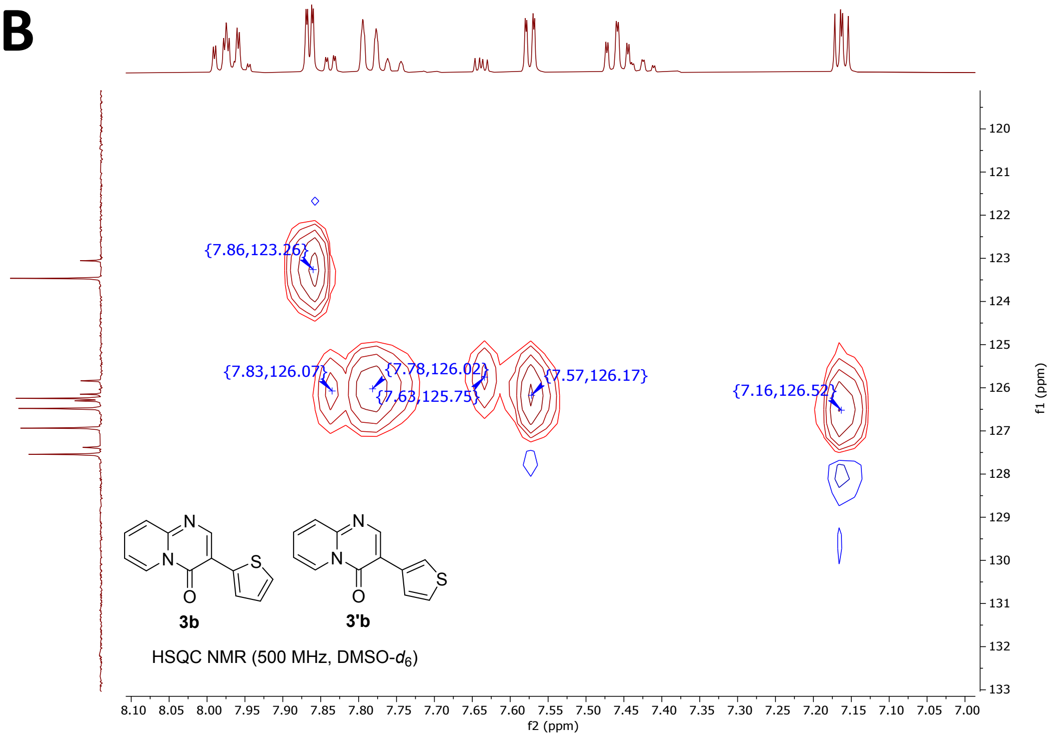

Figure S7. A: Partial HSQC spectrum of **3b**/**3'b** in DMSO-d_6 with marked correlations at all positions. **B**: Zoomed region of the above spectrum with correlations at positions 9, 4', and 5'.

Finally, the signals for quaternary carbon nuclei for compounds **3b** and **3’b** were assigned on the basis of HMBC spectrum of **3b/3’b**. For both isomers, the signals for the 3-C at around 110 ppm and 4-C at around 155 ppm both have cross-peaks with the respective 2–H, while the signals for 9a–C at around 149 ppm have cross-peaks with 2–H, 8–H and 9–H. Thiophene’s quaternary 2’–C of the major isomer **3b** at around 135 ppm correlates with 2–H, 3’–H, 4’–H, and 5’–H, while quaternary 3’–C of the minor isomer **3’b** at around 134 ppm correlates with 2–H, 2’–H, 4’–H, and 5’–H (Figure S8).

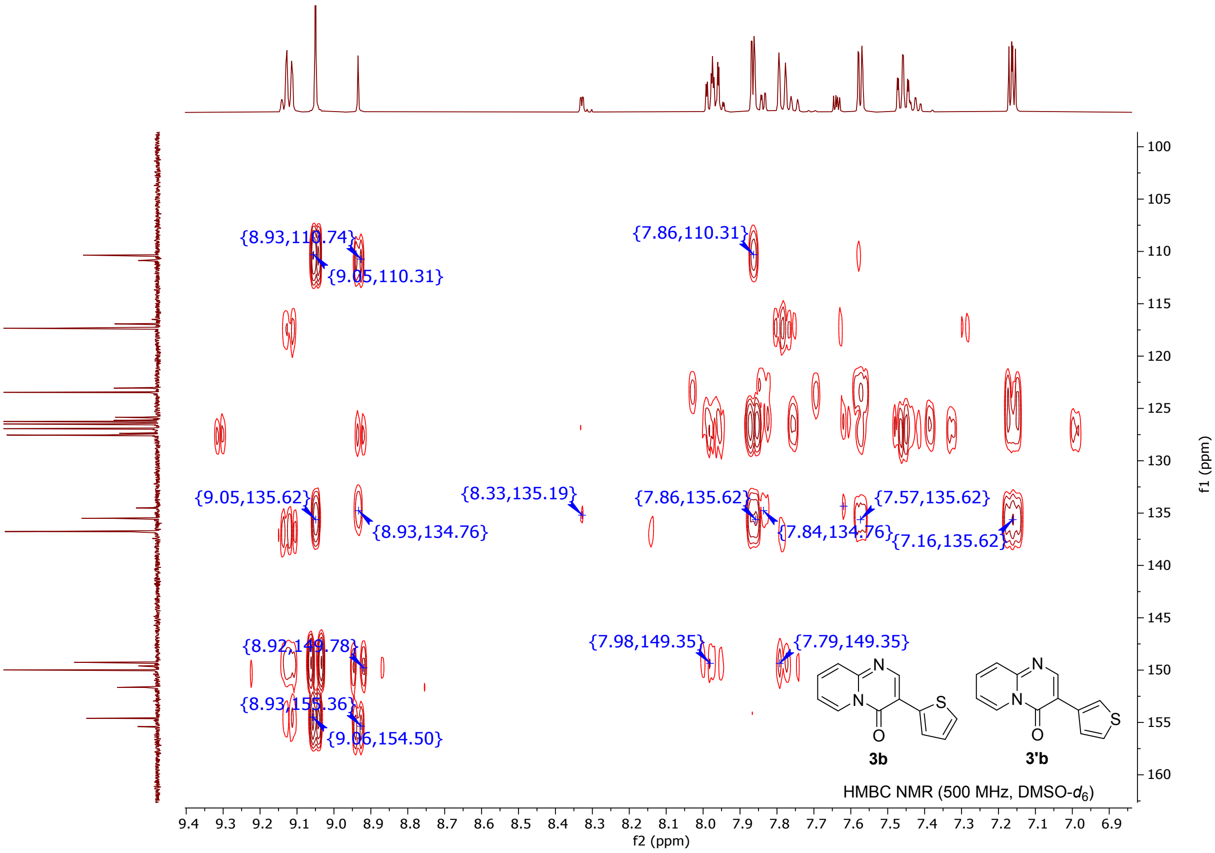

Figure S8. Partial HMBC spectrum of **3b**/**3’b** in DMSO-d_6 showing multiple bond correlations of quaternary carbon nuclei at positions 3, 4, 9a, 2’, and 3’ with protons at positions 2, 8, 9, 3’, and 4’.

Table S3. ^{1}H NMR and ^{13}C NMR δ chemical shifts (ppm) and 2D NMR data for regioisomeric compounds **3b** and **3’b**.

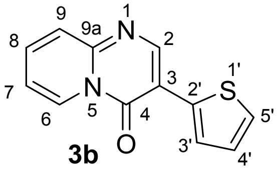

3b

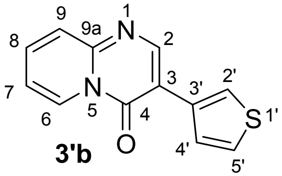

3'b

Compound	Experiment	Solvent	2	3	4	6	7	8	9	9a	2’	3’	4’	5’
**3b**	^{1}H	$CDCl_3$	8.87	-	-	9.21	7.22	~7.7^{a,}	~7.7^{a,b}	-	-	~7.7^{a,b}	7.15	7.41
**3b**	^{13}C	$CDCl_3$	150.1	112.0	155.4	127.7	116.3	135.3	126.2	149.6	135.3	124.1	127.1	126.7
**3b**	^{1}H	DMSO-d_6	9.05^{b}	-	-	9.12^{b}	7.46^{b}	7.97^{b}	7.79^{b}	-	-	7.86^{b}	7.16^{b}	7.57^{b}
**3b**	^{13}C	DMSO-d_6	150.0^{b}	110.4	154.6	127.5^{b}	117.3^{b}	136.8^{b}	126.25^{b}	149.3	135.5	123.5^{b}	126.9^{b}	126.5^{b}
**3’b**	^{1}H	$CDCl_3$	8.75	-	-	9.21	7.19	~7.7^{a,b}	~7.7^{a,b}	-	8.23	-	7.63	7.41
**3’b**	^{13}C	$CDCl_3$	151.5	112.6	156.2	127.6	115.9	135.7	125.9	149.9	123.9	134.1	126.6	125.5
**3’b**	^{1}H	DMSO-d_6	8.94^{b}	-	-	9.13	7.42	7.96	7.75	-	8.33^{b}	-	7.84^{b}	7.64^{b}
**3’b**	^{13}C	DMSO-d_6	151.7^{b}	110.9	155.4	127.4	116.9	136.7	126.15	149.6	123.1^{b}	134.5	126.3^{b}	125.8^{b}

^{a}) Overlaped by other signals. ^{b}) Cross-peak in HSQC NMR spectrum.

5. Elucidation of the reaction mechanism.

5.1. Absorption properties of diazonium salts 1a–c and absorption and emission properties of the product 3a.

Absorption spectra of diazonium salts **1a**–**c** are shown in Figures S9–S11, while normalized absorption spectra of **1a**–**c** are presented in Figure S12. Compounds **1a**–**c** exhibit absorption maxima between 200 and 450 nm. Among these three compounds, only quinolizine-diazonium salt **1a** exhibits two absorption maxima in the vis-region (404 nm and 425 nm), while absorption maxima of pyrido[1,2-*a*]pyrimidine-diazonium salt **1b** (371 nm and 385 nm) and thiazolo[3,2-*a*]pyrimidine-diazonium tetrafluoroborate **1c** (365 nm) are in the UV-A region (Figures S9–S11). As compounds **1a**–**c** do not absorb at wavelengths longer than 500 nm, they cannot be excited with green light (510 nm), which was used in photocatalytic arylations. These absorption properties of **1a**–**c** did not interfere with the absorption maximum of **EY-Na$_2$** at 520 nm.

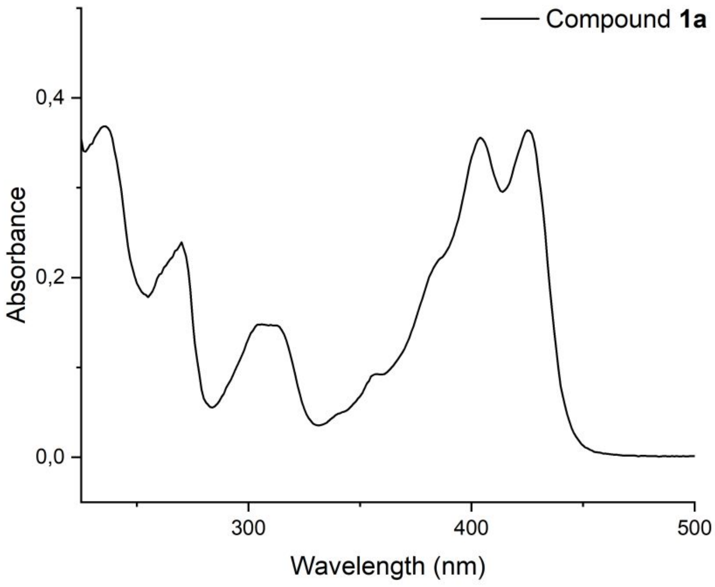

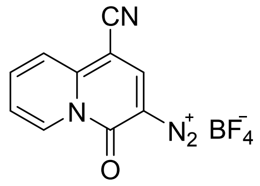

1a

Figure S9. Absorption spectrum of 1-cyano-4-oxo-4*H*-quinolizine-3-diazonium tetrafluoroborate (**1a**).

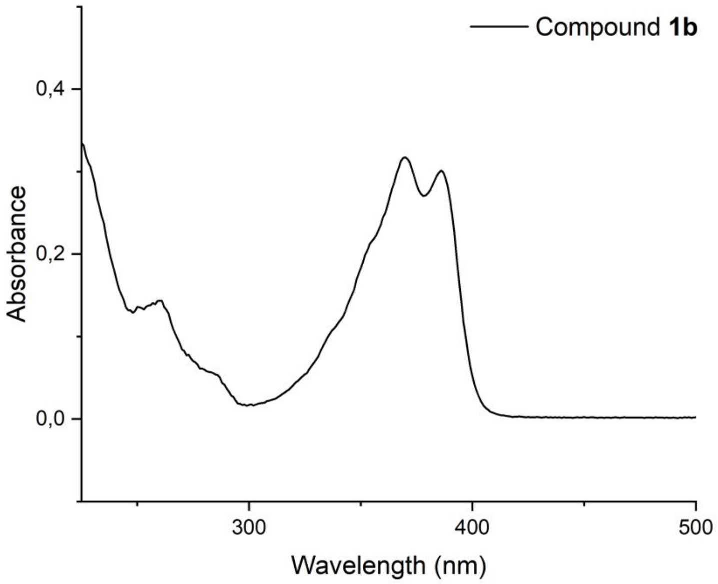

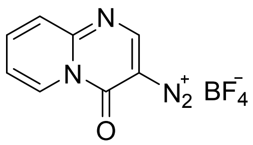

1b

Figure S10. Absorption spectrum of 4-oxo-4*H*-pyrido[1,2-*a*]pyrimidine-3-diazonium tetrafluoroborate (**1b**).

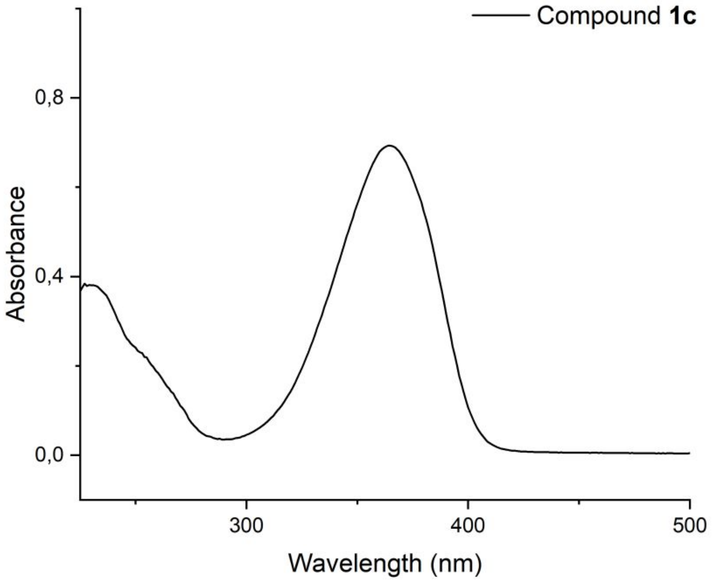

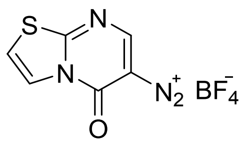

1c

Figure S11. Absorption spectrum of 5-oxo-5*H*-thiazolo[3,2-*a*]pyrimidine-6-diazonium tetrafluoroborate (**1c**).

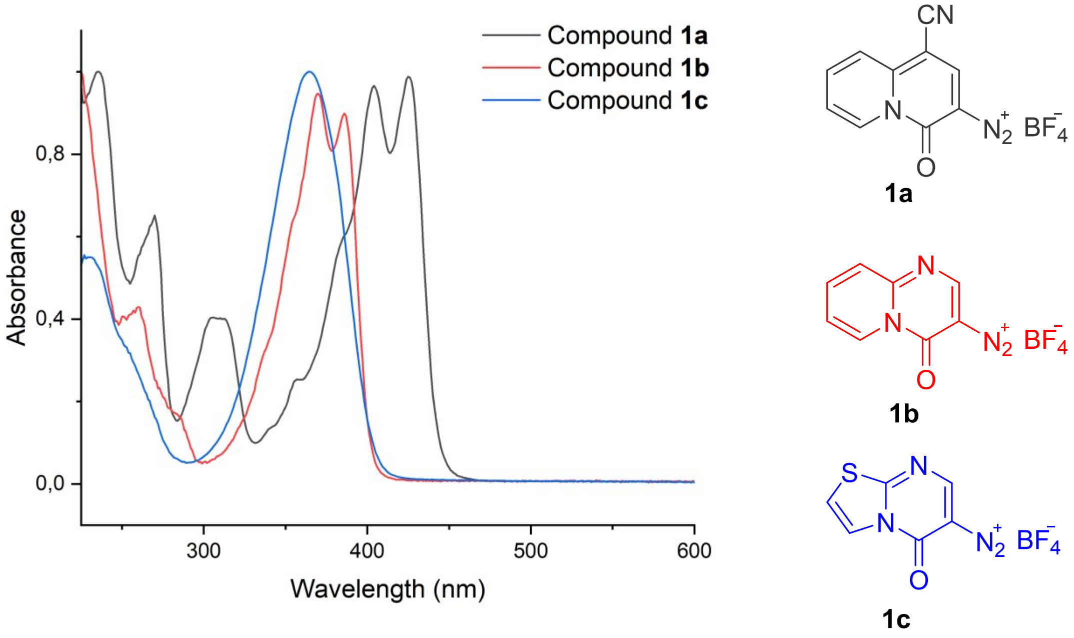

Figure S12. Normalized absorption spectra of diazonium tetrafluoroborates **1a**–**c**.

Absorption spectrum of 3-(furan-2-yl)-4*H*-pyrido[1,2-*a*]pyrimidin-4-one (**3a**), the product of the model reaction (**1b** + **2a** → **3a**), is shown in Figure S13. Compound **3a** does not absorb light at wavelengths above 450 nm. Consequently, it cannot act as a photocatalyst in the reaction performed under irradiation with green light (510 nm).

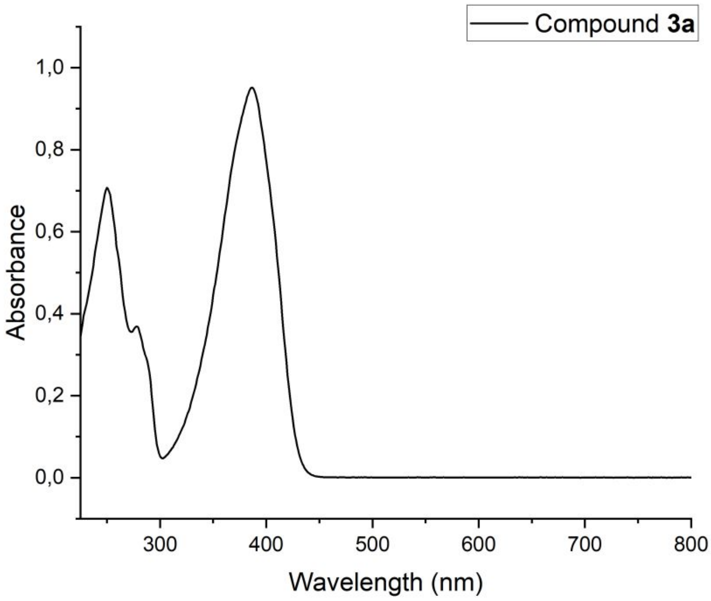

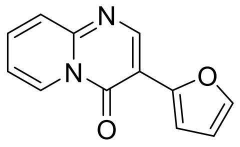

3a

Figure S13. Absorption spectrum of 3-(furan-2-yl)-4*H*-pyrido[1,2-*a*]pyrimidin-4-one (**3a**).

By monitoring the reaction progress by TLC most of arylation products **3a**–**f**,**h**,**i**,**k**,**l**,**n** exhibited yellow, green, or blue fluorescence upon irradiation with UV-light (366 nm) in solution and in the solid state. However, ferrocenyl-substituted compounds **3g**,**j**,**m** were not fluorescent. Examples of fluorescent compounds **3a,c,e,h,l** are shown in Figure S14.

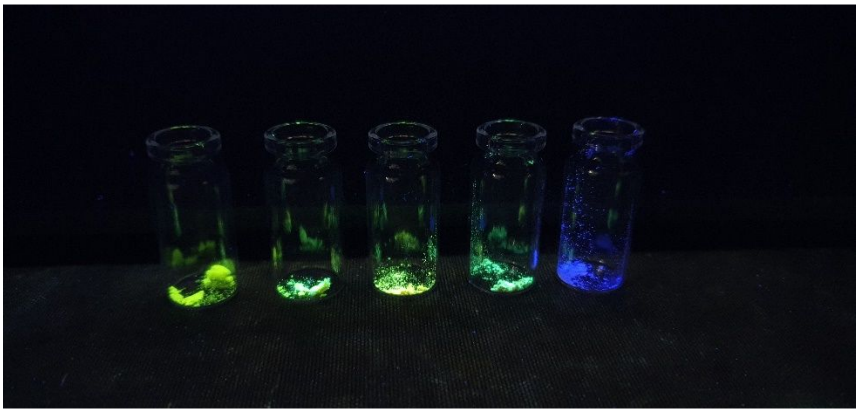

Figure S14. Fluorescence of compounds (from left to right) **3a**, **3e**, **3c**, **3l**, and **3h** in the solid state under UV-light (366 nm).

Emission spectrum of **3a** in MeCN exhibited emission maximum at 475 nm (Figure S15).

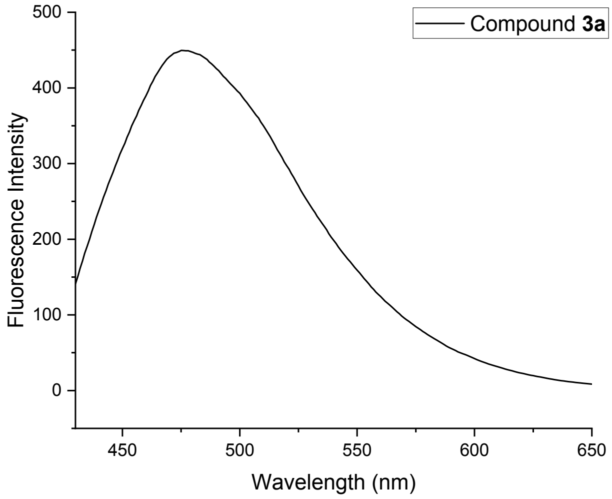

Figure S15. Emission spectrum of 3-(furan-2-yl)-4*H*-pyrido[1,2-*a*]pyrimidin-4-one (**3a**) at an excitation wavelength of 389 nm.

5.2. C–H Arylations of diazonium salts 1a–c performed in the presence of TEMPO.

The model reaction **1b** + **2a** → **3a** was performed also in the presence of TEMPO as radical scavenger. When the reaction was performed in the presence of TEMPO, the adduct **4** of TEMPO and radical **1b•** was detected by LC-HRMS (Table S4, entries 2 and 3). Next, the NMR yield of arylation product **3a** dropped significantly, from 77% without TEMPO to 9% with TEMPO (Table S4, entries 1 and 2). This result was consistent with the formation of heteroaryl radical **1b•** from the respective diazonium salt **1b**, also in the absence of furan (**2a**) (Table S4, entry 3). Addition of TEMPO also decreased the yield of **3a**, when the reaction was carried out under air in non-degassed solvent (Table S4, entries 4–6).

Table S4. Model transformation (**1a** + **2a** → **3a**) performed in the presence and in the absence of TEMPO.[a]

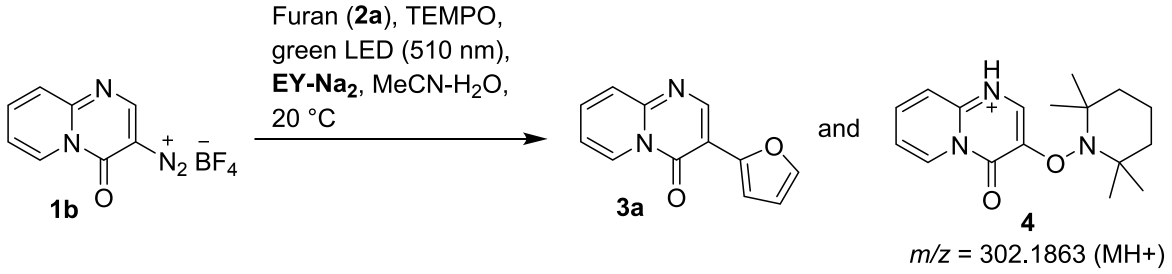

Entry	Equiv. of **2a**	Time (h)	Atmosphere	λ (nm)	Equiv. of **EY-Na₂**	Equiv. of TEMPO	Yield of **3a** (%)[b]
1[c]	10	4	N_2	510	0.01	0	77
2	10	4	N_2	510	0.01	2[d]	9
3	0	4	N_2	510	0.01	2[d]	0
4[c]	10	4	air[e]	[f]	0	0	3
5	10	21	air[e]	[f]	0	0	45
6	10	24	air[e]	[f]	0	2[d]	17

[a]) Reaction conditions: diazonium salt **1b** (0.2 mmol), furan (**2a**), degassed MeCN:H_2O (9:1, 1 mL), eosin Y, TEMPO, green LED, T = 20 °C, t = 4 h. [b]) NMR yield. [c]) Given also in Table 1 in the main manuscript. [d]) Adduct **4** detected by LC-HRMS. [e]) Non-degassed solvent was used for air atmosphere. [f]) No light.

5.3. Kinetic measurements.

The kinetic profiles of the model transformation (**1b** + **2a** → **3a**) were determined by ^{1}H NMR using dimethylsulfone as internal standard. 50 μL aliquots were taken out from the reaction mixture in suitable time intervals, diluted with $CDCl_3$, and ^{1}H NMR spectra were taken. Essentially, standard reaction conditions (510 nm, 20 °C, 1 mol% eosin Y, MeCN–H_2O, 9:1) were employed and the following parameters were varied:

a) the presence or absence of light,

b) the presence or absence of catalyst, and

c) temperature (20 °C or 50 °C).

The kinetic profiles under various reaction conditions are presented in Figures S16–S20.

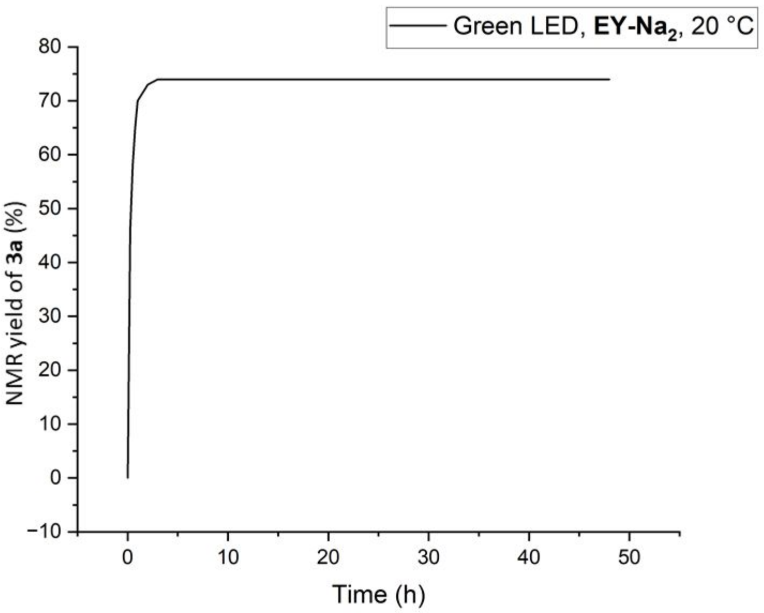

Figure S16. Kinetic profile of model reaction under standard conditions (510 nm, 20 °C, 1 mol% **EY-Na$_2$**, MeCN–H_2O, 9:1).

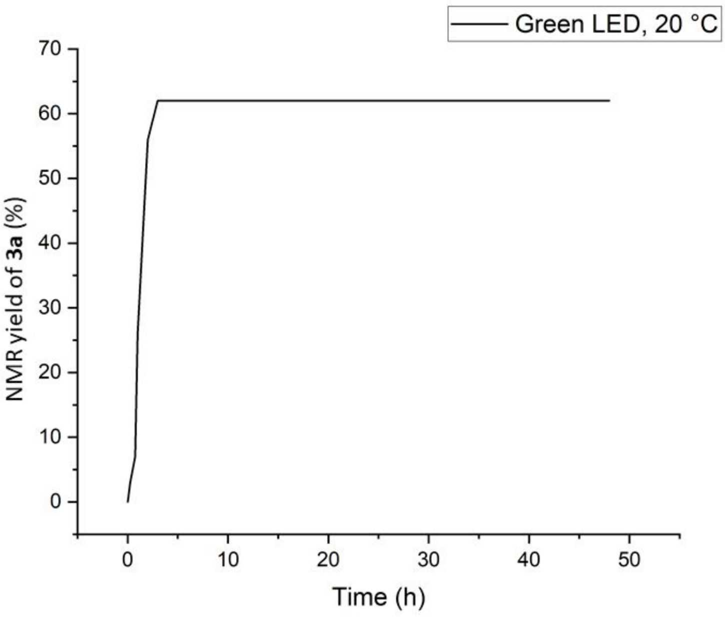

Figure S17. Kinetic profile of model reaction under modified standard conditions (510 nm, 20 °C, MeCN–H_2O, 9:1).

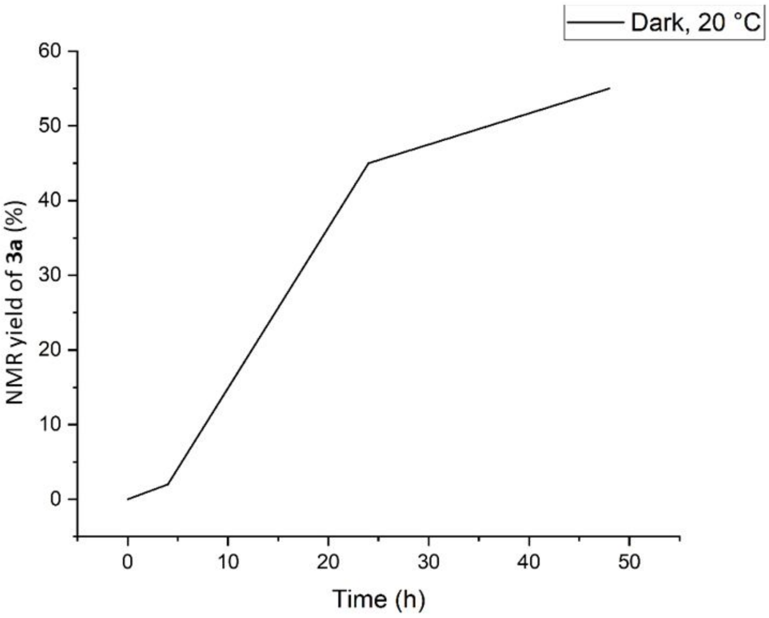

Figure S18. Kinetic profile of model reaction under modified standard conditions (dark, 20 °C, MeCN–H_2O, 9:1).

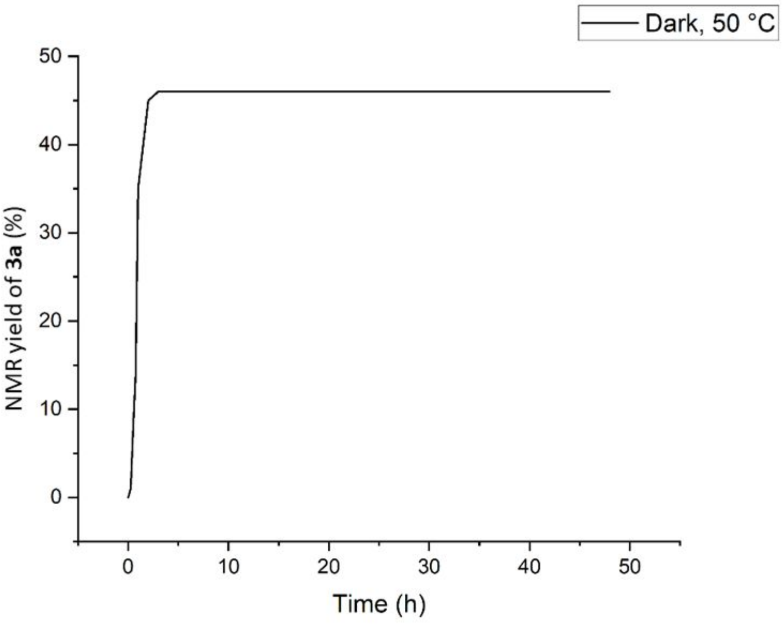

Figure S19. Kinetic profile of model reaction under modified standard conditions (dark, 50 °C, MeCN–H_2O, 9:1).

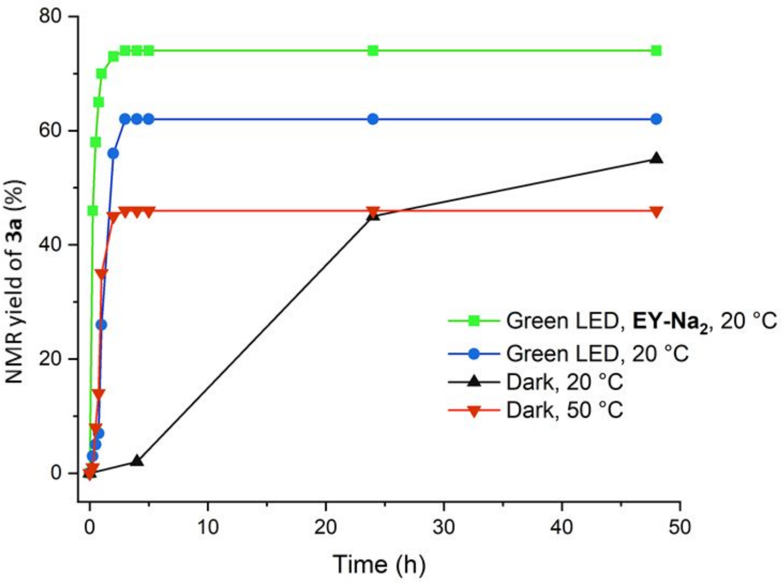

Figure S20. The kinetic profiles of model transformation **1b** + **2a** → **3a** within 48 h reaction time under photochemical conditions in the presence (▬) or absence (▬) of eosin Y and under thermal conditions in the absence of **EY-Na$_2$** at 20 °C (▬) and 50 °C (▬).

6. X-Ray diffraction analysis data for compound 3n.

Single-crystal X-ray diffraction data was collected on Agilent Technologies SuperNova Dual diffractometer with an Atlas detector using monochromated Mo-Kα radiation (λ = 0.71073 Å) at 150 K. The data was processed using CrysAlis PRO.[12] Using Olex2.1.2.,[13] the structures were solved by direct methods implemented in SHELXS[14] or SHELXT[15] and refined by a full-matrix least-squares procedure based on F^2 with SHELXT-2014/7.[16] All nonhydrogen atoms were refined anisotropicallly. Hydrogen atoms were placed in geometrically calculated positions and were refined using a riding model. The drawings (Figure S21) and the analysis of bond lengths, angles and intermolecular interactions were carried out using Mercury[17] and Platon.[18]

Structural and other crystallographic details on data collection and refinement for compound **3n** (Table S5) have been deposited with the Cambridge Crystallographic Data Centre as supplementary publication number CCDC Deposition Number 2266496. These data can be obtained free of charge via www.ccdc.cam.ac.uk/conts/retrieving.html (or from the CCDC, 12 Union Road, Cambridge CB2 1EZ, UK; fax: +44 1223 336033; e-mail: deposit@ccdc.cam.ac.uk).

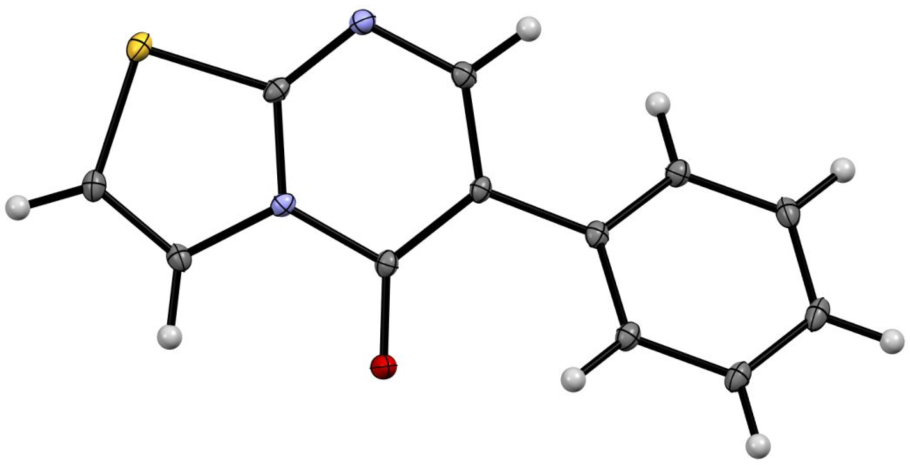

Figure S21. Molecular structure of product **3n**. Thermal ellipsoids are shown at 50% probability.

Table S5. Crystal data and structure refinement for **3n**.

Empirical formula	$C_{12}H_8N_2OS$
Formula weight	228.26
Temperature/K	150.00(10)
Crystal system	Orthorhombic
Space group	$Pna2_1$
a [Å^3]	12.7508(12)
b [Å^3]	6.4591(7)
c [Å^3]	12.2899(10)
α [°]	90
β [°]	90
γ [°]	90
V [Å^3]	1012.18(17)
Z	4
ρ_{calc} [g/cm^3]	1.498
μ [mm^{-1}]	0.295
F(000)	472.0
Crystal size/mm^3	0.4 × 0.4 × 0.2
Radiation	MoKα (λ = 0.71073)
Reflections collected	3495
Independent reflections	1871
R_{int}	0.0377
Data/restraints/parameters	1871/1/145
GOF	1.070
R_1, wR_2 [I>=2σ (I)]	0.0463, 0.0969
R_1, wR_2 (all data)	0.0570, 0.1113
$(\Delta\rho)_{max}$ [e Å^{-3}]	0.21
$(\Delta\rho)_{min}$ [e Å^{-3}]	-0.47

7. References

(1) Guchhait, S. K.; Priyadarshani, G. Pd-Catalyzed Ag(I)-Promoted C3-Arylation of Pyrido[1,2-*a*]pyrimidin-4-ones with Bromo/Iodo-Arenes. *J. Org. Chem.* **2015**, *80*, 8482–8488.

(2) Molnar, A.; Kapros, A.; Parkanyi, L.; Mucsi, Z.; Vlad, G.; Hermecz, I. Suzuki-Miyaura cross-coupling reactions of halo derivatives of 4*H*-pyrido[1,2-*a*]pyrimidin-4-ones. *Org. Biomol. Chem.* **2011**, *9*, 6559–6565.

(3) (a) Park, J. H.; Hong, S. Y.; Kim, J.; Lee, H. J.; Lee, H. H.; Kim, K. Y.; Lee, S. W.; Oh, H.-M.; Rho, M.-C.; Lee, B.-G.; Song, Y.-H. Convenient synthesis of novel phenylpyrimido[1,2-*c*]thienopyrimidinones as IL-6/STAT3 inhibitors. *Heterocycles* **2015**, *91*, 835–848. (b) Liu, S.; Fu, J.; Kamboj, R.; Jia, Q.; Wood, M.; Chowdhury, S.; Sun, J. Preparation of pyridopyrimidinone compounds useful in treating sodium channel-mediated diseases or conditions. WO2008097991 (2008); *Chem. Abstr.* **2008**, *149*, 268064.

(4) Buchmann, G.; Duchna, W. Synthesis of 1,3-disubstituted 4*H*-quinolizin-4-ones based on 2-pyridineacetonitrile. *Pharmazie*, **1968**, *23*, 301–303.

(5) Falch, E.; Natvig, T. Mass Spectra of Pyrimidines. Part II. Structure Determination of 2,3-Dihydrotriazolo[3,2-*a*]pyrimidinones. *Acta. Chim. Scand.* **1970**, *24*, 1423–1430.

(6) (a) Hermecz, I.; Vasvári-Debreczy, L. In *12.02 Bicyclic 6-6 Systems with One Bridgehead (Ring Junction) Nitrogen Atom: One Extra Heteroatom 1:0* in *Comprehensive Heterocyclic Chemistry III*; Katritzky, A. R., Ramsden, C. A., Scriven, E. F. V., Tayleor, R. J. K., Eds.; Vol 12.; Jones, K., Ed.; Elsevier: Oxford, **2008**, 94–115; and references cited therein.

(7) Rečnik, S. Svete, J.; Stanovnik, B. Coupling of Heteroaryldiazonium Tetrafluoroborates with 1,3-Dicarbonyl Compounds – Regioselective Synthesis of Alkyl 1-Heteroaryl-4-hydroxy-1*H*-pyrazole-3-carboxylates. *Heterocycles* **2002**, *57*, 2091–2106.

(8)d'Ischia, M.; Napolitano, A.; Pezzella, A. In *3.01 Pyrroles and their Benzo Derivatives: Structure* in *Comprehensive Heterocyclic Chemistry III*; Katritzky, A. R., Ramsden, C. A., Scriven, E. F. V., Tayleor, R. J. K., Eds.; Vol 3.; Jones, G., Ramsden, C. A., Eds.; Elsevier: Oxford, **2008**, 1–43; and references cited therein.

(9)Senning, A. In *3.05 Furans and their Benzo Derivatives: Structure* in *Comprehensive Heterocyclic Chemistry III*; Katritzky, A. R., Ramsden, C. A., Scriven, E. F. V., Tayleor, R. J. K., Eds.; Vol 3.; Jones, G., Ramsden, C. A., Eds.; Elsevier: Oxford, **2008**, 389–406; and references cited therein.

(10) Molina, P.; Arques, A.; Cartagena, I. In *3.09 Thiophenes and their Benzo Derivatives: Structure* in *Comprehensive Heterocyclic Chemistry III*; Katritzky, A. R., Ramsden, C. A., Scriven, E. F. V., Tayleor, R. J. K., Eds.; Vol 3.; Jones, G., Ramsden, C. A., Eds.; Elsevier: Oxford, **2008**, 625–737; and references cited therein.

(11) Bienz, S.; Bigler, L.; Fox, T.; Meier, H. In *Spectroscopic Methods in Organic Chemistry, 3rd Edition*, Thieme: Stuttgart, **2021**, 1–512.

(12) *CrysAlis PRO, Agilent Technologies UK Ltd*, Yarnton, Oxfordshire, England, **2011**.

(13) Dolomanov, O. V.; Bourhis, L. J.; Gildea, R. J.; Howard, J. A. K.; Puschmann, H. *OLEX2*: a complete structure solution, refinement and analysis program. *J. Appl. Cristallogr.* **2009**, *42*, 339–341.

(14) Sheldrick, G. M. A short history of *SHELX*. *Acta Crystallogr. A* **2008**, *64*, 112–122.

(15) Sheldrick, G. M. *SHELXT* - Integrated space-group and crystal-structure determination. *Acta Crystallogr. Sect. A Found. Adv.* **2015**, *71*, 3–8.

(16) Sheldrick, G. M. Crystal structure refinement with *SHELXL*. *Acta Crystallogr. Sect. C Struct. Chem.* **2015**, *71*, 3–8.

(17) Macrae, C. F.; Edgington, P. R.; McCabe, P.; Pidcock, E.; Shields, G. P.; Taylor, R.; Towler, M.; van de Streek, J. *Mercury*: visualization and analysis of crystal structures. *J. Appl. Crystallogr.* **2006**, *39*, 453–457.

(18) Spek, A. L. Single-crystal structure validation with the program *PLATON*. *J. Appl. Crystallogr.* **2003**, *36*, 7–13.
